# Homologous and heterologous re-challenge with *Salmonella* Typhi and *Salmonella* Paratyphi A in a randomised controlled human infection model

**DOI:** 10.1371/journal.pntd.0008783

**Published:** 2020-10-20

**Authors:** Malick M. Gibani, Celina Jin, Sonu Shrestha, Maria Moore, Lily Norman, Merryn Voysey, Elizabeth Jones, Luke Blackwell, Helena Thomaides-Brears, Jennifer Hill, Christoph J. Blohmke, Hazel C. Dobinson, Philip Baker, Claire Jones, Danielle Campbell, Yama F. Mujadidi, Emma Plested, Lorena Preciado-Llanes, Giorgio Napolitani, Alison Simmons, Melita A. Gordon, Brian Angus, Thomas C. Darton, Vincenzo Cerundulo, Andrew J. Pollard

**Affiliations:** 1 Oxford Vaccine Group, Department of Paediatrics, University of Oxford and the NIHR Oxford Biomedical Research Centre, United Kingdom; 2 Department of Infectious Diseases, Imperial College London, United Kingdom; 3 Institute for Infection and Global Health, University of Liverpool, United Kingdom; 4 Medical Research Council Human Immunology Unit, Radcliffe Department of Medicine, University of Oxford, United Kingdom; 5 Malawi-Liverpool-Wellcome Trust Clinical Research Programme, Blantyre, Malawi; 6 Nuffield Department of Medicine, University of Oxford, United Kingdom; 7 Department of Infection, Immunity and Cardiovascular Disease, University of Sheffield, Sheffield, United Kingdom; Johns Hopkins Bloomberg School of Public Health, UNITED STATES

## Abstract

Enteric fever is a systemic infection caused by *Salmonella* Typhi or Paratyphi A. In many endemic areas, these serovars co-circulate and can cause multiple infection-episodes in childhood. Prior exposure is thought to confer partial, but incomplete, protection against subsequent attacks of enteric fever. Empirical data to support this hypothesis are limited, and there are few studies describing the occurrence of heterologous-protection between these closely related serovars. We performed a challenge-re-challenge study using a controlled human infection model (CHIM) to investigate the extent of infection-derived immunity to *Salmonella* Typhi or Paratyphi A infection. We recruited healthy volunteers into two groups: naïve volunteers with no prior exposure to *Salmonella* Typhi/Paratyphi A and volunteers previously-exposed to *Salmonella* Typhi or Paratyphi A in earlier CHIM studies. Within each group, participants were randomised 1:1 to oral challenge with either *Salmonella* Typhi (10^4^ CFU) or Paratyphi A (10^3^ CFU). The primary objective was to compare the attack rate between naïve and previously challenged individuals, defined as the proportion of participants per group meeting the diagnostic criteria of temperature of ≥38°C persisting for ≥12 hours and/or *S*. Typhi/Paratyphi bacteraemia up to day 14 post challenge. The attack-rate in participants who underwent homologous re-challenge with *Salmonella* Typhi was reduced compared with challenged naïve controls, although this reduction was not statistically significant (12/27[44%] vs. 12/19[63%]; Relative risk 0.70; 95% CI 0.41–1.21; p = 0.24). Homologous re-challenge with *Salmonella* Paratyphi A also resulted in a lower attack-rate than was seen in challenged naïve controls (3/12[25%] vs. 10/18[56%]; RR0.45; 95% CI 0.16–1.30; p = 0.14). Evidence of protection was supported by a post hoc analysis in which previous exposure was associated with an approximately 36% and 57% reduced risk of typhoid or paratyphoid disease respectively on re-challenge. Individuals who did not develop enteric fever on primary exposure were significantly more likely to be protected on re-challenge, compared with individuals who developed disease on primary exposure. Heterologous re-challenge with *Salmonella* Typhi or *Salmonella* Paratyphi A was not associated with a reduced attack rate following challenge. Within the context of the model, prior exposure was not associated with reduced disease severity, altered microbiological profile or boosting of humoral immune responses. We conclude that prior *Salmonella* Typhi and Paratyphi A exposure may confer partial but incomplete protection against subsequent infection, but with a comparable clinical and microbiological phenotype. There is no demonstrable cross-protection between these serovars, consistent with the co-circulation of *Salmonella* Typhi and Paratyphi A. Collectively, these data are consistent with surveillance and modelling studies that indicate multiple infections can occur in high transmission settings, supporting the need for vaccines to reduce the burden of disease in childhood and achieve disease control.

**Trial registration** NCT02192008; clinicaltrials.gov.

## Author summary

In this study, we assessed whether previous infection with the bacteria *Salmonella* Typhi and *Salmonella* Paratyphi protected against second infections. Healthy volunteers who had been previously infected with these bacteria in earlier human challenge studies were challenged for a second time. We compared the rate of infection in the re-challenge group with healthy volunteers who were challenged for the first time. We found that previous infection was associated with a lower rate of second infections and longer time to disease but was not associated with complete protection from disease. Some individuals appeared to be more resistant to developing infection on both occasions. Previous infection with *Salmonella* Typhi did not appear to protect against later infection with *Salmonella* Paratyphi and vice-versa. Antibody responses and clinical symptoms were similar in between first and second infections. These results and future studies could help us to better understand immunity to these bacteria and help the development of new vaccines for *Salmonella* Typhi and Paratyphi.

## Introduction

Enteric fever results from infection with the typhoidal *Salmonella* serovars: *Salmonella enterica* subspecies *enterica* serovars Typhi and Paratyphi (*S*. Typhi and *S*. Paratyphi). The annual burden of enteric fever is estimated at ~14 million cases/year–the majority of which is attributable to *S*. Typhi[1]. An increasing incidence *S*. Paratyphi A disease has been reported over the past two decades, such that this serovar is now responsible for a large proportion of enteric fever cases in parts of Asia[2], including in Nepal [3], Cambodia [4,5] and China[6,7]. Comprehensive control of typhoid and paratyphoid fever is likely to require integration of initiatives to improve water quality, sanitation and hygiene, coupled with the deployment of effective vaccines[8]. Three vaccines are recommended by the WHO for typhoid fever, but no vaccines are currently available for control of paratyphoid fever[9].

Studies modelling the impact of vaccination on transmission of typhoidal *Salmonella* include infection-derived immunity as an important variable[10,11]. However, there are limited empirical data describing the extent, mechanisms and duration of immunity conferred by prior typhoid or paratyphoid infection. In high-burden settings, the incidence of typhoid disease is highest in infants and school-age children and declines with age—a pattern thought to be in keeping with acquisition of immunity through repeated exposures in childhood[1]. Modelling data suggest that multiple (~3–5) episodes of typhoid exposure are required to confer functional protection against future clinical typhoid disease[10,11]. Data from early case-series indicate that repeated typhoid infections can occur, particularly when there is exposure to a large infectious dose[12] and early human challenge studies suggest that previous typhoid fever confers moderate (~30%) but incomplete protection against subsequent disease[13]. An improved understanding of infection-derived immunity to *S*. Typhi could help to identify immunological correlates of protection and inform transmission modelling.

Much less is known regarding immunity acquired following previous paratyphoid infection. Similarly, it is unknown whether infection with *S*. Typhi confers heterologous protection against *S*. Paratyphi and vice-versa. Cross-reactive cellular and humoral immune responses can be detected *in vitro [14–16]* but it is unknown whether these responses correlate with protection. Some data indicate that the *S*. Typhi Ty21a vaccine confers moderate protection against *S*. Paratyphi B*[17]*. Less is known regarding cross-protection of Ty21a against *S*. Paratyphi A*, although it is generally considered not to confer protection to this serovar[18].*

We have previously described the establishment of *S*. Typhi and *S*. Paratyphi controlled human infection models in healthy volunteers in a non-endemic setting[19–23]. Challenge with wild-type *S*. Typhi (dose 1-5x10^4^ CFU) and *S*. Paratyphi (dose 1-5x10^3^ CFU) achieves an attack rate of 60–75% in unvaccinated individuals[19–23], whereas the attack rate, time to diagnosis and disease severity are reduced by prior-vaccination with a Vi-conjugate vaccine[22] and reduced challenge dose[19,20]. Challenge/re-challenge studies have been used extensively to investigate the extent of infection-derived immunity to a range of enteric pathogens, including *Vibrio cholerae*[24,25], enterotoxigenic *E*. *Coli*,[26] *Campylobacter jejuni*[27–29], *Shigella* spp.[30–32] and *Giardia lamblia*[33]. Such studies can offer insights into mechanisms and determinants of immunity to guide vaccination strategies.

To better characterise the effect of prior exposure on subsequent incidence of enteric fever, we performed homologous and heterologous re-challenge of participants previously enrolled in *S*. Typhi and *S*. Paratyphi challenge studies. The primary objective of the study was to compare the rate of enteric fever in re-challenge groups with naïve controls. Secondary objectives of the study were to compare the clinical features, microbiological profile and humoral immune responses between groups.

## Methods

### Ethics statement

Written informed consent was obtained from all volunteers prior to enrolment. Ethical approvals for the primary protocol, and any study amendments, were obtained from the South-Central Oxford A research ethics committee (14/SC/1204). The study was registered with clinicaltrials.gov (NCT02192008) and was performed according to the provisions of the Declaration of Helsinki and Good Clinical Practice guidelines.

### Study design & participants

We performed a participant-blinded, randomised, out-patient human challenge/re-challenge study using well-characterised wild-type strains of *S*. Typhi[19] and *S*. Paratyphi A[20]. The study was performed at the Centre for Clinical Vaccinology & Tropical Medicine (Churchill Hospital, Oxford, UK). Healthy adults aged 18–60 years, without prior residency for ≥6 months in an enteric fever endemic country, were considered eligible for enrolment. Participants with a known and documented history of typhoid vaccination with either Vi-polysaccharide or Ty21a were excluded from the naïve cohort. Key exclusion criteria included significant medical, surgical or psychiatric history, gallbladder disease and high-risk occupations as defined by National guidelines[34]. A full description of inclusion and exclusion criteria is provided in the **S1 Methods**.

We enrolled naïve volunteers with no known exposure to typhoidal *Salmonella* or previous vaccination into the naïve challenge cohort, consistent with earlier challenge studies conducted at the Oxford site [19–22].

The re-challenge cohort was comprised of volunteers who had previously participated in earlier *S*. Typhi or *S*. Paratyphi challenge studies conducted at the Oxford site[19–22]. All previous participants who had consented to be approached for future studies were contacted to consider enrolment into a re-challenge cohort. The pool of potential volunteers was limited and heterogenous—notably all eligible participants from earlier challenge studies were considered for re-challenge, regardless of previous challenge dose, outcome of primary challenge (disease vs. no-disease) or vaccination status[21,22]. In addition, participants enrolled into the naïve group of this study were eligible for enrolment into the re-challenge group after a minimum of 12 months from primary challenge (**S1 Methods**).

### Randomisation & masking

Randomisation was undertaken using the computer randomisation system Sortition (Nuffield Department of Primary Care, Clinical Trials Unit, University of Oxford). Participants in the naïve cohort were randomised 1:1 to receive either *S*. Typhi or *S*. Paratyphi. Participants in the re-challenge cohort were stratified according to previous exposure. Those with *S*. Typhi were randomised 1:1 to receive either homologous *S*. Typhi re-challenge or heterologous *S*. Paratyphi re-challenge. Those previously challenged with *S*. Paratyphi were randomised 1:1 to receive either homologous *S*. Paratyphi re-challenge or heterologous *S*. Typhi re-challenge. Varying block sizes were used. Randomisation was performed after screening investigations were completed at the time of enrolment.

Participants and laboratory staff were masked to challenge agent and group allocation until unblinding, using distinct participant IDs for clinical and laboratory staff. Microbiology staff processing blood cultures were also blinded to challenge agent allocation. Clinical staff administering the challenge agent were not blinded to challenge agent allocation.

### Procedures

Participants were challenged by oral ingestion of 1–5 × 10^4^ colony forming units (CFUs) of *S*. Typhi Quailes strain or 1–5 × 10^3^ CFUs of *S*. Paratyphi A NVGH308 strain, as previously described[20–23,35]. Two minutes prior to challenge, participants ingested a sodium bicarbonate solution (2.1g/120ml) to neutralise stomach acid. The oral challenge inoculum was administered suspended in a sodium bicarbonate solution (0.53g/30ml) and kept on ice prior to administration within two hours of preparation. Participants were reviewed twelve hours after challenge and then daily for a minimum of fourteen days, as previously described[19]. Participants completed an online diary with twice daily self-recorded temperature measurements for 21 days, covering the two-week challenge period and an additional seven days to monitor antibiotic tolerability and symptom resolution. Solicited symptoms and twice daily temperature measurements were also recorded in an electronic diary for 21 days following challenge. Symptoms were categorized as not present, mild, moderate or severe (**S1 Methods**).

We initiated antibiotic treatment when participants met the composite diagnostic criteria (see Outcomes), or at day 14 for those not diagnosed with enteric fever. From March 2015 to October 2016, the first line treatment was oral azithromycin 500mg once daily for 14 days. After October 2016, first line treatment was changed to ciprofloxacin 500mg twice-daily for 14 days. The change in first line therapy was prompted by a recommendation from the data safety monitoring committee, following review of safety data pertaining to antibiotic treatment from a parallel typhoid challenge study[22,36].

### Outcomes

The primary objective of this study was to compare the proportion of participants meeting the composite diagnostic endpoint for enteric fever (attack rate, AR) in the naïve cohorts, compared with the re-challenge cohorts. The composite diagnostic endpoint for enteric fever was defined as a temperature of ≥38°C persisting for ≥12 hours and/or *S*. Typhi/Paratyphi bacteraemia collected ≥72hours after oral challenge.

Secondary endpoints were mode of diagnosis; time to diagnosis; time to first temperature ≥38oC; time to bacteraemia; duration of bacteraemia and quantitative blood culture (**S1 Methods**). Descriptive endpoints included severe adverse events; solicited symptom profiles; proportion of participants meeting the criteria for severe enteric fever; haematological and biochemical measures; pattern of bacteraemia and pattern of stool shedding (**S1 Methods**). Blood culture samples were collected at 12 hours after challenge and daily thereafter until 96 hours post initiation of treatment. As no further blood cultures were scheduled for collection after 96 hours post-typhoid diagnosis, the analysis for duration of bacteraemia were censored at 96 hours after treatment initiation. Sample collection timepoints for bacteraemia evaluation are outlined in the **S1 Methods** and **S1 Protocol**.

Stool samples for culture, blood samples for culture (10ml), haematological and biochemical testing were processed by the local hospital’s accredited pathology laboratory as previously described[19,37]. Haematology and biochemistry laboratory samples were processed at a United Kingdom Accreditation Service (UKAS) accredited laboratory at the John Radcliffe Hospital, Oxford, UK. Stool samples were submitted for standard microbiological culture at the microbiology laboratory, John Radcliffe Hospital, Oxford University Hospital NHS Foundation Trust. Stool cultures were performed according to local procedures and based on national guidance.[38] Briefly, selenite broth was inoculated with ~1g faeces and mixed by vortex. Agar plates, including XLD, were either directly inoculated with 100μL of the suspension, or after 18–24 hours incubation at 37°C, when chromogenic agar (Salmonella Plus agar, E&O laboratories) for the detection of *Salmonella* spp, was inoculated. All negative cultures were incubated and kept for 1-week until being discarded and being reported as ‘no growth’.

Samples for assessment of antibody responses were collected at 28 and 90 days after challenge and compared with those measured at baseline. We measured specific immunoglobulin G (IgG) and IgA isotype responses to *S*. Typhi LPS (*S*. Typhosa LPS, L2387; Sigma-Aldrich, Dorset, UK); H-d antigen (University of Maryland CV0150622); *S*. Paratyphi O:2 (GSK Vaccines for global health) and H-a antigen (University of Maryland CVD1902D lot CVD141113-01) using an in-house ELISA, as previously described[19–21]. In addition, immunoglobulin G (IgG) responses to Vi were measured using a commercial ELISA kit (VaccZyme, The Binding Site Ltd, Birmingham, UK) according to the manufacturer’s instructions as previously described[22].

### Statistical analyses

Attack rates and 95% confidence intervals were calculated for each challenge group for the per-protocol population (i.e. participants who completed the fourteen-day challenge period) as the primary endpoint. Participants who were challenged but who commenced antibiotics prior to day 14 without meeting the diagnostic criteria were excluded from the primary analysis but were included in the time-to-event analysis and were censored at the time of antibiotic initiation.

No formal sample size calculations were made in the design of this study. We assumed that the attack rates in the *S*. Typhi and *S*. Paratyphi naïve challenge groups would be 60%-75%, consistent with earlier studies[19–23]. All participants who had participated in previous challenge studies and who had consented to be contacted for future studies were approached to participate in the re-challenge group. The number of volunteers enrolled into the re-challenge group was dependent on the number of participants who consented to re-challenge and who were eligible after screening. We aimed to enrol 20 volunteers into each of the *S*. Typhi and *S*. Paratyphi naïve groups, giving 95% confidence intervals (CIs) for attack rates of between 36%–81% and 46%–88% assuming measured attack rates of 60%-75%[19].

We calculated the differences in attack rates between naïve and re-challenge groups using Fisher’s exact test. Estimated protection was calculated as the percentage reduction in the re-challenge groups compared with naïve groups ([1-relative risk] x 100). We also performed a post-hoc analysis comparing attack rates in the re-challenge groups with the combined attack rates from non-vaccinated/control individuals from previous studies where challenge was performed at an equivalent dose. Sub-group analysis was performed in the re-challenge group according to prior vaccination status (no vaccine vs. any vaccine and Vi-polysaccharide, Vi-tetanus conjugate, Ty21a or MO1ZH09 vaccination) and outcome of first challenge (enteric fever diagnosis vs. no enteric fever diagnosis). We analysed factors potentially associated with a higher probability of being diagnosed when re-challenged using a multivariable log-binominal model. Predictors in the model included time since first challenge (years); challenge agent (*S*. Typhi or *S*. Paratyphi); re-challenge group (homologous vs. heterologous re-challenge); sex (male vs. female); age (years); previous typhoid vaccination (yes or no) and prior diagnosis status (diagnosed on previous challenge or not). Multivariable analysis was conducted using SAS version 9.4.

Time to diagnosis, time to first fever and time to bacteraemia were summarised using the Kaplan-Meir method, with participants censored at Day 14. Group comparisons were performed using a log-rank test.

Clinical data were recorded on a web-based database (OpenClinica Enterprise). Data analysis was performed using R version 3.6.1[39], using ggplot2[40], survminer[41] and forestplot[42] packages.

## Results

We enrolled 124 participants between 17^th^ March 2015 and 24^th^ August 2017. Nine participants withdrew prior to challenge. Three participants commenced antibiotics prior to day 14 without meeting the criteria for enteric fever diagnosis and were excluded from the primary analysis. In total, 112 participants were included in the per-protocol primary analysis (**S1 Fig**). Baseline characteristics were comparable across groups (**Table 1**).

**Table 1:** Participant characteristics OVG2014/01(PATCH) study. ST-ST Re-Challenge = Homologous re-challenge with S. Typhi following previous S. Typhi challenge. SPT-SPT Re-Challenge = Homologous re-challenge with S. Paratyphi following previous S. Paratyphi challenge. ST-SPT Re-Challenge = Heterologous re-challenge with S. Paratyphi following previous S. Typhi challenge. SPT-ST Re-Challenge = Heterologous re-challenge with S. Typhi following previous S. Typhi challenge. Prior known typhoid vaccination of any type was an exclusion criterion for enrolment into the naïve arm of the study. Participants with prior history of Vi-polysaccharide vaccination were eligible for enrolment in previous paratyphoid challenge studies. Participants with a history of Vi-polysaccharide, Vi-tetanus toxoid conjugate, Ty21a, M01ZH09 vaccination received either vaccine as part of previous human challenge studies assessing vaccine efficacy. [21, 22] Minimum interval between primary challenge and re-challenge was 12 months.

	Challenge Group						
	All	*S*. Typhi Challenge			*S*. Paratyphi (A) Challenge		
		ST Naïve	ST-ST Re-Challenge	SPT-ST Re-Challenge	SPT Naïve	SPT-SPT Re-Challenge	ST-SPT Re-Challenge
**Number**	112	19	27	10	18	12	26
**Male sex, n (%)**	71/11(63%)	14/19 (74%)	16/27 (59%)	9/10 (90%)	11/18 (61%)	6/12 (50%)	15/26 (58%)
**Age, Years, median (range)**	27.8 (18.8–60.8)	27.2 (19.6–59.7)	33.2 (21.3–60.8)	26.7(22.2–52)	27.1(18.8–42.6)	23.8 (21–44.1)	32.0 (19.3–55)
**Ethnicity, n (%)**							
***White British***	82 /112 (73%)	13 /13(68%)	19/27 (70%)	9/10 (90%)	11/18 (61%)	9/12 (75%)	21/26 (81%)
***White (Other)***	23/112 (21%)	6/19 (31%)	6/27 (22%)	1/10 (10%)	4/18 (22%)	2/12 (12%)	4/26 (15%)
***Mixed***	6/112 (5%)	0/19 (0%)	2/27 (7%)	0/10 (0%)	3/18 (17%)	0/12 (0%)	1/26 (4%)
***Asian (Indian)***	1/112 (1%)	0/19 (0%)	0/27 (0%)	0/10 (0%)	0/18 (0%)	1/12 (8%)	0/26 (0%)
**Previous travel to enteric fever endemic area, n (%)**	42/112 (38%)	5/19 (26%)	10/27 (37%)	8/10 (80%)	5/18 (28%)	8/12 (67%)	6/26 (23%)
**Alcohol consumption, any, n (%)**	92/112 (82%)	14/19 (73%)	22 /27 (82%)	9/10 (90%)	16/18 (89%)	8/12 (67%)	23/26 (88%)
**Tobacco smoker, any, n (%)**	35/112 (31%)	7/19 (37%)	9 /27 (33%)	3/10 (30%)	4/18 (22%)	5/12 (42%)	7/26 (27%)
**Previous *Salmonella* Challenge, n (%)**							
***S*. Typhi**	53/112 (47%)	-	27/27 (100%)	-	-	-	26/26 (100%)
***Challenge Dose (CFU)*: *1-5x10***^***3***^	5/112 (4%)	-	3/27 (11%)	-	-	-	2/26 (8%)
***Challenge Dose (CFU)*: *1-5x10***^***4***^	48/112 (43%)	-	24/27 (89%)	-	-	-	24/26 (92%)
***S*. Paratyphi**	22/112 (20%)	-	-	10/10 (100%)	-	12/12 (100%)	-
***Challenge Dose (CFU)*:*0*.*5–1 x 10***^***3***^	12/112 (11%)	-	-	6/10 (60%)	-	6/12 (50%)	-
***Challenge Dose (CFU)*: *1–5 x 10***^***3***^	10/113 (9%)	-	-	4/10 (40%)	-	6/12 (50%)	-
**Re-Challenge Interval, months, median (range)**^**c**^	19 (12–67.9)	-	38 (12–60.6)	17.3 (13.9–24.9)	-	17 (14.2–27.2)	28 (12.2–67.9)
**Previous enteric fever diagnosis, n (% enrolled in re-challenge groups)**	37/75(49%)	-	12/27 (44%)	7/10 (70%)	-	5/12 (41%)	13/26 (50%)
**Previous typhoid vaccination, n (%)**^**b**^ **Any Vaccine**	26/112 (23%)	0/19 (0%)	11/27 (41%)	0/10 (0%)	0/18 (0%)	0/12 (0%)	15/26 (58%)
***Vi-Polysaccharide***	7/112 (6%)	0/19 (0%)	4/27 (15%)	0/10 (0%)	0/18 (0%)	0/12 (0%)	3/27 (11%)
***Vi-Tetanus toxoid conjugate***	11/112(10%)	0/19 (0%)	3/27 (11%)	0/10 (0%)	0/18 (0%)	0/12 (0%)	8/27 (30%)
***Ty21a***	4/112 (4%)	0/19 (0%)	3/27(11%)	0/10 (0%)	0/18 (0%)	0/12 (0%)	1/27 (4%)
***M01ZH09***	4/112 (4%)	0/19 (0%)	1 /27(4%)	0/10 (0%)	0/18 (0%)	0/12 (0%)	3/26 (12%)

All challenged participants were successfully treated, with no episodes of disease relapse or recrudescence after twelve months follow up. One participant had convalescent shedding of *S*. Paratyphi in the stool following a 14-day course of ciprofloxacin. Following treatment with azithromycin, all subsequent stool samples in this participant were negative. Four serious adverse events were reported during the course of the study, of which two were considered related to challenge (**S1 Table**). Five participants met the pre-specified criteria for severe enteric fever across all challenge groups (**S2 Table**). The median interval between primary challenge and re-challenge in participants previously challenged with *S*. Typhi was 38 months (range 12–68) compared with 16.9 months (13.9–27.7) in participants previously challenged with *S*. Paratyphi (p = 0.09), reflecting the timing of previous challenge studies (**S2 Fig**).

**Fig 1:**
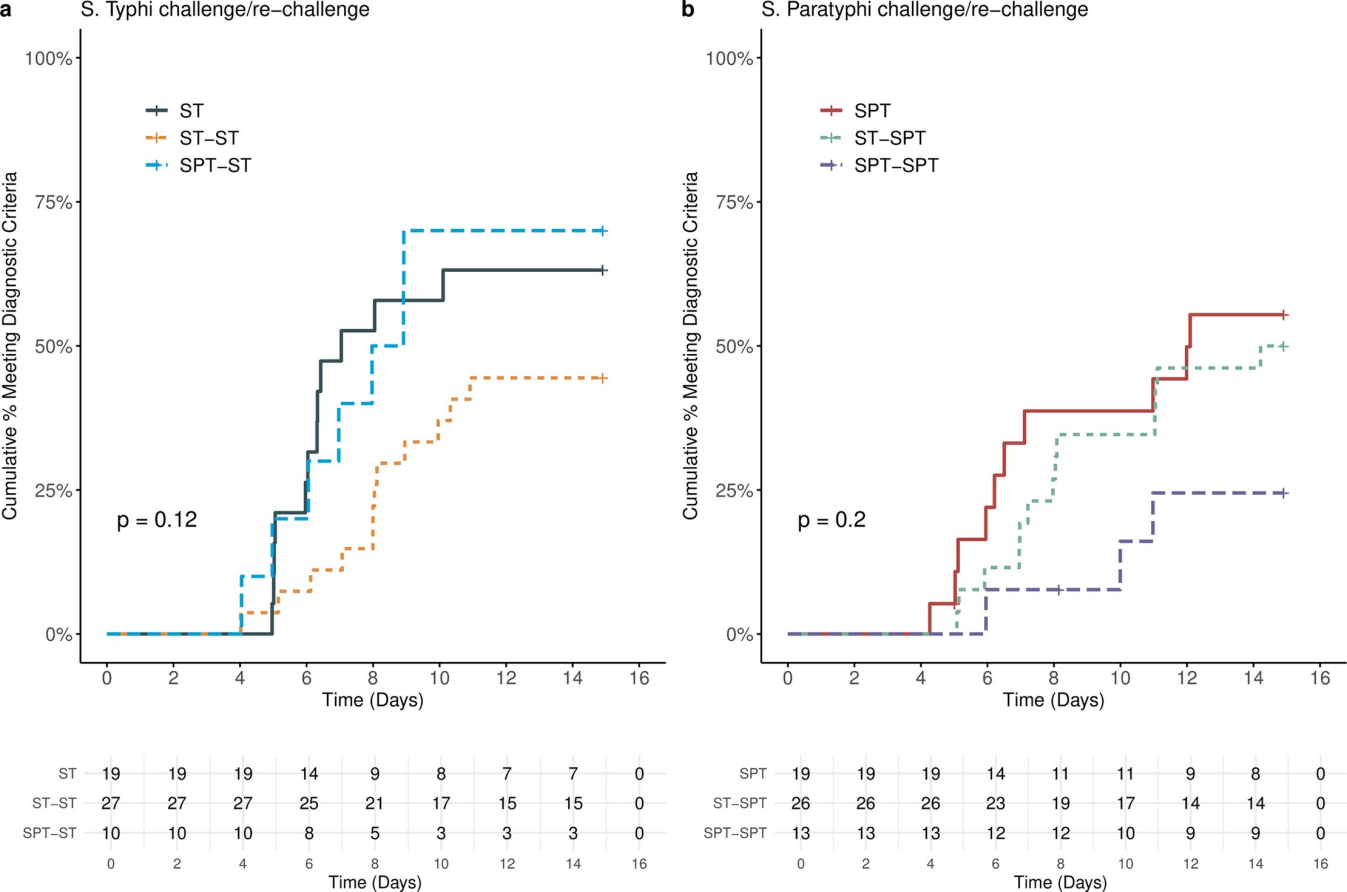
Time to diagnosis after challenge with a) *Salmonella* Typhi and b) *Salmonella* Paratyphi A. Kaplan-Meier survival cumulative incidence of participants meeting the composite diagnostic endpoint, measured from challenge agent ingestion to development of first fever ≥38°C or first positive blood culture sampling. Non-diagnosed participants censored at day 14 hours. P = log-rank test between three groups. ST = S. Typhi naïve challenge. ST-ST = Homologous Re-Challenge with S. Typhi. SPT = S. Paratyphi naïve challenge. SPT-SPT = Homologous Re-Challenge with S. Paratyphi. SPT-ST Re-Challenge = Heterologous re-challenge with S. Typhi following previous S. Paratyphi challenge. ST-SPT Re-Challenge = Heterologous re-challenge with S. Paratyphi following previous S. Typhi challenge.

**Fig 2:**
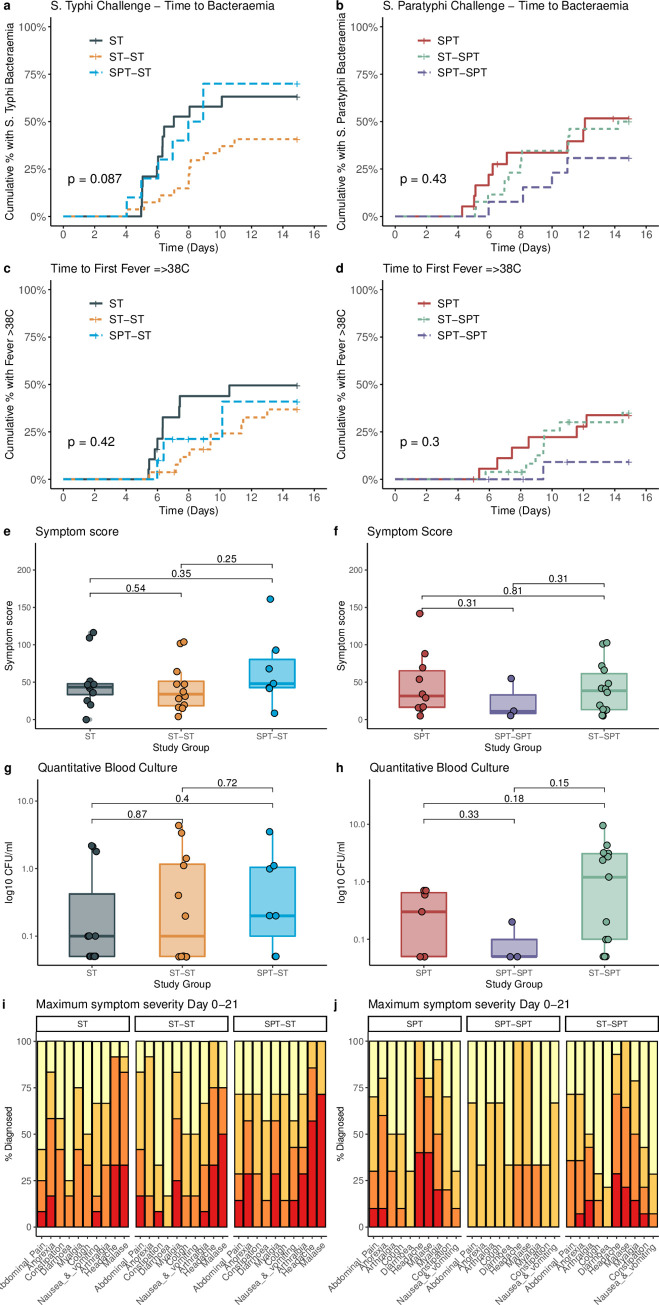
Clinical profile following Salmonella Typhi and Salmonella Paratyphi A challenge and re-challenge: Kaplan-Meier curves indicating time to bacteraemia following Salmonella Typhi (a) and Salmonella Paratyphi (b) challenge, in naïve and re-challenge participants; Time first fever >38oC following Salmonella Typhi (c) and Salmonella Paratyphi (d) challenge, in naïve and re-challenge participants. P = log-rank test between three groups;. Comparison of cumulative symptom severity scores (Day 0 to 21) in all participants diagnosed with Salmonella Typhi (e) or Salmonella Paratyphi (f) (median, interquartile range, p = Mann-Whitney U test); Quantitative blood culture at time of typhoid (g) or paratyphoid (h) diagnosis. Box plots represent median, interquartile range. Samples with no colonies were assigned a value corresponding to half the lower limit of detection (0.05 CFU/ml). p = Mann-Whitney U test; Maximum symptom severity score in participants diagnosed with typhoid (i) or paratyphoid (j) fever. Percentage of participants reporting one or more events. Symptoms were recorded using an electronic diary from Day 0 up to Day 21 post challenge. Stacked columns display percentage of participants reporting maximum symptom severity graded as mild (present but no interference with daily activity), moderate (some limitation of daily activity) or severe (unable to perform normal daily activity).

We challenged a total of 56 participants with *S*. Typhi across three groups. All participants were challenged within the target dose range of 1-5x10^4^ CFU (**Table 2**). In the *S*. Typhi homologous re-challenge group 12/27 participants (44%) met the composite primary endpoint for typhoid fever, compared with 12/19 (63%) of naïve controls, however this difference was not significant (relative risk 0.70; 95% CI 0.41 to 1.21; p = 0.24; **Table 2**). The diagnosis of typhoid fever was confirmed by a positive blood culture in 12/12 (100%) of the naïve cohort and 11/12 (92%) of the re-challenge cohort and the majority of participants in both groups were diagnosed based upon microbiological criteria (**Table 2**). The median time to diagnosis was 8 days [4–10.9] (median days [range]) in the homologous re-challenge group compared with 6.2 days [5–10.1] in the naïve group (p = 0.08 Log-rank test ST-ST vs. ST challenge) (**Fig 1A**). Homologous re-challenge was also associated with a non-significant prolonged time to bacteraemia and time to fever compared with naïve controls (**Fig 2**).

Ten participants with previous exposure to *S*. Paratyphi underwent heterologous re-challenge with *S*. Typhi, after a median interval of 17.3 months (range 13.9–24.9; **Table 2**). There was no detectable cross-protection between *S*. Typhi and *S*. Paratyphi A. Typhoid fever occurred in 7/10 (70%) of participants challenged with *S*. Typhi following prior *S*. Paratyphi challenge compared with 12/19 (63%) in the naïve cohort (RR 1.11; 95%CI 0.65 to 1.89; p = 0.99), with a comparable time to diagnosis (7.0[4.0–8.9] vs. 6.2 [5–10.1] days; p = 0.88 SPT-ST vs. ST; **Fig 1A**).

**Table 2:** Primary and secondary outcome. ST-ST Re-Challenge = Homologous re-challenge with S. Typhi following previous S. Typhi challenge. SPT-SPT Re-Challenge = Homologous re-challenge with S. Paratyphi following previous S. Paratyphi challenge. ST-SPT Re-Challenge = Heterologous re-challenge with S. Paratyphi following previous S. Typhi challenge. SPT-ST Re-Challenge = Heterologous re-challenge with S. Typhi following previous S. Paratyphi challenge.

	All	Challenge Group					
		S. Typhi Challenge			S. Paratyphi Challenge		
		ST Naïve	ST-ST Re-Challenge	SPT-ST Re-Challenge	SPT Naïve	SPT-SPT Re-Challenge	ST-SPT Re-Challenge
**Number Challenged**	115	19	27	10	19	13	27
**Included in Primary Outcome Analysis**	112	19	27	10	18	12	26
**Enteric Fever Diagnosis, n (% attack rate)**	57/112 (51%)	12/19 (63%)	12/27 (44%)	7/10 (70%)	10/18 (56%)	3/12 (25%)	13/26(50%)
**Attack rate 95% Confidence Interval, %**	41–60%	38–84%	25–65%	35–93%	31–78%	5–57%	30–70%
**Primary Clinical Diagnosis, n (% diagnosed)**	3/57 (5%)	0/12 (0%)	1/12(8%)	0/7 (0%)	1/10 (20%)	1/3 (33.3%)	0/13(0%)
**Primary Microbiological Diagnosis, n (% diagnosed)**	54/57 (95%)	12/12 (100%)	11/12 (92%)	7/7 (100%)	9/10 (90%)	2/3 (66.7%)	13/13(100%)
**Actual challenge dose administered, CFU x 10**^**3**^**, median (IQR)**	-	24.4 (21.9–29.5)	22.9 (21.3–24.4)	26.2 (21.5–29.3)	2.1 (2.1–2.6)	2.5 (2.2–2.7)	2.6 (2.4–2.8)
**Previous Typhoid Diagnosis, n (% attack rate)**	-	-	8/12 (67%)	-	-	-	10/13 (77%)
**Previous Paratyphoid Diagnosis, n(% attack rate)**	-	-	-	6/7 (86%)	-	1/5 (20%)	-
**Previous Typhoid Exposure (No Diagnosis), n (% attack rate)**	-	-	4/15 (27%)	-	-	-	3/13 (23%)
**Previous Paratyphoid Exposure (No Diagnosis), n(% attack rate)**	-	-	-	1/3 (33%)	-	2/7 (29%)	-
**Previous Vaccine Diagnosed, (% attack rate)**							
**None**	23/50 (46%)	NA	7/16 (44%)	7/7 (100%)	NA	3/12 (25%)	6/10 (60%)
**Any**	12/26 (46%)	NA	5/11 (45%)	0/0 (0%)	NA	0/0 (0%)	7/15 (47%)
***Vi-PS***	3/7 (43%)	NA	2/4 (50%)	0/0 (0%)	NA	0/0 (0%)	1/3 (33%)
***Vi-TT***	5/11 (45%)	NA	0/3 (0%)	0/0 (0%)	NA	0/0 (0%)	5/8 (63%)
***Ty21a***	2/4 (50%)	NA	2/3 (67%)	0/0 (0%)	NA	0/0 (0%)	0/1 (0%)
***M01ZH09***	2/4(50%)	NA	1/1 (100%)	0/0 (0%)	NA	0/0 (0%)	1/3 (33%)
**Time to diagnosis, Days, median (range)**	7.0(6.0–8.9)	6.2 (5.0–10.1)	8.0(4.0–10.9)	7.0(4.0–8.9)	6.4 (4.3–12.1)	9.4 (6.0–11.0)	8.0 (5.1–14.2)
**Time to first fever >38°C, Days, median (range)**	8.43 (6.4–11)	7.3(5.4–10.6)	10.2 (5.5–13.0)	6.4 (6.0–10.1)	7.4(5.0–12.2)	9.44 (9.4–10.0)	9.5 (5.8–14.5)
**Severe Enteric Fever (Any), n**	5/57 (9%)	2/12 (17%)	0/12 (0%)	1/10 (10%)	0/10 (0%)	0/3 (0%)	2/13 (15%)
***Systolic blood pressure ≤ 85mmHg***	0	0	0	0	0	0	0
***Oral Temperature ≥ 40°C***	0	0	0	0	0	0	0
***Significant lethargy or confusion***	0	0	0	0	0	0	0
***Gastrointestinal bleeding and/or perforation***	0	0	0	0	0	0	0
***Grade 4 laboratory abnormality***	5	2	0	1	0	0	2
**Microbiology—Blood culture**							
**Time to bacteraemia, Days, Median (IQR)**	7.0 (5.9–8.9)	6.2 (5.0–6.7)	8 (6.1–9.0)	7 (5.0–8.9)	6.2 (5.1–11.0)	7 (6–11)	8 (7–11)
**Duration of bacteraemia,Hrs, Median (IQR)**	81.4 (60.2–96)	24.5(4.49–76.3)	24 (15.1–68.4)	92 (88.4–96)	93.7 (67.5–96)	72.8 (49.5–96)	74.8 (45.6)
**Positive at ED + 96 hours, n (% diagnosed)**	21/57 (37%)	2/12 (17%)	1/12 (8%)	6/7 (86%)	5/10 (50%)	1/3 (33%)	6/13(46%)
**Quantitative blood culture, CFU/ml, Median (IQR)**	-	0.1(0–1.8)	0.1 (0–1.3)	0.2 (0–1.1)	0.3 (0–0.7)	0 (0–0.2)	1.2 (0.05–3.15)

We challenged a total of 59 participants with *S*. Paratyphi A in three groups (**Table 2**). Three participants commenced antibiotics prior to meeting the diagnostic endpoint before day 14 and were excluded from analysis of the primary endpoint (**S1 Fig**). All participants were challenged within the target dose range of 1-5x10^3^ CFU (**Table 2**). The observed rate of paratyphoid infection in the *S*. Paratyphi homologous re-challenge group was 3/12 (25%) compared with 10/18 (56%) in the naïve group, however this difference was again not significant (RR 0.45; 95% CI 0.16 to 1.30; p = 0.14). In addition, the median time to diagnosis was longer in the *S*. Paratyphi homologous re-challenge group at 9.4 days [6.0–11.0] (median [range]) group, compared with 6.4 days (4.3–12.1) in the naïve group, but did not meet significance (p = 0.09 SPT-SPT vs. SPT; **Fig 1B**).

A total of 26 participants with previous exposure to *S*. Typhi underwent heterologous re-challenge with *S*. Paratyphi. The median interval between primary challenge and re-challenge was 28 months (range 12.2–67.9). The attack rate in the *S*. Paratyphi heterologous re-challenge group was not reduced compared with naïve controls (13/26 (50%) vs. 10/18 (56%); RR 0.90; 95% CI 0.51 to 1.58; p = 0.77; ST-SPT vs. SPT; **Fig 1B**). In keeping with this, there was no significant difference in time to bacteraemia and time to first fever following heterologous re-challenge (**Fig 2**).

Regardless of the disease definition that was applied, we observed no significant difference in the attack rate of enteric fever between naïve and re-challenge groups (**S4 Table**).

As the main comparisons between naïve and re-challenge cohorts had low statistical power with wide confidence intervals, we performed a post-hoc analysis comparing the rate of typhoid infection in the re-challenge cohort with the combined attack rate from all naïve volunteers enrolled in all challenge studies between 2011 and 2018, including naïve participants from this study[20–23,35]. The observed attack rate across all naïve volunteers challenged with *S*. Typhi (n = 121) was consistent from study-to-study with an average attack rate of 69% (95%CI 60–77% **S3 Fig**).When compared with all unvaccinated historical controls challenged in Oxford, the rate of typhoid infection was significantly reduced in the *S*. Typhi re-challenge group compared with naïve historical controls, corresponding with a 36% relative risk reduction (84/121 [69%] vs. 12/27 [44%]; RR 0.64 [0.41–0.99]; p = 0.02 Fisher’s exact test). The time to diagnosis was also prolonged in the typhoid re-challenge group when compared with all naïve historical controls but did meet significance (median days [range] 7[4–14] vs. 6.2 [5–10.1]; p = 0.06 –**S4A Fig**). When we included all naïve participants from this and earlier *S*. Paratyphi studies[20] in a post-hoc analysis (n = 39), previous challenge with *S*. Paratyphi was associated with an estimated 57% relative reduction in the rate of paratyphoid disease and prolonged time to diagnosis, but this difference did not meet significance (3/12[25%] vs. 22/38 [58%]; RR 0.43 [0.15–1.19]; p = 0.1; **S4 Fig**).

In order to determine if prior exposure was associated with an altered clinical phenotype, we compared solicited symptoms between naïve and re-challenge groups. Homologous *S*. Typhi and Paratyphi re-challenge was associated with a non-significant prolongation in the time to bacteraemia and time to first fever compared with naïve controls (**Fig 2A–2D, S1 Data**). The most common symptoms reported by all participants diagnosed with enteric fever were headache (97%), malaise (90%), anorexia (78%) and myalgia (78%) (**Fig 2**). Symptom profiles were broadly similar between all study groups, with the exception of participants diagnosed in the *S*. Paratyphi homologous re-challenge group who reported fewer severe symptoms. There was no significant difference in the proportion of participants recording any fever between naïve and re-challenge groups (**S4 Table**). Haematological and biochemical parameters were comparable between groups (**S5 Fig**). Overall, this suggests that prior exposure was not associated with an altered clinical phenotype within the context of the model.

In order to determine if previous challenge was associated with an altered microbiological profile, we compared the pattern of bacteraemia and stool shedding between all challenge groups. We observed no significant difference in the number of colony forming units at the time of typhoid or paratyphoid diagnosis (**Fig 2**). The pattern of bacteraemia following challenge with S. Typhi and Paratyphi is illustrated in **S6 Fig**. In participants who met the composite criteria for typhoid or paratyphoid fever, the diagnosis was confirmed by positive blood culture in 30/31 (97%) and 25/27 (93%) cases respectively. Participants who met the diagnostic criteria for enteric fever were significantly more likely to have at least one positive stool culture than participants who did not develop disease (36/58 [62%] vs. 18/75 [24%]; RR 2.59 [95%CI 1.67–4.10]; p<0.0001 –**Table 2**, **S7 Fig).**

Participants enrolled into the re-challenge cohorts were heterogeneous with respect to several variables that might impact the outcome of re-challenge. These included vaccination status, outcome of primary challenge (disease vs. no disease) and interval between primary and secondary challenge (**Table 1**). We analysed sub-groups within the re-challenge cohort to determine which factors might impact the response to re-challenge. Within the re-challenge cohort, participants with any history of typhoid vaccination had an equivalent attack-rate on re-challenge compared with unvaccinated participants (AR 12/26[46%] vs. 23/50[46%]; **Table 2**).

Of the 75 participants analysed in all re-challenge groups, 37 (49%) had been previously diagnosed with enteric fever on primary exposure. Individuals who did not develop enteric fever at the time of their first challenge had a significantly lower attack rate when they were re-challenged, as compared with those who had been diagnosed with enteric fever after their first exposure (10/38 [26%] vs. 25/37 [68%]; RR 0.38, 95%CI 0.22–0.69; p = 0.0005; **Table 3**). When analysed by sub-group, participants who did not develop typhoid on primary exposure were less likely to develop typhoid on re-challenge (4/15 vs. 8/12; RR 0.40, 95%CI 0.16 to 1.01 p = 0.06; **Table 3**). The protection conferred to these individuals in their primary challenge study may have resulted from prior vaccination, as the *S*. Typhi re-challenge group included some individuals who had received typhoid vaccines, as well as those who had received a placebo or no vaccine. To explore this, we repeated the analysis in the *S*. Typhi homologous re-challenge group excluding previously vaccinated individuals and observed a similar trend (2/9[22%] vs. 5/7[71%]; RR0.31 [0.08–1.15]; p = 0.13; **Table 3**). In the multivariable model, participants who did not develop disease on primary exposure were also less likely to be diagnosed on re-challenge after adjusting for prior vaccine status, time since primary challenge, age, sex and challenge agent/group. No other factors were independently associated with diagnosis in this model (**Table 4**).

Compared with naïve controls, participants in the *S*. Typhi homologous re-challenge group who did not develop typhoid on primary exposure were less likely to develop typhoid on re-challenge than naïve controls (4/15 vs. 12/19; RR 0.42 (0.17–1.05); p = 0.04; **S8 Fig, S9 Fig**).

**Table 3:** Comparison of attack rates in re-challenge cohorts according to outcome of primary challenge (n^No previous disease^/n^previous disease^).

	No Previous Disease on Primary Challenge	Previous Disease on Primary Challenge	RR (95% CI)	P
**All**	10/38 (26%)	25/37(68%)	0.38 (0.22–0.69)	0.0005
**ST-ST**	4/15 (27%)	8/12(67%)	0.40 (0.16–1.01)	0.06
*ST-ST*: *No previous vaccine*	2/9 (22%)	5/7 (71%)	0.31 (0.08–1.15)	0.13
*ST-ST*: *previous vaccine*	2/6 (33%)	3/5 (60%)	0.56 (0.14–2.12)	0.57
**SPT-ST**	1/3 (33%)	6/7(86%)	0.39 (0.08–1.98)	0.18
**SPT-SPT**	2/7(29%)	1/5(20%)	1.43 (0.17–11.76)	0.99
**ST-SPT**	3/13(23%)	10/13(77%)	0.30 (0.11–0.85)	0.02

**Table 4:** Multivariable log-binomial model displaying adjusted relative risk of diagnosis in re-challenged participants (n = 75). ST = *S*. Typhi, SPT = *S*. Paratyphi.

Parameter		Adjusted RR (95% CI)	p
**Years since primary challenge**	*Per Year*	1.01(0.85–1.20)	0.91
**Challenge agent**	*ST*	1.21 (0.74–1.97)	0.44
	*SPT*	*Ref*.	-
**Challenge group**	*Homologous re-challenge*	0.75 (0.45–1.23)	0.25
	*Heterologous re-challenge*	*Ref*.	-
**Sex**	*Male*	1.17 (0.69–1.98)	0.55
	*Female*	*Ref*.	-
**Age**	*Per year older*	1.01 (0.98–1.04)	0.41
**Previous diagnosis**	*Yes*	2.09 (1.25–3.48)	0.0048
	*No*	*Ref*.	-
**Previous vaccine (any)**	*Yes*	1.05 (0.70–1.60)	0.80
	*No*	*Ref*.	-

**Fig 3:**
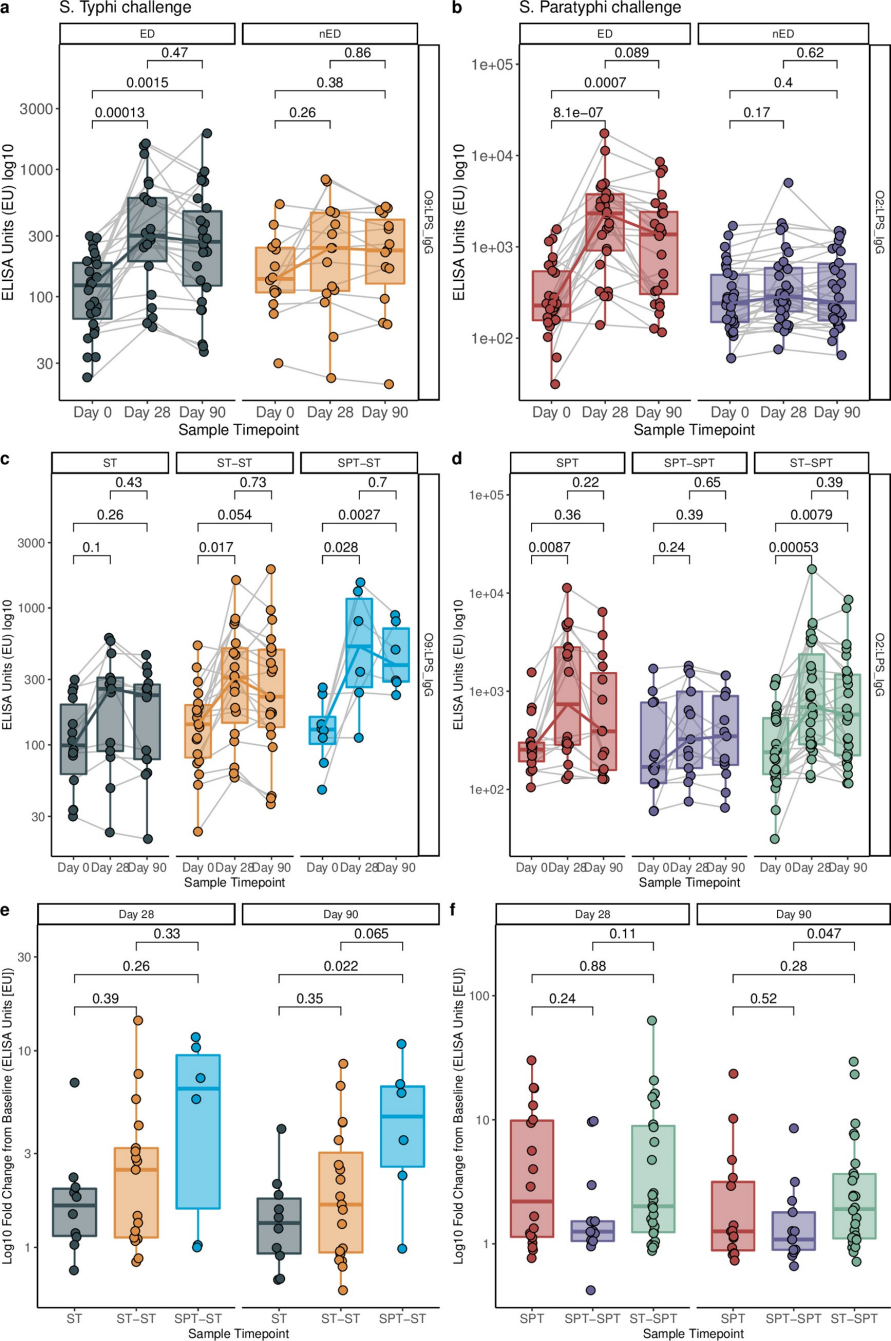
Antibody response to *Salmonella* Typhi and *Paratyphi* A challenge/re-challenge. Serum IgG response to O9:LPS following *Salmonella* Typhi challenge (a) and O2:LPS following Salmonella Paratyphi challenge (b) grouped according to outcome (ED = Met criteria for enteric fever diagnosis. nED = Did not meet criteria for enteric fever diagnosis); Comparison of serum IgG response to O9:LPS (c) and O2:LPS (d) according to challenge/re-challenge group. Coloured lines connect median values for each timepoint. Grey lines connect paired samples across timepoints; p = Wilcoxon signed rank test; Magnitude of anti-O9:LPS IgG following *Salmonella* Typhi challenge (e) and anti-O2:LPS IgG following *Salmonella* Paratyphi challenge (f) expressed as log10 fold change from baseline at Day 28 and Day 90, p = Mann-Whitney U test; ST = S. Typhi naïve; ST-ST = Homologous S. Typhi re-challenge. SPT-ST = Heterologous S. Typhi re-challenge. SPT = S. Paratyphi naïve; SPT-SPT = Homologous S. Paratyphi re-challenge. ST-SPT = Heterologous S. Paratyphi re-challenge.

Baseline antibody levels to O-, H- and Vi-antigens were comparable between naïve and re-challenge participants, and did not differ between those who went on to develop disease compared with those who did not (**S10 and S11 Figs**). Consistent with previous studies, serum antibody to serovar-specific O-antigens were more pronounced in individuals who developed typhoid and paratyphoid fever, as compared with those who did not develop disease after challenge (**Fig 3A and 3B**). Challenge with *S*. Typhi was not associated with a significant anti-Vi and anti-Hd IgG response (**S12 Fig**). There was no demonstrable antibody booster effect when we compared the fold-change of anti-O serum naïve and re-challenge groups were compared (**Fig 3**).

## Discussion

In this study, we have described the application of a controlled human infection model to study the impact of prior exposure to *S*. Typhi and Paratyphi on the rate of infection on re-challenge. Overall, our data are consistent with surveillance data and modelling studies, which suggest that a single prior exposure to *S*. Typhi/Paratyphi A induces partial but incomplete protection against subsequent disease. Intriguingly, we observed that individuals who did not develop enteric fever on primary exposure were more likely to be protected on re-challenge, compared with individuals diagnosed on primary exposure. A major caveat to these observations is the low statistical power owing to small sample size within each sub-group. This notwithstanding, these data suggest that a single previous exposure to *S*. Typhi and *S*. Paratyphi was associated with an approximately 36% and 57% reduced risk of typhoid or paratyphoid disease on re-challenge, respectively. Heterologous re-challenge with *S*. Paratyphi or Typhi was not associated with a reduced rate of infection. In those participants who did develop disease, the clinical and microbiological features of naïve and re-challenge participants were indistinguishable.

The protective effect of prior *S*. Typhi challenge was notably similar to that observed in previous studies describing infection-derived immunity to typhoid fever in different epidemiological settings and patient populations. Marmion and colleagues describe a 35% relative risk reduction in previously infected patients in the context of two consecutive typhoid outbreaks[12], and Dupont and colleagues report a 30% relative risk reduction in a human challenge study[13]. No participants (0/3) who had previously received a Vi-tetanus toxoid conjugate vaccine[22] developed typhoid fever on re-challenge. Comparisons between historical and contemporary typhoid challenge studies are complicated by key methodological differences, particularly relating to study population, challenge dose and mode of administration. New insights from our study include a substantially longer interval between challenge and re-challenge compared with previous studies (median 38 months vs. ≤12months), and the inclusion of participants who did not develop disease on primary exposure[12,13]. Overall, these data support the view that a single episode of typhoid infection induces moderate, but incomplete, protection against subsequent disease. Given this, it is reasonable to recommend that a history of prior typhoid disease should not preclude typhoid vaccination where it is clinically indicated—either when typhoid vaccines are used as part of vaccine campaigns in endemic countries or for travellers to high-risk areas.

Prior *S*. Paratyphi exposure was associated with a slightly greater protective effect within the context of the challenge model (57%) but did not meet the significance threshold due to the small sample size within the homologous re-challenge group (n = 12). We also observed a longer time to disease onset in the *S*. Paratyphi re-challenge group compared with the naïve group, however the prolongation was not significant. The estimate of protective effect from prior paratyphoid exposure is comparable to that conferred by Vi-conjugate vaccines following typhoid challenge [22] and is likely to be an underestimate owing to the strict diagnostic endpoint applied.[22] These observations raise the possibility that prior paratyphoid infection could confer at least partial immunity against re-infection and would be supportive of an approach to test live-attenuated oral paratyphoid vaccines in this model (e.g. CVD-1902[43]). Differences in the magnitude of protection associated with prior *S*. Typhi or *S*. Paratyphi challenge might be explained by differences between the serovars (e.g. lack of Vi-capsule expression by S. Paratyphi A), challenge dose (10^3^ vs 10^4^CFU) or a longer interval between challenge episodes (38 vs. 17 months) associated with waning immunity. The precision of these observations is limited by the small sample size in each study sub-group and will require further validation in field studies or future challenge studies.

This study also represents the first description of heterologous re-challenge with *S*. Typhi and *S*. Paratyphi A with the aim of studying cross-protection between these closely related serovars.[44] When individuals previously challenged with *S*. Typhi were re-challenged with *S*. Paratyphi A, the attack rate was comparable with that in naïve controls (52% vs. 56%; RR0.93 [0.54–1.68]; p = 0.99). Patients with typhoid fever and individuals vaccinated with oral typhoid-vaccines have detectable cross-reactive humoral immune responses against Paratyphi A in vitro.[14–16] However, field studies in highly endemic areas have shown that Ty21a does not appear to protect against *S*. Paratyphi A,[18] but there is evidence for moderate protection against *S*. Paratyphi B from a retrospective analysis (49% efficacy; 95%CI 8–73%)[17]. Whilst these comparisons are also underpowered, these data suggest that prior exposure to *S*. Typhi is not associated with a significant protection against S. Paratyphi A infection within the context of the challenge model.

The effect of previous *S*. Paratyphi A infection on protection against *S*. Typhi disease has been less extensively studied. The attack rate in the *S*. Typhi heterologous re-challenge group was similar to naïve controls challenged with *S*. Typhi, albeit in a small sample of volunteers (n = 10). As these serovars are co-endemic and share a primary mode of transmission, an improved understanding of cross-reactive immune responses between these two serovars will be important in future disease control efforts–particularly in the context of the impending deployment of Vi-conjugate vaccines that offer no protection against *S*. Paratyphi A.

Whilst in our study previous exposure to *S*. Typhi and Paratyphi A was associated with a moderate protection against disease on re-challenge, the clinical presentation in re-challenged participants was comparable to those with no prior exposure. Modelling studies postulate a spectrum of hypothetical immune states following prior typhoid infection, ranging from sterile immunity through to clinical immunity to complete susceptibility[10,11]. In particular, a state of clinical immunity is proposed to be characterised by milder infection, with or without shedding, that is less likely to require clinical attention. There are no validated metrics of typhoid/paratyphoid severity. When measured using a range of clinical and microbiological endpoints, we observed no difference in the observable clinical syndrome between naïve and re-challenge groups, although early treatment initiation limits the conclusion that can be drawn from these endpoints. Data from this study suggest that prior exposure is not demonstrably associated with an attenuated clinical phenotype and clinical immunity compared with naïve exposure, although the significance of this to field settings is as yet unclear.

Intriguingly, we observed that individuals who did not develop enteric fever on primary exposure were more likely to be protected on re-challenge, compared with individuals diagnosed on primary exposure. Only 49% of those recruited into the re-challenge groups had developed disease on primary exposure, suggesting a slight recruitment bias towards those who were protected on primary exposure. Host factors might explain why apparent susceptibility to typhoid infection differs between individuals. For example, genome wide association studies in Vietnam and Nepal have identified variation at HLA-DRB1 to be strongly associated with resistance to enteric fever[45]. Additional risk factors associated with susceptibility to enteric fever include variations in the *cftr* locus [46,47] and carriage of *Helicobacter pylori*[48,49]. The effect of the host microbiome on susceptibility to enteric fever is currently the subject of further study[50].

It is unclear if the development of clinical immunity following *S*. Typhi/Paratyphi challenge requires the development of clinical disease/bacteraemia, or whether immunity can develop following asymptomatic exposure. Our previous studies, have shown that the majority of participants challenged with *S*. Typhi produce a transient peak in cytokine production as early as 12 hours after challenge, which is independent of the subsequent development of typhoid disease, suggesting that early innate interactions occur in most challenged participants.[51] However, in the challenge model, effective priming of the adaptive immune system appears to require the development of symptomatic infection and/or bacteraemia, as individuals who did not develop typhoid/paratyphoid disease after challenge failed to mount a humoral or cellular response to *S*. Typhi/Paratyphi antigens[19,20,52–56].

These observations raise the possibility that the protection observed following homologous re-challenge is not mediated by a classical adaptive memory immune response following primary exposure. Differences in infection rates after re-challenge may instead be accounted for by inter-host variation in genetic or epigenetic factors impacting innate immune responses or the development of trained innate immunity after primary infection[57]. Alternatively, protection may be mediated by adaptive immune responses that have yet to be measured–such as those mediated by T- and B-cells at the gastrointestinal mucosa[58].

We acknowledge the limitations of our experimental approach. The study was broadly underpowered to detect anything other than large differences in attack rates between naïve and re-challenge groups and the protective effects were non-significant, with wide confidence intervals, due to the small sample size in the re-challenge arms. We cannot exclude that any apparent protective effect driven by participants in the re-challenge arm who were vaccinated in earlier studies, although the sub-group analysis does not support this. No formal sample size calculations were made in the design of this study as the size of the re-challenge cohort was dependent on the number of participants who were willing and consented to take part in a second challenge. However, we contend that the protective effect of prior exposure may represent an underestimate of the likely protective effect observed in field settings. As an example, the efficacy of typhoid conjugate vaccine in the human challenge model was 52%[22] as compared with 1-year efficacy of 81.6% in an randomised control trial in Nepal[59], which likely reflects the stringent diagnostic criteria and regularity of blood culture in the challenge model. Safety considerations in the design of this study, including early initiation of rescue therapy, limit the extent that our findings can be extrapolated to endemic settings[60]. It is plausible that some individuals who did not develop enteric fever during the 14-day observation period may have progressed to symptomatic disease/bacteraemia had antibiotic treatment not been initiated in all participants. Conversely, some participants who were diagnosed based on bacteraemia in the absence of clinical signs of disease may not be representative of typhoid fever in the field–indeed, spontaneous clearance of asymptomatic *S*. Typhi bacteraemia in the absence of treatment has been described[61].

As only a single strain from each serovar was used in this study we cannot conclusively rule out a strain-specific effect for our observations. However, the NVGH308 strain is a recent clinical isolate from a symptomatic case with bacteraemia, and is closely related to currently circulating strains [20]. The Quailes strain of *S*. Typhi is also related to other known disease causing isolates[19,62]. Both *S*. Typhi and *S*. Paratyphi A are clonally monomorphic pathogens containing limited genomic variation^17^, suggesting that the pathogenicity and immune response to both the Quails and NVGH308 strains should translate to other wild-type strains.

It could be speculated that a larger protective effect would have been observed had we employed a shorter interval between challenge episodes. Other re-challenge studies of enteric pathogens have typically used an interval of 1-≤12months [24–33]. The minimum interval between primary challenge and re-challenge in this study was set at 12 months, which was defined primarily to ensure the safety and comfort of study participants in mind.

The overarching aim of this study was to investigate the mechanisms and determinants of immunity following natural infection with *S*. Typhi and Paratyphi. To address this within the context of the challenge model, the study incorporated a re-challenge group to approximate individuals with prior immunological priming. Re-challenged participants were compared with ostensibly “immunologically naïve” individuals from a non-endemic country with no known prior exposure to typhoidal Salmonella. In reality, both the naïve and re-challenge groups are internally heterogenous with respect to baseline immune status, with a degree of overlap between groups. This was the illustrated by the elevated baseline anti-Vi IgG in the naïve cohort (**S10 Fig**), presumably reflecting undisclosed/undocumented travel vaccination or cross-reactive immune responses from exposure to other serotypes. Re-challenge studies aim to approximate of the extent of immunity conferred by prior exposure to a pathogen. However, a single discrete exposure to a high pathogen load is unlikely to be representative of the exposure dynamics in typhoid and paratyphoid fever in endemic settings, where immunity is thought to be acquired through multiple exposure episodes over time. In addition, the age-distribution of participants enrolled in the challenge studies (**Table 1**) was not representative of those who acquire typhoid/paratyphoid fever in many endemic settings. In order for findings from challenge studies to inform studies of transmission and vaccine development in countries with the highest burden of disease, it will be necessary to compare findings from challenge studies with naturally occurring disease in an endemic setting. To that end, prospective studies comparing the response to natural infection in typhoid endemic countries are ongoing.

In this study we have described the application of a controlled human infection model to study infection derived immunity to *S*. Typhi and Paratyphi infection. By comparing groups of individuals who have been previously exposed to these pathogens, this approach aims to assess the contribution of infection-derived protection in the prevention (or otherwise) of subsequent disease. This approach has proven valuable in vaccine development for other enteric pathogens, such as *Shigella* spp[63] and *Vibrio Cholerae*[24,25], and could potentially accelerate the development of vaccines for *S*. Paratyphi A. Improved understanding of infection-induced immunity could provide valuable data to refine modelling of transmission dynamics and vaccine impact measures, in addition to aiding the identification of correlates of protection to expedite vaccine development for *S*. Typhi and Paratyphi.

## Supporting information

S1 CONSORT Checklist (DOC)

S1 Table Serious adverse events OVG2014/01 study. (DOCX)

S2 Table Participants meeting pre-specified criteria for severe enteric fever. Hypokalaemia was observed in four participants, which was attributed to pseudohypokalaemia resulting from delayed sample transport over periods of high ambient temperature.
(DOCX)

S3 Table Number (%) of participants enrolled for re-challenge from previous challenge studies. Participants from the OVG2014/01 (n = 113) and OVG2014/08 (n = 103) studies were eligible for re-challenge after 12 months had elapsed from their primary challenge. As these studies were running contemporaneously, only a proportion of participants from these studies were eligible for re-challenge by the end of recruitment.
(DOCX)

S4 Table Attack rates according to alternative diagnostic criteria. ST = S. Typhi. SPT = S. Paratyphi A. p = Fishers exact test.
(DOCX)

S5 Table *Salmonella* Typhi and Paratyphi controlled human infection studies conducted in Oxford 2011–2017. (DOCX)

S1 Fig Study profile. Participants were recruited into one of three study groups defined *a priori* according to prior challenge status.
(PDF)

S2 Fig Recruitment into re-challenge cohort. Density plot according to previous challenge agent allocation. Vertical lines represent median re-challenge interval for participants previously challenged with S. Typhi (orange) and S. Paratyphi (grey)
(PDF)

S3 Fig Combined Attack Rates in S. Typhi and S. Paratyphi challenge studies. Forest plot illustrating attack rates in naive cohorts of S. Typhi (top) and S. Paratyphi (bottom) challenge studies. Heterogeneity I-squared = 0, test of homogeneity (Q statistic) gives p value for 0.78 (no evidence the proportions vary).
(PDF)

S4 Fig Time to Diagnosis after homologous re-challenge and combined naïve historical controls. Cumulative incidence of typhoid (i) and paratyphoid A (ii) fever after challenge in naïve (ST& SPT) and homologous re-challenge (ST-ST & SPT-SPT) groups. Time to composite diagnostic endpoint, measured from challenge agent ingestion to development of first fever ≥38°C or first positive blood culture sampling. Non-diagnosed participants censored at day 14 hours. P value from log-rank test comparing ST = S. Typhi naïve challenge. ST-ST = Homologous Re-Challenge with S. Typhi. SPT = S. Paratyphi naïve challenge. SPT-SPT = Homologous Re-Challenge with S. Paratyphi.
(PDF)

S5 Fig Haematology (a & b) and Biochemistry (c & d) laboratory parameters post challenge according to diagnosis status. Timepoints in a and c are normalised to the day of diagnosis (ED day = 0). Timepoints b and d in non-diagnosed participants represent day of sample collection. Box-and whisker plots represent median and interquartile range. Solid coloured lines link median value at each time point. Grey lines connect paired data points from the same individuals. Units: Haemoglobin = (g/dL); Haemoglobin change g/dL compared with Hb Day 0; Haematocrit(L/L); White cell count/Neutrophil count/Lymphocyte count/Eosinophil count/Monocyte count = cells x 10^9^/L; Urea = mmol/L; Creatinine = mg/L; Na^+^/K^+^ = mEq/L; C-reactive protein = mmol/l; Bilirubin = umol/l; ALT–IU/l; ALP = U/L; Albumin = g/L.
(PDF)

S6 Fig Pattern of bacteraemia following *S*. Typhi (A) and *S*. Paratyphi A (B) challenge. Each row corresponds to an individual participant. Grey squares = Negative sample, Red squares = Positive blood culture, White squares = No sample collected. Tx = Day of treatment initiation. Participants above the dotted line did not meet the composite criteria for typhoid or paratyphoid diagnosis.
(PDF)

S7 Fig Pattern of stool shedding after S. Typhi and S. Paratyphi challenge. Each row corresponds to an individual participant. Grey squares = Negative sample, Brown squares = Positive stool culture, White squares = No sample collected. Tx = Day of treatment initiation. Participants above the dotted line did not meet the composite criteria for typhoid diagnosis.
(PDF)

S8 Fig Sub-Group analysis OVG2014/01 study. Forest plot comparing relative risk of typhoid or paratyphoid diagnosis following re-challenge compared with naïve controls from OVG2014/01 study. Box-plots represent relative risk and 95% confidence intervals scaled according to size of sub-group. Diamonds represent combined relative risk of diagnosis in each of the re-challenge cohorts. P = Fishers exact test.
(PDF)

S9 Fig Sub group analysis. Forest plot comparing relative risk of typhoid or paratyphoid diagnosis following re-challenge compared with naïve and unvaccinated controls challenged with wild-type strains in all challenge studies[20–23,35]. Box-plots represent relative risk and 95% confidence intervals scaled according to size of sub-group. Diamonds represent combined relative risk of diagnosis in each of the re-challenge cohorts. P = Fishers exact test.
(PDF)

S10 Fig Baseline (Day 0) serum anti-O9:LPS (a) anti-Hd (b) and anti-Vi (c) IgG in participants challenged with S. Typhi, grouped according to (i) outcome of challenge (ii) challenge group and (iii) outcome of previous challenge. p = Mann-Whitney U test two sided; Box plots display median, interquartile range; ST = S. Typhi naïve; ST-ST = Homologous S. Typhi re-challenge. SPT-ST = Heterologous S. Typhi re-challenge. ED = Met criteria for enteric fever diagnosis. nED = Did not meet criteria for enteric fever diagnosis.
(PDF)

S11 Fig Baseline (Day 0) serum anti-O2:LPS IgG (a) and IgA in participants challenged with *S*. Paratyphi, grouped according to (i) outcome of challenge (ii) challenge group and (iii) outcome of previous challenge. p = Mann-Whitney U test two sided; Box plots display median, interquartile range; SPT = S. Paratyphi naïve; SPT-SPT = Homologous S. Paratyphi re-challenge. ST-SPT = Heterologous S. Paratyphi re-challenge. ED = Met criteria for enteric fever diagnosis. nED = Did not meet criteria for enteric fever diagnosis.
(PDF)

S12 Fig Antibody response to *Salmonella* Typhi (a) and Paratyphi *A* (b) antigens following challenge/re-challenge with *Salmonella* Typhi (a) and Paratyphi (b). Grouped according to antigen and outcome (ED = Met criteria for enteric fever diagnosis. nED = Did not meet criteria for enteric fever diagnosis). Coloured lines connect median values for each timepoint. Grey lines connect paired samples across timepoints; Box plots display median, interquartile range; p = Mann-Whitney test.
(PDF)

S1 Data PATCH data. (XLSX)

S1 Methods Supplementary Methods. (DOCX)

S1 Protocol (PDF)
