# Supplementary material for: Homologous and heterologous re-challenge with Salmonella Typhi and Salmonella Paratyphi A in a randomised controlled human infection model
Source: PLoS Negl Trop Dis. 2020 Oct 20;14(10):e0008783. doi: 10.1371/journal.pntd.0008783 (PMC7598925; doi:10.1371/journal.pntd.0008783)
Supplement: S1 Table — (DOCX) [file pntd.0008783.s002.docx]

S1 Table - Serious adverse events OVG2014/01 study.

| **Challenge Group** | **Criteria** | **Event** | **Challenge related** | **Comment** |
| --- | --- | --- | --- | --- |
| ***S.* Typhi (Naïve)** | In-patient hospitalisation or prolongation | Collapse | No | Episode of collapse with loss of consciousness 12 months after challenge. Reported left arm and left leg weakness 24 hours’ duration. Diagnosed as possible generalised seizure following neurology review. Instructed not to drive for 6/12. No medication prescribed, and no subsequent events. |
| ***S.* Paratyphi (Naïve)** | In-patient hospitalisation or prolongation | Hospital admission for IV fluids | Yes | Nausea and vomiting, unresponsive to oral anti-emetics. Tachycardia. Admitted overnight for IV fluids and anti-emetics. Treatment switched to IV Ceftriaxone. Discharged after <24 hours and completed course of oral ciprofloxacin. |
| **NA** | Medically important event | Renal Colic | No | Right loin pain with evidence of renal calculus on CT KUB. Diagnosed after enrolment but prior to challenge. Withdrawn from study and not challenged. |
| ***S.* Paratyphi (Heterologous re-challenge ST-SPT)** | Medically important event | Raised ALT | Yes | Alanine aminotransferase elevated to 898 IU/L 5 days after diagnosis. Ascribed to paratyphoid fever plus possible adverse drug reaction (azithromycin + paracetamol). Antibiotic switched from azithromycin to ciprofloxacin. Paracetamol withheld. Resolved. |
