# Supplementary material for: Homologous and heterologous re-challenge with Salmonella Typhi and Salmonella Paratyphi A in a randomised controlled human infection model
Source: PLoS Negl Trop Dis. 2020 Oct 20;14(10):e0008783. doi: 10.1371/journal.pntd.0008783 (PMC7598925; doi:10.1371/journal.pntd.0008783)
Supplement: S2 Table — Hypokalaemia was observed in four participants, which was attributed to pseudohypokalaemia resulting from delayed sample transport over periods of high ambient temperature. (DOCX) [file pntd.0008783.s003.docx]

S2 Table - Participants meeting pre-specified criteria for severe enteric fever. Hypokalaemia was observed in four participants, which was attributed to pseudohypokalaemia resulting from delayed sample transport over periods of high ambient temperature[1]

| **Challenge Group** | **Criteria** | **Event** | **Challenge related** | **Comment** |
| --- | --- | --- | --- | --- |
| ***S.* Typhi Naïve** | Grade 4 laboratory abnormality | Hypokalaemia | No | K+ 2.9 mmol/l  At time of typhoid diagnosis |
| ***S.* Typhi Naïve** | Grade 4 laboratory abnormality | Hypokalaemia | No | K+ 2.8 mmol/l  24 hours after typhoid diagnosis |
| ***S.* Paratyphi (Heterologous re-challenge ST-SPT)** | Grade 4 laboratory abnormality | Hypokalaemia | No | K+ 2.9 mmol/l |
| ***S.* Paratyphi (Heterologous re-challenge ST-SPT)** | Grade 4 laboratory abnormality | Hypokalaemia | No | K+ 2.8 mmol/l |
| ***S.* Typhi (Heterologous re-challenge SPT-ST)** | Grade 4 laboratory abnormality | Elevated CRP | Yes | CRP 231mmol/l 48 hours after typhoid diagnosis |
