# Supplementary material for: Homologous and heterologous re-challenge with Salmonella Typhi and Salmonella Paratyphi A in a randomised controlled human infection model
Source: PLoS Negl Trop Dis. 2020 Oct 20;14(10):e0008783. doi: 10.1371/journal.pntd.0008783 (PMC7598925; doi:10.1371/journal.pntd.0008783)
Supplement: S3 Table — Participants from the OVG2014/01 (n = 113) and OVG2014/08 (n = 103) studies were eligible for re-challenge after 12 months had elapsed from their primary challenge. As these studies were running contemporaneously, only a proportion of participants from these studies were eligible for re-challenge by the end of recruitment. (DOCX) [file pntd.0008783.s004.docx]

**S3 Table - Number (%) of participants enrolled for re-challenge from previous challenge studies. Participants from the OVG2014/01 (n=113) and OVG2014/08 (n=103) studies were eligible for re-challenge after 12 months had elapsed from their primary challenge. As these studies were running contemporaneously, only a proportion of participants from these studies were eligible for re-challenge by the end of recruitment**

| Study | Primary Challenge | Number Eligible  for Re-Challenge | Number (%) Enrolled  for Re-Challenge |
| --- | --- | --- | --- |
| OVG2009/10 (T1) [1] | *S*. Typhi Quailes strain | 40 | 14 (35%) |
| OVG2011/02 (T2)  NCT01405521 [2] | *S.* Typhi Quailes strain | 94 | 15 (16%) |
| OVG2013/07 (P1)  NCT02100397 [3] | *S.* Paratyphi A NVGH308 strain | 40 | 22 (55%) |
| OVG2014/08 (VAST)  NCT02324751 [4] | *S.* Typhi Quailes strain | - | 23 |
| OVG2014/01 (PATCH)  NCT02192008 | *S.* Paratyphi A NVGH308 strain/  *S.* Typhi Quailes strain | - | 3 |

References

1. Waddington CS, Darton TC, Jones C, Haworth K, Peters A, John T, et al. An outpatient, ambulant-design, controlled human infection model using escalating doses of Salmonella Typhi challenge delivered in sodium bicarbonate solution. Clin Infect Dis. 2014;58: 1230–40. doi:10.1093/cid/ciu078

2. Darton TC, Jones C, Blohmke CJ, Waddington CS, Zhou L, Peters A, et al. Using a Human Challenge Model of Infection to Measure Vaccine Efficacy: A Randomised, Controlled Trial Comparing the Typhoid Vaccines M01ZH09 with Placebo and Ty21a. PLoS Negl Trop Dis. 2016;10: e0004926. doi:10.1371/journal.pntd.0004926

3. Dobinson HC, Gibani MM, Jones C, Thomaides-Brears HB, Voysey M, Darton TC, et al. Evaluation of the clinical and microbiological response to salmonella paratyphi a infection in the first paratyphoid human challenge model. Clin Infect Dis. 2017;64. doi:10.1093/cid/cix042

4. Jin C, Gibani MM, Moore M, Juel HB, Jones E, Meiring J, et al. Efficacy and immunogenicity of a Vi-tetanus toxoid conjugate vaccine in the prevention of typhoid fever using a controlled human infection model of Salmonella Typhi: a randomised controlled, phase 2b trial. Lancet. 2017;390: 2472–2480.
