# Supplementary material for: Homologous and heterologous re-challenge with Salmonella Typhi and Salmonella Paratyphi A in a randomised controlled human infection model
Source: PLoS Negl Trop Dis. 2020 Oct 20;14(10):e0008783. doi: 10.1371/journal.pntd.0008783 (PMC7598925; doi:10.1371/journal.pntd.0008783)
Supplement: S4 Table — ST = S. Typhi. SPT = S. Paratyphi A. p = Fishers exact test. (DOCX) [file pntd.0008783.s005.docx]

S4 Table - Attack rates according to alternative diagnostic criteria. ST = *S*. Typhi. SPT = *S*. Paratyphi A. p = Fishers exact test.

|  | Challenge Group | | | | | |
| --- | --- | --- | --- | --- | --- | --- |
|  | S. Typhi Challenge | | | S. Paratyphi Challenge | | |
|  | ST Naïve | ST-ST Re-Challenge | SPT-ST Re-Challenge | SPT Naïve | SPT-SPT Re-Challenge | ST-SPT Re-Challenge |
| Composite Criteria, n (% attack rate) | 12/19  (63%) | 12/27  (44%) | 7/10  (70%) | 10/18  (56%) | 3/12  (25%) | 13/26  (50%) |
| RR (95% CI)  Compared with naïve controls | Ref. | 0.7  (0.40-1.23) | 1.10  (0.59-1.87) | Ref. | 0.15  (0.15-1.14) | 0.90  (0.51-1.58) |
| p | Ref. | 0.24 | 0.99 | Ref. | 0.14 | 0.76 |
| Fever ≥38˚C (any duration) | 9/19  (47%) | 9/27  (33%) | 3/10  (30%) | 6/18  (33%) | 1/12  (8%) | 8/26  (31%) |
| RR (95% CI)  Compared with naïve controls | Ref. | 0.70  (0.35-1.45) | 0.63  (0.21-1.60) | Ref. | 0.25  (0.04 – 1.29) | 1.1  (0.45-2.58) |
| p | Ref. | 0.37 | 0.44 | Ref. | 0.19 | 0.99 |
| Fever ≥38·0˚C (any duration) + *S.* Typhi bacteraemia | 9/19  (47%) | 7/27  (26%) | 3/10  (30%) | 5/18  (27%) | 1/12  (8%) | 8/26  (31%) |
| RR (95% CI)  Compared with naïve controls | Ref. | 0.54  (0.25-120) | 0.63  (0.21-1.60) | Ref. | 0.3  (0.05-1.61) | 0.90  (0.35 -2.31) |
| p | Ref. | 0.20 | 0.44 | Ref. | 0.35 | 0.99 |
| Fever ≥38·0˚C (any duration) with subsequent bacteraemia | 0/19  (0%) | 4/27  (15%) | 0/10  (0%) | 2/18  (11%) | 1/12  (8%) | 2/26  (8%) |
| RR (95% CI)  Compared with naïve controls | Ref. | - | - | Ref. | 0.75  (0.10 – 5.14) | 0.69  (0.11 – 4.4) |
| p | Ref. | - | - | Ref. | 0.99 | 0.99 |
| *S*. Typhi bacteraemia OR stool shedding | 14/19  (74%) | 20/27  (74%) | 9/10  (90%) | 14/18  (77%) | 5/12  (42%) | 15/26  (58%) |
| RR (95% CI)  Compared with naïve controls | Ref. | 1  (0.71-1.51) | 1.22  (0.77 – 1.80) | Ref. | 0.75  (0.10 – 5.14) | 0.74  (0.49 – 1.12) |
| p | Ref. | 0.99 | 0.63 | Ref. | 0.06 | 0.20 |
| *S*. Typhi bacteraemia AND stool shedding | 7/19 (37%) | 6/27  (22%) | 7/10  (70%) | 4/18 (22%) | 2/12  (17%) | 7/26  (27%) |
| RR (95% CI)  Compared with naïve controls | Ref. | 0.6  (0.24-1.49) | 1.9  (0.9 – 3.9) | Ref. | 0.75  (0.18-2.96) | 1.21  (0.41-3.53) |
| p | Ref. | 0.33 | 0.12 | Ref. | 0.99 | 0.99 |
| Any Shedding | 9/19 (47%) | 15/27 (56%) | 9/10  (90%) | 9/18 (50%) | 4/12  (33%) | 9/26  (35%) |
| RR (95% CI)  Compared with naïve controls | Ref. | 1.17  (0.67-2.19) | 1.9  (1.10 – 3.37) | Ref. | 0.67  (0.25-1.54) | 0.69  (0.34 – 1.40) |
| p | Ref. | 0.76 | 0.043 | Ref. | 0.47 | 0.36 |
