# Supplementary material for: Homologous and heterologous re-challenge with Salmonella Typhi and Salmonella Paratyphi A in a randomised controlled human infection model
Source: PLoS Negl Trop Dis. 2020 Oct 20;14(10):e0008783. doi: 10.1371/journal.pntd.0008783 (PMC7598925; doi:10.1371/journal.pntd.0008783)
Supplement: S5 Table — (DOCX) [file pntd.0008783.s006.docx]

S5 Table - *Salmonella* Typhi and Paratyphi controlled human infection studies conducted in Oxford 2011- 2017.

| Study | Challenge agent | Study type | Vaccine | Description | Ref |
| --- | --- | --- | --- | --- | --- |
| OVG2009/10  (T1) | *S*. Typhi Quailes strain | Observational | - | Dose finding study  Low dose: 1-5 x 10^3^ CFU (n=20)  High dose: 1-5 x 10^4^ CFU (n=20) | [1] |
| OVG2011/02  NCT01405521  (T2) | *S*. Typhi Quailes strain | Vaccine RCT | M01ZH09 (n=31)  Ty21a (n=30)  Placebo (n=30) | Ty21a or M01ZH09 vaccines compared with control.  Challenge dose 1-5 x 10^4^ CFU | [2] |
| OVG2013/07  NCT02100397  (P1) | *S.* Paratyphi A NVGH308 strain | Observational | - | Dose finding study  High dose: 1-5 x 10^3^ CFU (n=20)  Low dose: 0.5-1 x 10^3^ CFU (n=20) | [3] |
| OVG2014/08  NCT02324751 | *S.* Typhi Quailes strain | Vaccine RCT | Vi-Polysaccharide (n=35)  Vi-TT conjugate (n=37)  Placebo (n=31) | Vi-PS (Typhim Vi ®, Sanofi Pasteur) or Vi-TT (TypbarTCV ®, Bharat Biotech) vaccines compared with control.  Challenge dose 1-5 x 10^4^ CFU | [4] |
| OVG2014/01  NCT02192008 | *S.* Paratyphi A NVGH308 strain  *S.* Typhi Quailes strain | RCT | - | Naïve challenge (S. Typhi and S. Paratyphi) vs Re-challenge (homologous and heterologous)  S. Typhi challenge dose 1-5 x 10^4^ CFU  S. Paratyphi challenge dose 1-5 x 10^3^ CFU | [5] |
| OVG2016/03  NCT03067961 | *S.* Typhi Quailes strain/  *S*. Typhi SB6000 (Typhoid-toxin deficient) | RCT | - | Wild Type S. Typhi Quailes strain (n=20)  SB6000 Typhoid toxin negative strain (n=20)  Challenge dose 1-5 x 10^4^ CFU | [6] |

References

1. Waddington CS, Darton TC, Jones C, Haworth K, Peters A, John T, et al. An outpatient, ambulant-design, controlled human infection model using escalating doses of Salmonella Typhi challenge delivered in sodium bicarbonate solution. Clin Infect Dis. 2014;58: 1230–40. doi:10.1093/cid/ciu078

2. Darton TC, Jones C, Blohmke CJ, Waddington CS, Zhou L, Peters A, et al. Using a Human Challenge Model of Infection to Measure Vaccine Efficacy: A Randomised, Controlled Trial Comparing the Typhoid Vaccines M01ZH09 with Placebo and Ty21a. PLoS Negl Trop Dis. 2016;10: e0004926. doi:10.1371/journal.pntd.0004926

3. Dobinson HC, Gibani MM, Jones C, Thomaides-Brears HB, Voysey M, Darton TC, et al. Evaluation of the clinical and microbiological response to salmonella paratyphi a infection in the first paratyphoid human challenge model. Clin Infect Dis. 2017;64. doi:10.1093/cid/cix042

4. Jin C, Gibani MM, Moore M, Juel HB, Jones E, Meiring J, et al. Efficacy and immunogenicity of a Vi-tetanus toxoid conjugate vaccine in the prevention of typhoid fever using a controlled human infection model of Salmonella Typhi: a randomised controlled, phase 2b trial. Lancet. 2017;390: 2472–2480.

5. Gibani M, Jin C, Thomaides-Brears H, Shrestha S, Precaido-Llanes L, Napolitani G, et al. Investigating Systemic Immunity to Typhoid and Paratyphoid Fever: Characterising the Response to Re-challenge in a Controlled Human Infection Model. Open Forum Infect Dis. Oxford University Press; 2017;4: S227–S228. doi:10.1093/ofid/ofx163.474

6. Gibani MM, Jones E, Barton A, Jin C, Meek J, Camara S, et al. Investigation of the role of typhoid toxin in acute typhoid fever in a human challenge model. Nat Med. Nature Publishing Group; 2019;25: 1082–1088. doi:10.1038/s41591-019-0505-4
