# Supplementary material for: Homologous and heterologous re-challenge with Salmonella Typhi and Salmonella Paratyphi A in a randomised controlled human infection model
Source: PLoS Negl Trop Dis. 2020 Oct 20;14(10):e0008783. doi: 10.1371/journal.pntd.0008783 (PMC7598925; doi:10.1371/journal.pntd.0008783)
Supplement: S1 Fig — Participants were recruited into one of three study groups defined a priori according to prior challenge status. (PDF) [file pntd.0008783.s007.pdf]

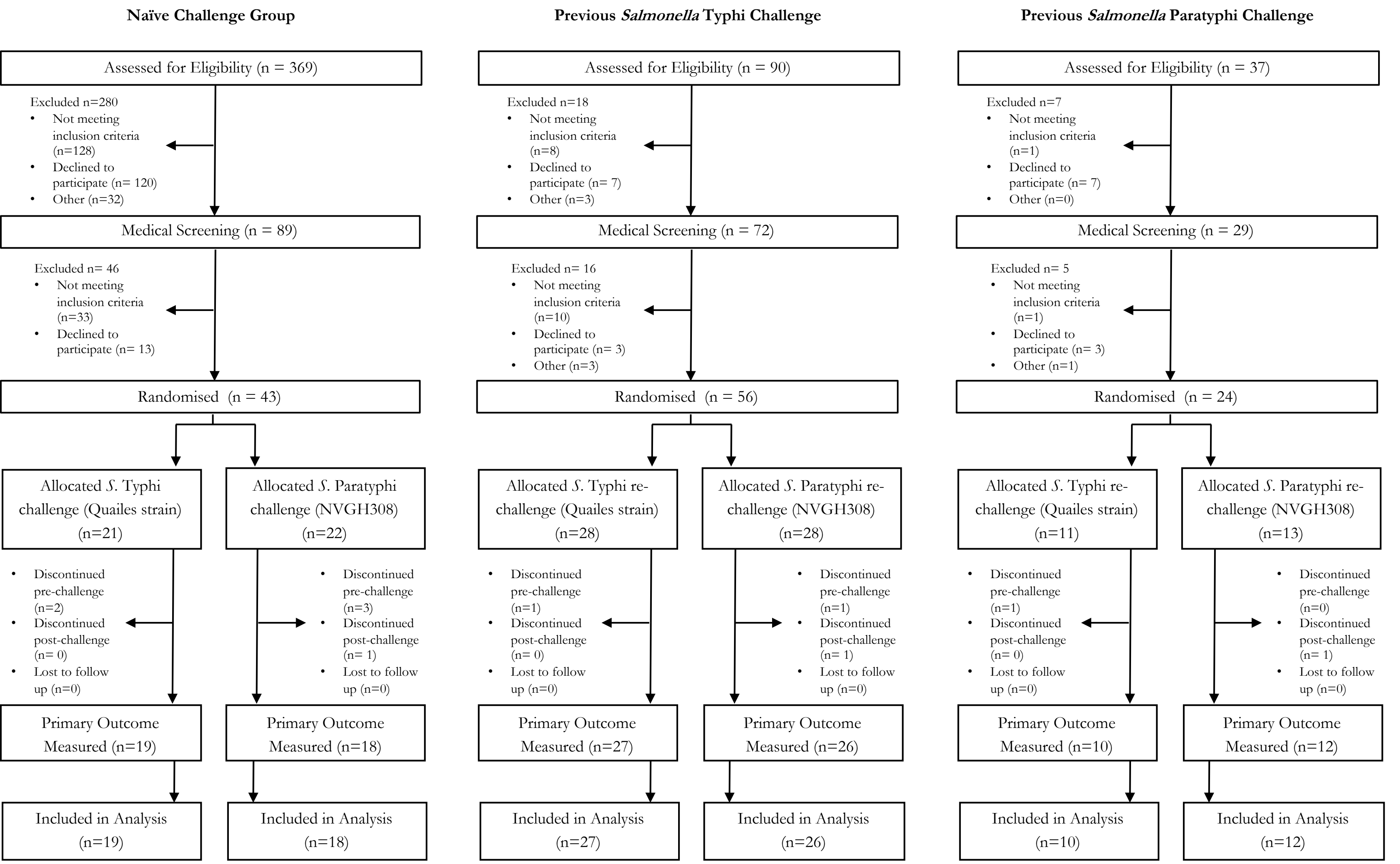
Naïve Challenge Group
Previous *Salmonella* Typhi Challenge
Previous *Salmonella* Paratyphi Challenge
Assessed for Eligibility (n = 369)
Excluded n=280
• Not meeting inclusion criteria (n=128)
• Declined to participate (n= 120)
• Other (n=32)
Medical Screening (n = 89)
Excluded n= 46
• Not meeting inclusion criteria (n=33)
• Declined to participate (n= 13)
Randomised (n = 43)
Allocated *S*. Typhi challenge (Quailes strain) (n=21)
Allocated *S*. Paratyphi challenge (NVGH308) (n=22)
• Discontinued pre-challenge (n=2)
• Discontinued post-challenge (n= 0)
• Lost to follow up (n=0)
• Discontinued pre-challenge (n=3)
• Discontinued post-challenge (n= 1)
• Lost to follow up (n=0)
Primary Outcome Measured (n=19)
Primary Outcome Measured (n=18)
Included in Analysis (n=19)
Included in Analysis (n=18)
Assessed for Eligibility (n = 90)
Excluded n=18
• Not meeting inclusion criteria (n=8)
• Declined to participate (n= 7)
• Other (n=3)
Medical Screening (n = 72)
Excluded n= 16
• Not meeting inclusion criteria (n=10)
• Declined to participate (n= 3)
• Other (n=3)
Randomised (n = 56)
Allocated *S*. Typhi re-challenge (Quailes strain) (n=28)
Allocated *S*. Paratyphi re-challenge (NVGH308) (n=28)
• Discontinued pre-challenge (n=1)
• Discontinued post-challenge (n= 0)
• Lost to follow up (n=0)
• Discontinued pre-challenge (n=1)
• Discontinued post-challenge (n= 1)
• Lost to follow up (n=0)
Primary Outcome Measured (n=27)
Primary Outcome Measured (n=26)
Included in Analysis (n=27)
Included in Analysis (n=26)
Assessed for Eligibility (n = 37)
Excluded n=7
• Not meeting inclusion criteria (n=1)
• Declined to participate (n= 7)
• Other (n=0)
Medical Screening (n = 29)
Excluded n= 5
• Not meeting inclusion criteria (n=1)
• Declined to participate (n= 3)
• Other (n=1)
Randomised (n = 24)
Allocated *S*. Typhi re-challenge (Quailes strain) (n=11)
Allocated *S*. Paratyphi re-challenge (NVGH308) (n=13)
• Discontinued pre-challenge (n=1)
• Discontinued post-challenge (n= 0)
• Lost to follow up (n=0)
• Discontinued pre-challenge (n=0)
• Discontinued post-challenge (n= 1)
• Lost to follow up (n=0)
Primary Outcome Measured (n=10)
Primary Outcome Measured (n=12)
Included in Analysis (n=10)
Included in Analysis (n=12)
