# Supplementary figures and images for: Homologous and heterologous re-challenge with Salmonella Typhi and Salmonella Paratyphi A in a randomised controlled human infection model
Source: PLoS Negl Trop Dis. 2020 Oct 20;14(10):e0008783. doi: 10.1371/journal.pntd.0008783 (PMC7598925; doi:10.1371/journal.pntd.0008783)

# Re-Challenge Interval - Primary Challenge

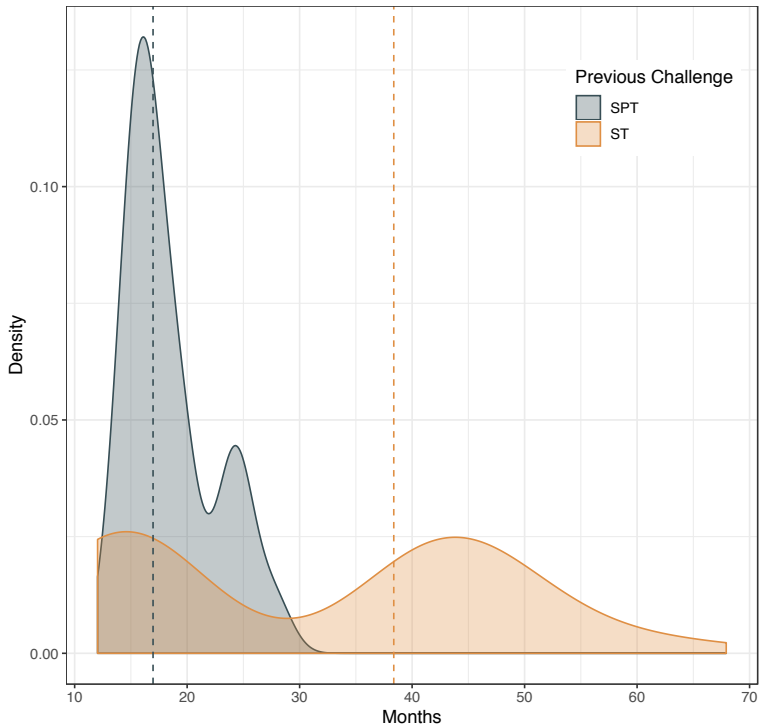

Supplement: S2 Fig — Density plot according to previous challenge agent allocation. Vertical lines represent median re-challenge interval for participants previously challenged with S. Typhi (orange) and S. Paratyphi (grey) (PDF) [file pntd.0008783.s008.pdf]

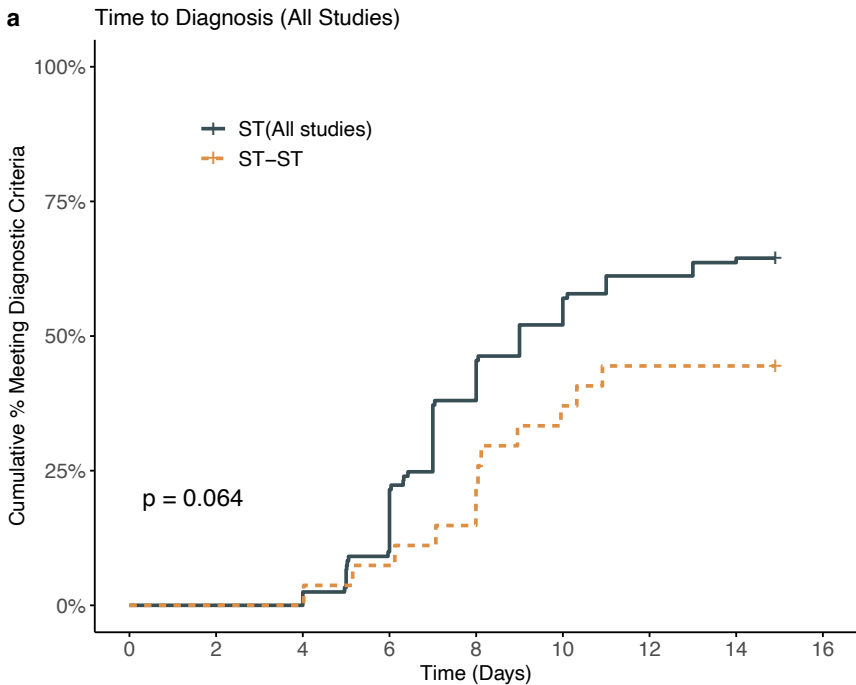

|                  |     |     |     |     |    |    |    |    |    |
|------------------|-----|-----|-----|-----|----|----|----|----|----|
| ST (All studies) | 121 | 121 | 121 | 109 | 75 | 58 | 47 | 44 | 0  |
| ST-ST            | 27  | 27  | 27  | 25  | 21 | 17 | 15 | 15 | 0  |
|                  | 0   | 2   | 4   | 6   | 8  | 10 | 12 | 14 | 16 |

Time (Days)

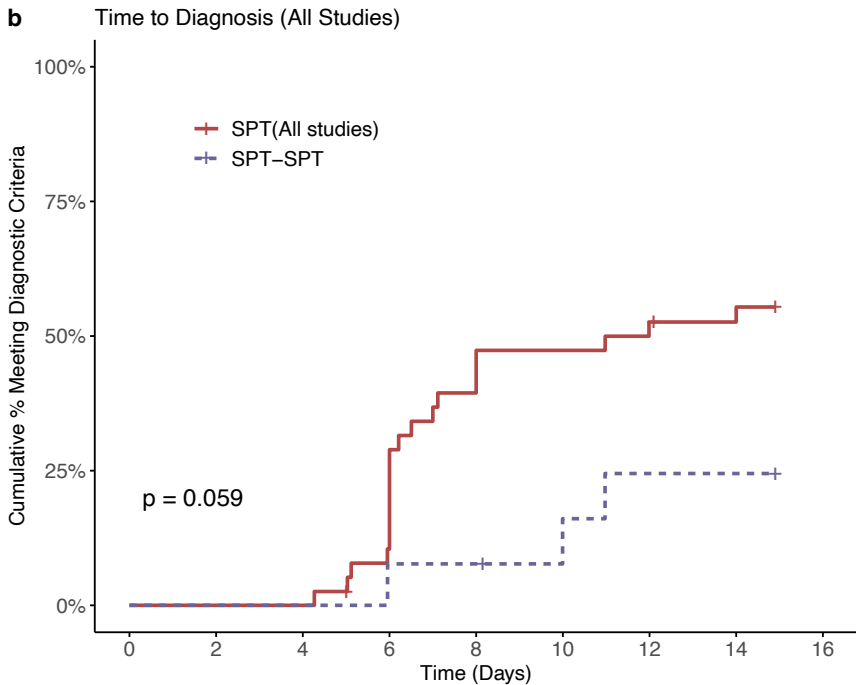

|                   |    |    |    |    |    |    |    |    |    |
|-------------------|----|----|----|----|----|----|----|----|----|
| SPT (All studies) | 39 | 39 | 39 | 34 | 23 | 20 | 18 | 17 | 0  |
| SPT-SPT           | 13 | 13 | 13 | 12 | 12 | 10 | 9  | 9  | 0  |
|                   | 0  | 2  | 4  | 6  | 8  | 10 | 12 | 14 | 16 |

Time (Days)

Supplement: S4 Fig — Cumulative incidence of typhoid (i) and paratyphoid A (ii) fever after challenge in naïve (ST& SPT) and homologous re-challenge (ST-ST & SPT-SPT) groups. Time to composite diagnostic endpoint, measured from challenge agent ingestion to development of first fever ≥38°C or first positive blood culture sampling. Non-diagnosed participants censored at day 14 hours. P value from log-rank test comparing ST = S. Typhi naïve challenge. ST-ST = Homologous Re-Challenge with S. Typhi. SPT = S. Paratyphi naïve challenge. SPT-SPT = Homologous Re-Challenge with S. Paratyphi. (PDF) [file pntd.0008783.s010.pdf]

# A

## S. Typhi Challenge

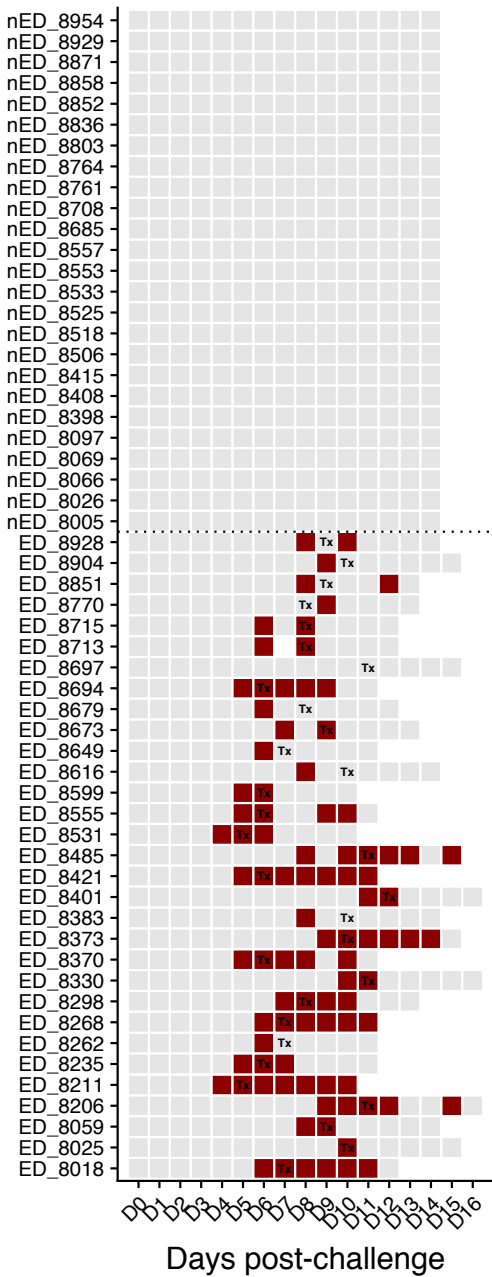

E

## S. Paratyphi Challenge

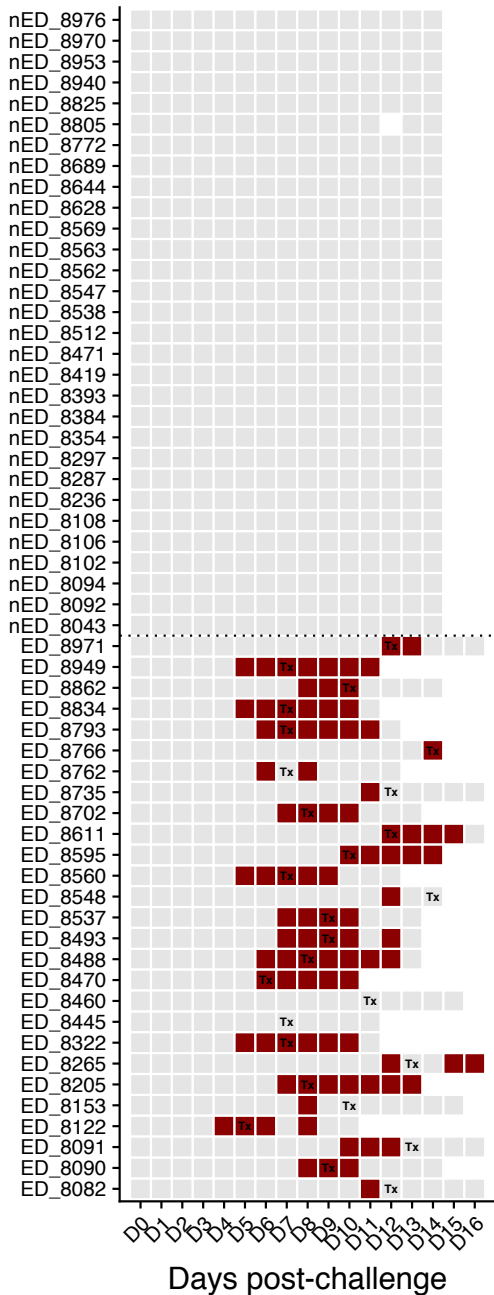

Supplement: S6 Fig — Pattern of bacteraemia following S. Typhi (A) and S. Paratyphi A (B) challenge. Each row corresponds to an individual participant. Grey squares = Negative sample, Red squares = Positive blood culture, White squares = No sample collected. Tx = Day of treatment initiation. Participants above the dotted line did not meet the composite criteria for typhoid or paratyphoid diagnosis. (PDF) [file pntd.0008783.s012.pdf]

**A****S. Typhi Challenge**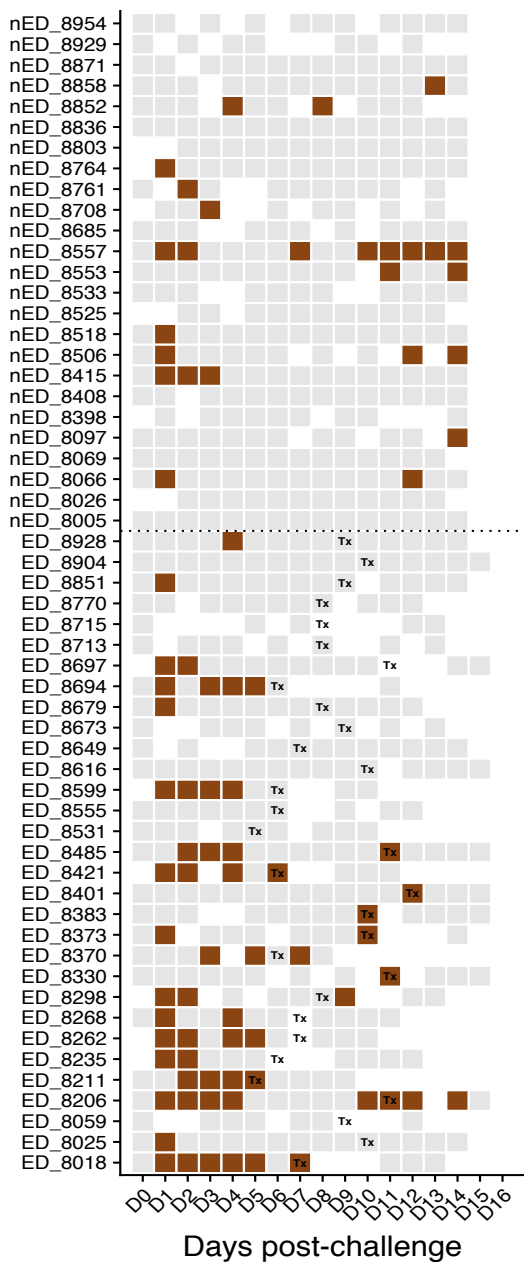**B****S. Paratyphi Challenge**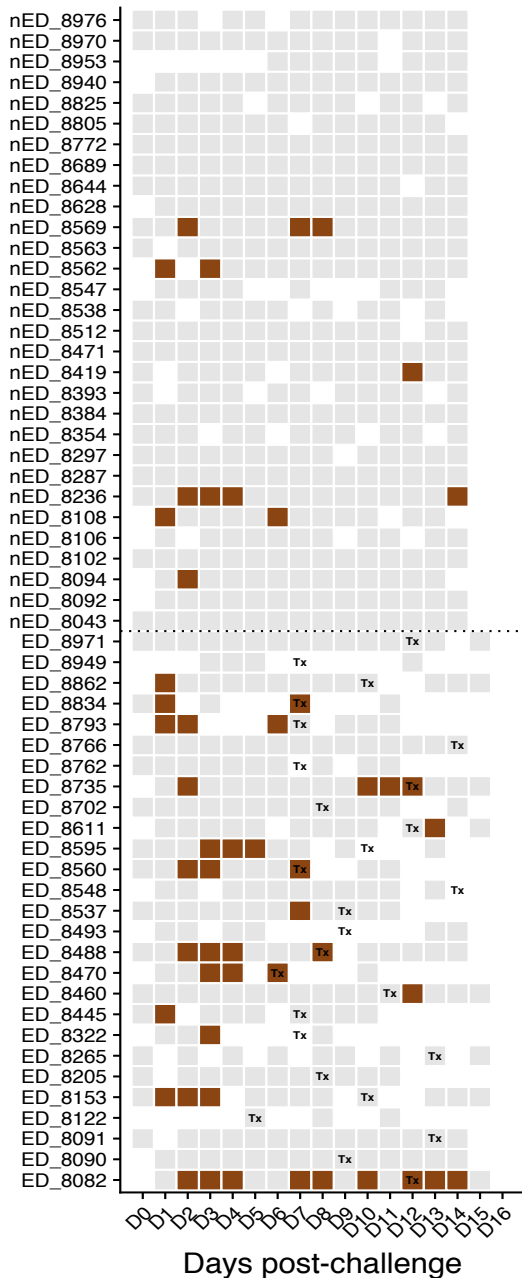

Supplement: S7 Fig — Each row corresponds to an individual participant. Grey squares = Negative sample, Brown squares = Positive stool culture, White squares = No sample collected. Tx = Day of treatment initiation. Participants above the dotted line did not meet the composite criteria for typhoid diagnosis. (PDF) [file pntd.0008783.s013.pdf]

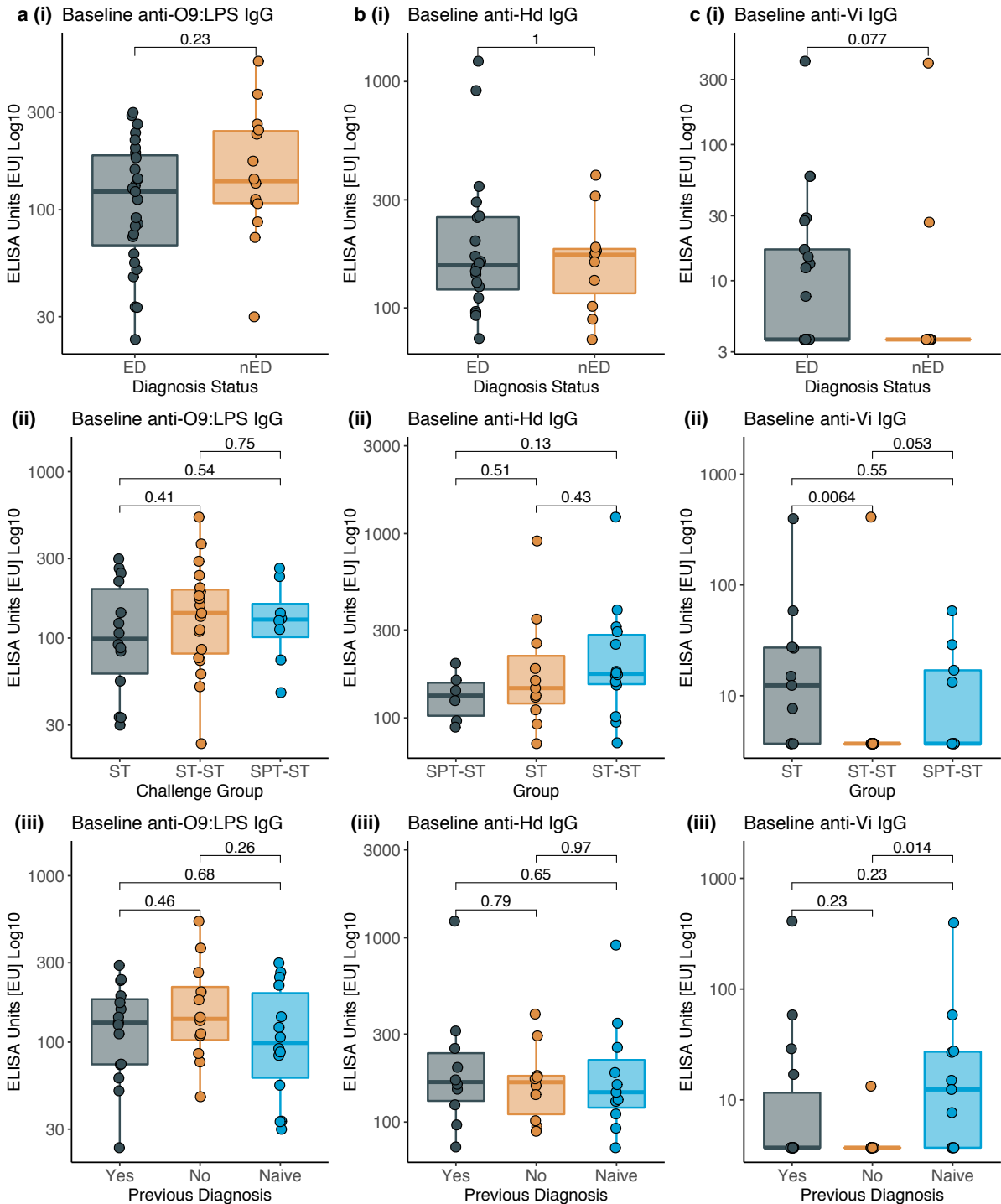

Supplement: S10 Fig — Baseline (Day 0) serum anti-O9:LPS (a) anti-Hd (b) and anti-Vi (c) IgG in participants challenged with S. Typhi, grouped according to (i) outcome of challenge (ii) challenge group and (iii) outcome of previous challenge. p = Mann-Whitney U test two sided; Box plots display median, interquartile range; ST = S. Typhi naïve; ST-ST = Homologous S. Typhi re-challenge. SPT-ST = Heterologous S. Typhi re-challenge. ED = Met criteria for enteric fever diagnosis. nED = Did not meet criteria for enteric fever diagnosis. (PDF) [file pntd.0008783.s016.pdf]

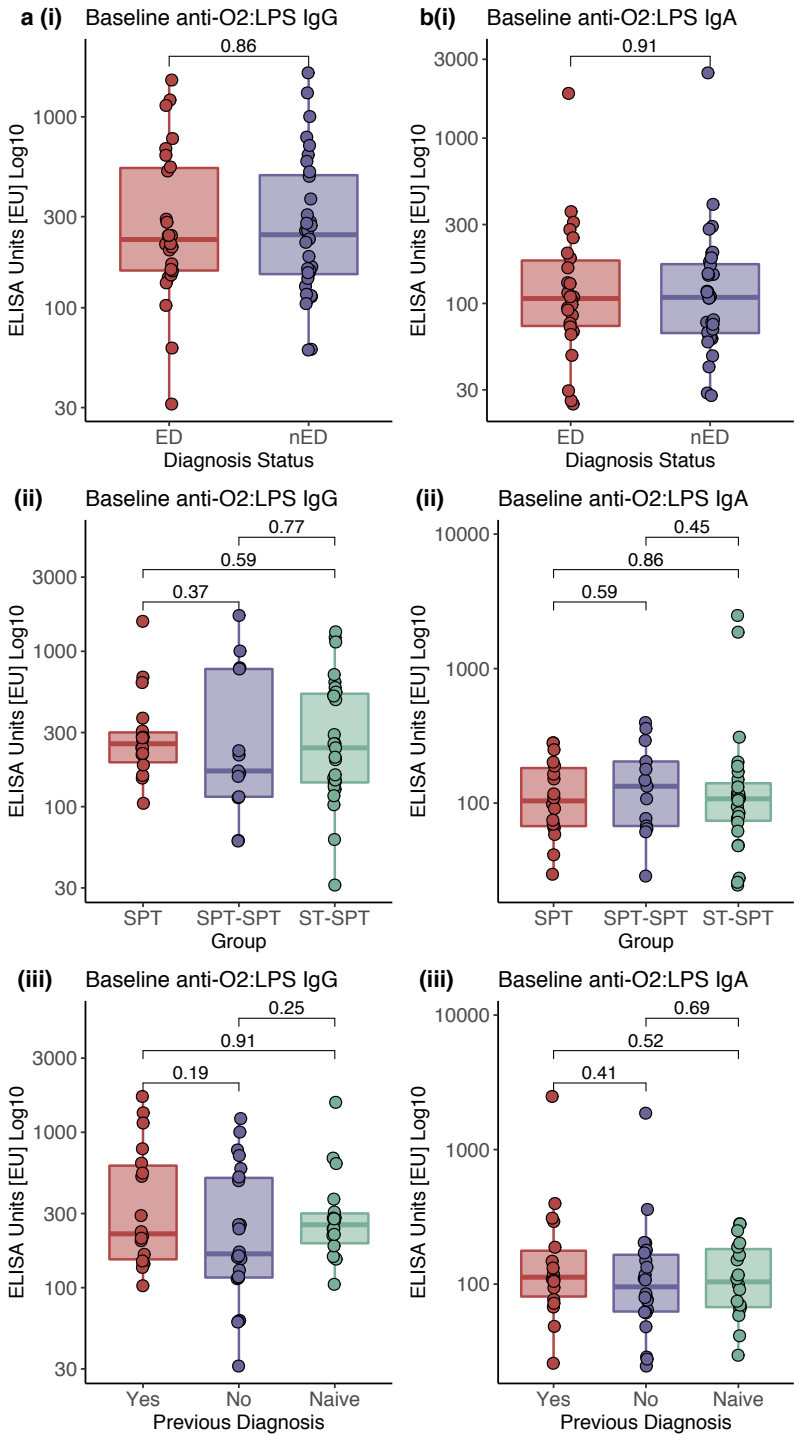

Supplement: S11 Fig — Baseline (Day 0) serum anti-O2:LPS IgG (a) and IgA in participants challenged with S. Paratyphi, grouped according to (i) outcome of challenge (ii) challenge group and (iii) outcome of previous challenge. p = Mann-Whitney U test two sided; Box plots display median, interquartile range; SPT = S. Paratyphi naïve; SPT-SPT = Homologous S. Paratyphi re-challenge. ST-SPT = Heterologous S. Paratyphi re-challenge. ED = Met criteria for enteric fever diagnosis. nED = Did not meet criteria for enteric fever diagnosis. (PDF) [file pntd.0008783.s017.pdf]

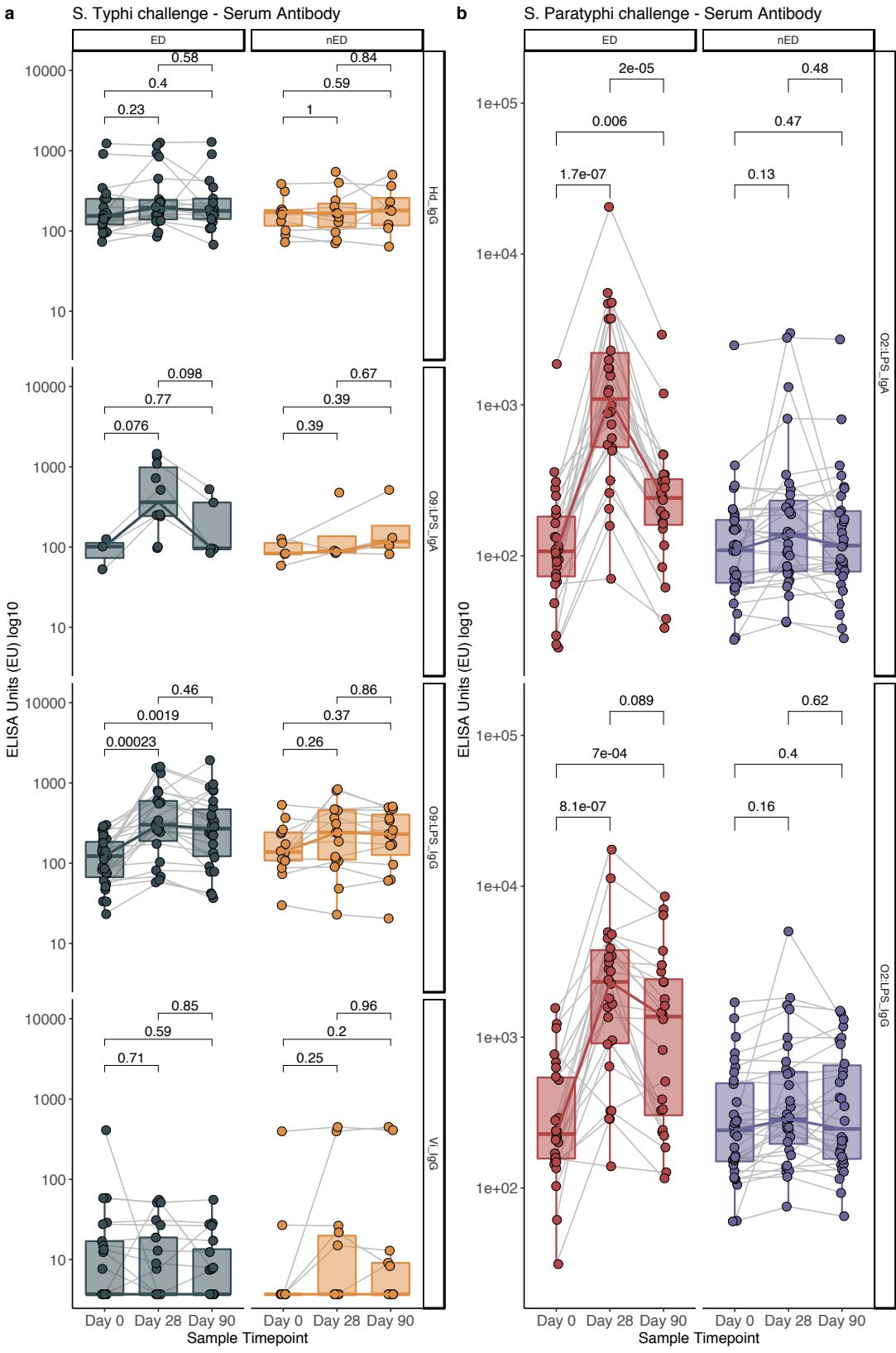

Supplement: S12 Fig — Antibody response to Salmonella Typhi (a) and Paratyphi A (b) antigens following challenge/re-challenge with Salmonella Typhi (a) and Paratyphi (b). Grouped according to antigen and outcome (ED = Met criteria for enteric fever diagnosis. nED = Did not meet criteria for enteric fever diagnosis). Coloured lines connect median values for each timepoint. Grey lines connect paired samples across timepoints; Box plots display median, interquartile range; p = Mann-Whitney test. (PDF) [file pntd.0008783.s018.pdf]
