# Supplementary material for: Homologous and heterologous re-challenge with Salmonella Typhi and Salmonella Paratyphi A in a randomised controlled human infection model
Source: PLoS Negl Trop Dis. 2020 Oct 20;14(10):e0008783. doi: 10.1371/journal.pntd.0008783 (PMC7598925; doi:10.1371/journal.pntd.0008783)
Supplement: S3 Fig — Forest plot illustrating attack rates in naive cohorts of S. Typhi (top) and S. Paratyphi (bottom) challenge studies. Heterogeneity I-squared = 0, test of homogeneity (Q statistic) gives p value for 0.78 (no evidence the proportions vary). (PDF) [file pntd.0008783.s009.pdf]

Attack Rate in Naive/Control Participants Challenged with wild-type S. Typhi or Paratyphi - Combined Analysis Across Studies

Study	Reference	No. of Participants [% Total]	Attack rate [95% CI]	Attack rate
**S. Typhi Challenge**				
**All naïve/control participants challenged with wild-type Quailes strain 1-5 x 10e4 CFU**				
OVG2009/10 (T1)	Waddington et al 2014 [1]	20 (17)	0.65(0.41-0.85)	13/20 (65%)
OVG2011/02 (T2)	Darton et al 2016 [2]	30 (25)	0.67(0.47-0.83)	20/30 (67%)
OVG2014/08 (VAST)	Jin et al 2017 [3]	31 (26)	0.77(0.59-0.9)	24/31 (77%)
OVG2014/01 (PATCH)	-	19 (16)	0.63(0.38-0.84)	12/19 (63%)
OVG2016/03 (TYGER)	Gibani et al 2019 [4]	21 (17)	0.71(0.48-0.89)	15/21 (62%)
**Overall**		**121 (100)**	**0.69(0.6-0.77)**	**84/121 (69%)**
**S. Paratyphi Challenge**				
**All naïve/control participants challenged with NVGH308 strain 1-5 x 10e3 CFU**				
OVG2013/07 (P1)	Dobinson et al 2017 [5]	20 (53)	0.6(0.36-0.81)	12/20 (60%)
OVG2014/01 (PATCH)	-	18 (47)	0.56(0.31-0.78)	10/18 (56%)
**Overall**		**38 (100)**	**0.58(0.41-0.74)**	**22/38 (58%)**

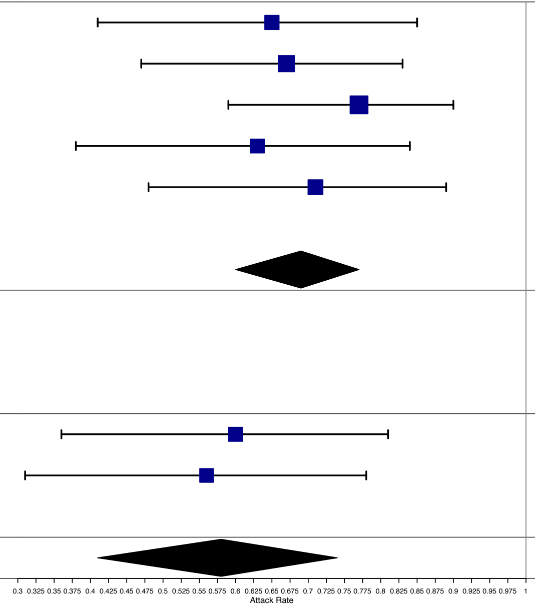

References

[1] Waddington CS, Darton TC, Jones C, Haworth K, Peters A, John T, et al. An outpatient, ambulant-design, controlled human infection model using escalating doses of Salmonella Typhi challenge delivered in sodium bicarbonate solution. Clin Infect Dis. 2014;58: 1230–40. doi:10.1093/cid/ciu078

[2] Darton TC, Jones C, Blohmke CJ, Waddington CS, Zhou L, Peters A, et al. Using a Human Challenge Model of Infection to Measure Vaccine Efficacy: A Randomised, Controlled Trial Comparing the Typhoid Vaccines M01ZH09 with Placebo and Ty21a. PLoS Negl Trop Dis. 2016;10: e0004926. doi:10.1371/journal.pntd.0004926

. [3] Jin C, Gibani MM, Moore M, Juel HB, Jones E, Meiring J, et al. Efficacy and immunogenicity of a Vi-tetanus toxoid conjugate vaccine in the prevention of typhoid fever using a controlled human infection model of Salmonella Typhi: a randomised controlled, phase 2b trial. Lancet. 2017;390: 2472–2480.

[4] Gibani MM, Jones E, Barton A, Jin C, Meek J, Camara S, et al. Investigation of the role of typhoid toxin in acute typhoid fever in a human challenge model. Nat Med. Nature Publishing Group; 2019;25: 1082–1088. doi:10.1038/s41591-019-0505-4

[5] Dobinson HC, Gibani MM, Jones C, Thomaides-Brears HB, Voysey M, Darton TC, et al. Evaluation of the clinical and microbiological response to salmonella paratyphi a infection in the first paratyphoid human challenge model. Clin Infect Dis. 2017;64. doi:10.1093/cid/cix042
