# Supplementary material for: Homologous and heterologous re-challenge with Salmonella Typhi and Salmonella Paratyphi A in a randomised controlled human infection model
Source: PLoS Negl Trop Dis. 2020 Oct 20;14(10):e0008783. doi: 10.1371/journal.pntd.0008783 (PMC7598925; doi:10.1371/journal.pntd.0008783)
Supplement: S5 Fig — Timepoints in a and c are normalised to the day of diagnosis (ED day = 0). Timepoints b and d in non-diagnosed participants represent day of sample collection. Box-and whisker plots represent median and interquartile range. Solid coloured lines link median value at each time point. Grey lines connect paired data points from the same individuals. Units: Haemoglobin = (g/dL); Haemoglobin change g/dL compared with Hb Day 0; Haematocrit(L/L); White cell count/Neutrophil count/Lymphocyte count/Eosinophil count/Monocyte count = cells x 109/L; Urea = mmol/L; Creatinine = mg/L; Na+/K+ = mEq/L; C-reactive protein = mmol/l; Bilirubin = umol/l; ALT–IU/l; ALP = U/L; Albumin = g/L. (PDF) [file pntd.0008783.s011.pdf]

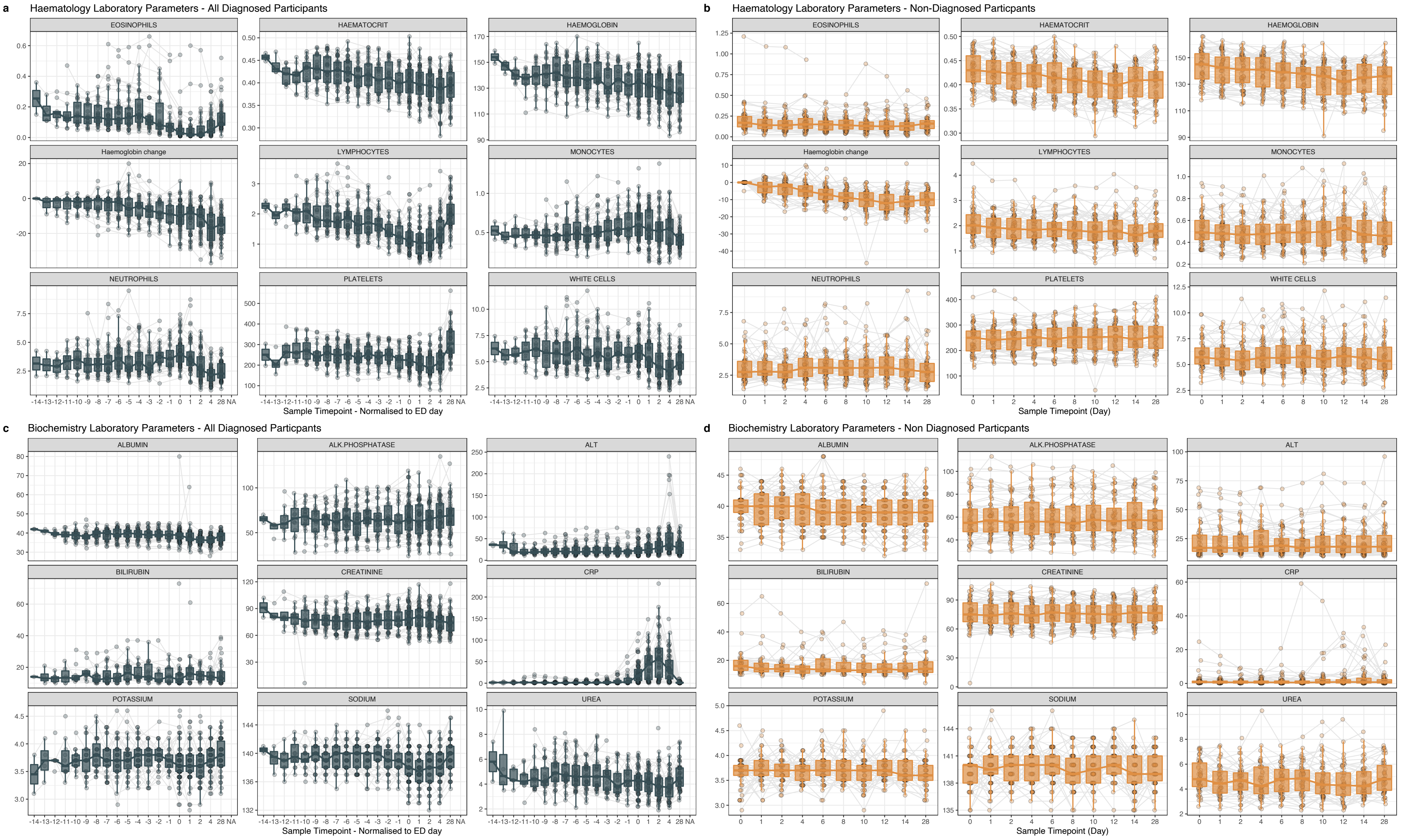
a
Haematology Laboratory Parameters - All Diagnosed Participants
EOSINOPHILS
HAEMATOCRIT
HAEMOGLOBIN
Haemoglobin change
LYMPHOCYTES
MONOCYTES
NEUTROPHILS
PLATELETS
WHITE CELLS
Sample Timepoint - Normalised to ED day
b
Haematology Laboratory Parameters - Non-Diagnosed Particpants
EOSINOPHILS
HAEMATOCRIT
HAEMOGLOBIN
Haemoglobin change
LYMPHOCYTES
MONOCYTES
NEUTROPHILS
PLATELETS
WHITE CELLS
Sample Timepoint (Day)
c
Biochemistry Laboratory Parameters - All Diagnosed Particpants
ALBUMIN
ALK.PHOSPHATASE
ALT
BILIRUBIN
CREATININE
CRP
POTASSIUM
SODIUM
UREA
Sample Timepoint - Normalised to ED day
d
Biochemistry Laboratory Parameters - Non Diagnosed Particpants
ALBUMIN
ALK.PHOSPHATASE
ALT
BILIRUBIN
CREATININE
CRP
POTASSIUM
SODIUM
UREA
Sample Timepoint (Day)
