# Supplementary material for: Homologous and heterologous re-challenge with Salmonella Typhi and Salmonella Paratyphi A in a randomised controlled human infection model
Source: PLoS Negl Trop Dis. 2020 Oct 20;14(10):e0008783. doi: 10.1371/journal.pntd.0008783 (PMC7598925; doi:10.1371/journal.pntd.0008783)

**S. Typhi & Paratyphi Re-Challenge - Relative Risk of Composite Diagnositic Endpoint by Subgroup (vs. Combined Naive Controls)**

| Subgroup | No. of Participants [%] | Re-Challenge Group % AR | Naive Group % AR | RR[95%CI] | P Value |
|---|---|---|---|---|---|
| **Overall S. Typhi-S. Typhi Re-challenge** | **27 (100)** | **12/27(44%)** | **12/19(63%)** | **0.7(0.41-1.21)** | **0.24** |
| Outcome of Primary Challenge | | | | | |
| Typhoid Challenge - Typhoid Diagnosis | 12 (44.4) | 8/12(67%) | 12/19(63%) | 1.06(0.62-1.79) | 0.99 |
| Typhoid Challenge - No previous Diagnosis | 15 (55.6) | 4/15(27%) | 12/19(63%) | 0.42(0.17-1.05) | 0.045 |
| Previous Vaccine & Typhoid Challenge | | | | | |
| Any Vaccine | 11 (41) | 5/11(45%) | 12/19(63%) | 0.72(0.35-1.5) | 0.45 |
| Previous Vi-PS | 4 (15) | 2/4(50%) | 12/19(63%) | 0.79(0.28-2.24) | 0.99 |
| Previous Vi-TT | 3 (11) | 0/3(0%) | 12/19(63%) | | 0.08 |
| Previous Ty21a | 3 (11) | 2/3(67%) | 12/19(63%) | 1.06(0.44-2.52) | 0.99 |
| Previous M01ZH09 | 1 (4) | 1/1(100%) | 12/19(63%) | | 0.99 |
| No Previous Vaccine | 16 (59) | 7/16(44%) | 12/19(63%) | 0.69(0.36-1.33) | 0.31 |
| **Overall S. Paratyphi-S. Typhi Re-challenge** | **10 (100)** | **7/10(70%)** | **12/19(63%)** | **1.1(0.65-1.89)** | **0.99** |
| Outcome of Primary Challenge | | | | | |
| Paratyphoid Challenge - Paratyphoid Diagnosis | 7 (70) | 6/7(86%) | 12/19(63%) | 1.35(0.86-2.15) | 0.37 |
| Paratyphoid Challenge - No previous Diagnosis | 3 (30) | 1/3(33%) | 12/19(63%) | 0.53(0.1-2.71) | 0.54 |
| **Overall S. Paratyphi-S.Paratyphi Re-Challenge** | **12 (100)** | **3/12(25%)** | **10/18 (56%)** | **0.45(0.16-1.3)** | **0.14** |
| Outcome of Primary Challenge | | | | | |
| Paratyphoid Challenge - Paratyphoid Diagnosis | 5 (42) | 1/5(20%) | 10/18 (56%) | 0.36(0.06-2.18) | 0.32 |
| Paratyphoid Challenge - No previous Diagnosis | 7 (58) | 2/7 (29%) | 10/18 (56%) | 0.51(0.15-1.78) | 0.38 |
| **Overall S. Typhi-S. Paratyphi Re-Challenge** | **26 (100)** | **13/26(50%)** | **10/18 (56%)** | **0.9(0.51-1.58)** | **0.77** |
| Outcome of Primary Challenge | | | | | |
| Typhoid Challenge - Typhoid Diagnosis | 13 (50) | 10/13 (77%) | 10/18 (56%) | 1.39(0.83-2.3) | 0.28 |
| Typhoid Challenge - No previous Diagnosis | 13 (50) | 3/13 (23%) | 10/18 (56%) | 0.42(0.14-1.22) | 0.14 |
| Previous Vaccine & Typhoid Challenge | | | | | |
| Any Vaccine | 15 (58) | 7/15 (47%) | 10/18 (56%) | 0.84(0.43-1.66) | 0.73 |
| Previous Vi-PS | 3 (12) | 1/3 (33%) | 10/18 (56%) | 0.6(0.11-3.13) | 0.59 |
| Previous Vi-TT | 8 (31) | 5/8 (63%) | 10/18 (56%) | 1.13(0.57-2.22) | 0.99 |
| Previous Ty21a | 1 (4) | 0/1 (0%) | 10/18 (56%) | | 0.47 |
| Previous M01ZH09 | 3 (12) | 1/3 (33%) | 10/18 (56%) | 0.6(0.11-3.13) | 0.99 |
| No Previous Vaccine | 11 (42) | 6/11(55%) | 10/18 (56%) | 0.98(0.5-1.94) | 0.99 |

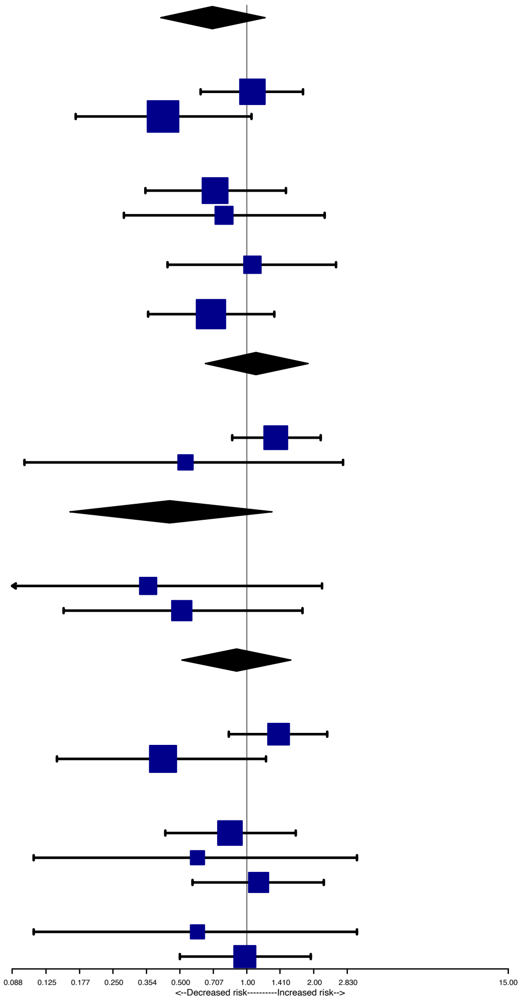

Supplement: S8 Fig — Forest plot comparing relative risk of typhoid or paratyphoid diagnosis following re-challenge compared with naïve controls from OVG2014/01 study. Box-plots represent relative risk and 95% confidence intervals scaled according to size of sub-group. Diamonds represent combined relative risk of diagnosis in each of the re-challenge cohorts. P = Fishers exact test. (PDF) [file pntd.0008783.s014.pdf]
