# Supplementary material for: Homologous and heterologous re-challenge with Salmonella Typhi and Salmonella Paratyphi A in a randomised controlled human infection model
Source: PLoS Negl Trop Dis. 2020 Oct 20;14(10):e0008783. doi: 10.1371/journal.pntd.0008783 (PMC7598925; doi:10.1371/journal.pntd.0008783)

S. Typhi & Paratyphi Re-Challenge - Relative Risk of Composite Diagnositic Endpoint by Subgroup (vs. Combined Naive Controls)

Subgroup	No. of Participants [%]	Re-Challenge Group % AR	Naive Group % AR	RR[95%CI]	P Value
**Overall S. Typhi-S. Typhi Re-challenge**	**27 (100)**	**12/27(44%)**	**84/121(69%)**	**0.64(0.41-0.99)**	**0.02**
Outcome of Primary Challenge					
Typhoid Challenge - Typhoid Diagnosis	12 (44.4)	8/12(67%)	84/121(69%)	0.96(0.63-1.45)	0.99
Typhoid Challenge - No previous Diagnosis	15 (55.6)	4/15(27%)	84/121(69%)	0.38(0.16-0.9)	0.002
Previous Vaccine & Typhoid Challenge					
Any Vaccine	11 (41)	5/11(45%)	84/121(69%)	0.65(0.34-1.26)	0.18
Previous Vi-PS	4 (15)	2/4(50%)	84/121(69%)	0.72(0.27-1.93)	0.64
Previous Vi-TT	3 (11)	0/3(0%)	84/121(69%)		0.03
Previous Ty21a	3 (11)	2/3(67%)	84/121(69%)	0.96(0.43-2.34)	0.99
Previous M01ZH09	1 (4)	1/1(100%)	84/121(69%)		0.99
No Previous Vaccine	16 (59)	7/16(44%)	84/121(69%)	0.63(0.36-1.11)	0.35
**Overall S. Paratyphi-S. Typhi Re-challenge**	**10 (100)**	**7/10(70%)**	**84/121(69%)**	**1.01(0.66-1.54)**	**0.99**
Outcome of Primary Challenge					
Paratyphoid Challenge - Paratyphoid Diagnosis	7 (70)	6/7(86%)	84/121(69%)	1.24(0.89-1.71)	0.67
Paratyphoid Challenge - No previous Diagnosis	3 (30)	1/3(33%)	84/121(69%)	0.48(0.19-2.39)	0.23
**Overall S. Paratyphi-S.Paratyphi Re-Challenge**	**12 (100)**	**3/12(25%)**	**22/38(58%)**	**0.43(0.16-1.19)**	**0.1**
Outcome of Primary Challenge					
Paratyphoid Challenge - Paratyphoid Diagnosis	5 (42)	1/5(20%)	22/38(58%)	0.34(0.06-2.04)	0.16
Paratyphoid Challenge - No previous Diagnosis	7 (58)	2/7 (29%)	22/38(58%)	0.49(0.15-1.64)	0.38
**Overall S. Typhi-S.Paratyphi Re-Challenge**	**26 (100)**	**13/26(50%)**	**22/38(58%)**	**0.86(0.54-1.38)**	**0.61**
Outcome of Primary Challenge					
Typhoid Challenge - Typhoid Diagnosis	13 (50)	10/13 (77%)	22/38(58%)	1.32(0.89-1.99)	0.32
Typhoid Challenge - No previous Diagnosis	13 (50)	3/13 (23%)	22/38(58%)	0.4(0.14-1.12)	0.05
Previous Vaccine & Typhoid Challenge					
Any Vaccine	15 (58)	7/15 (47%)	22/38(58%)	0.81(0.44-1.48)	0.54
Previous Vi-PS	3 (12)	1/3 (33%)	22/38(58%)	0.58(0.11-2.92)	0.57
Previous Vi-TT	8 (31)	5/8 (63%)	22/38(58%)	1.08(0.59-1.97)	0.99
Previous Ty21a	1 (4)	0/1 (0%)	22/38(58%)		
Previous M01ZH09	3 (12)	1/3 (33%)	22/38(58%)	0.58(0.11-2.92)	0.57
No Previous Vaccine	11 (42)	6/11(55%)	22/38(58%)	0.94(0.52-1.72)	0.99

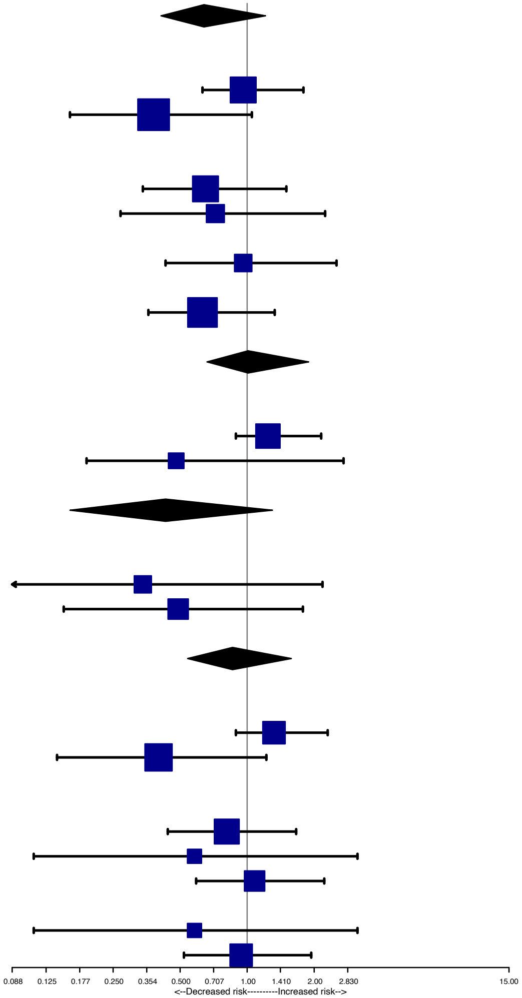

Supplement: S9 Fig — Forest plot comparing relative risk of typhoid or paratyphoid diagnosis following re-challenge compared with naïve and unvaccinated controls challenged with wild-type strains in all challenge studies[20–23,35]. Box-plots represent relative risk and 95% confidence intervals scaled according to size of sub-group. Diamonds represent combined relative risk of diagnosis in each of the re-challenge cohorts. P = Fishers exact test. (PDF) [file pntd.0008783.s015.pdf]
