# Supplementary material for: Homologous and heterologous re-challenge with Salmonella Typhi and Salmonella Paratyphi A in a randomised controlled human infection model
Source: PLoS Negl Trop Dis. 2020 Oct 20;14(10):e0008783. doi: 10.1371/journal.pntd.0008783 (PMC7598925; doi:10.1371/journal.pntd.0008783)
Supplement: S1 Methods — (DOCX) [file pntd.0008783.s020.docx]

**Supplementary methods**

##

## Inclusion and Exclusion criteria

Inclusion Criteria

Participants must satisfy all of the following criteria to be considered eligible for the study:

- Agree to give informed consent for participation in the study.
- Aged between 18 and 60 years inclusive at time of challenge.
- In good health as determined by medical history, physical examination and clinical judgment of the investigators.
- Agree (in the Investigator’s opinion) to comply with all study requirements, including capacity to adhere to good personal hygiene and infection control precautions.
- Agree to allow his or her General Practitioner (and/or Consultant if appropriate), to be notified of participation in the study.
- Agree to allow Public Health England to be informed of their participation in the study^[[1]](#footnote-1)^.
- Agree to give his or her close household contacts written information informing them of the participants’ involvement in the study and offering them voluntary screening for *S.* Typhi or *S*. Paratyphi carriage^*^.
- Agree to have 24-hour contact with study staff during the four weeks post challenge and are able to ensure that they are contactable by mobile phone for the duration of the challenge period until antibiotic completion^*^.
- Have internet access to allow completion of the e-diary and real-time safety monitoring.
- Agree to avoid antipyretic/anti-inflammatory treatment until advised by a study doctor or until at least 14 days after challenge.
- Willing to undergo endoscopy and biopsy.
- Agree to provide their National Insurance/Passport number for the purposes of TOPS registration and bank details for the purpose of reimbursement for the duration of their participation. Bank details will be stored electronically for the duration of the study.

Additional Inclusion Criteria for Re-Challenge

Participants must satisfy all of the above inclusion criteria as well as the following to be considered eligible for the study:

- Have been challenged with *S.* Typhi or *S.* Paratyphi within the last five years during which they were seen at least up to and including the Day 28 visit (completion of challenge and antibiotic treatment).

Exclusion Criteria

The participant will not be enrolled if any of the following apply:

- History of significant organ/system disease that could interfere with study conduct or completion. Including, for example, but not restricted to:
  - Cardiovascular disease
  - Respiratory disease
  - Haematological disease
  - Endocrine disorders
  - Renal or bladder disease, including history of renal calculi
  - Biliary tract disease, including biliary colic, asymptomatic gallstones, previous cholecystectomy, or abnormal ultrasound of the gallbladder
  - Gastro-intestinal disease including requirement for antacids, H_2_-receptor antagonists, proton pump inhibitors or laxatives
  - Neurological disease
  - Metabolic disease
  - Autoimmune disease
  - Psychiatric illness requiring hospitalisation or known or suspected drug and/or alcohol misuse (alcohol misuse defined as an intake exceeding 42 units per week)
  - Infectious disease
- Have any known or suspected impairment of immune function, alteration of immune function, or prior immune exposure that may alter immune function resulting from, for example:
  - Congenital or acquired immunodeficiency, including IgA deficiency
  - Human Immunodeficiency Virus infection
  - Receipt of immunosuppressive therapy such as anti-cancer chemotherapy or radiation therapy within the preceding 12 months or long-term systemic corticosteroid therapy
  - Receipt of immunoglobulin or any blood product transfusion within three months of study start
  - History of cancer (except squamous cell or basal cell carcinoma of the skin and cervical carcinoma in situ)
- Moderate or severe depression or anxiety as classified by the Hospital Anxiety and Depression Score at screening or challenge that is deemed clinically significant by the study investigators^[[2]](#footnote-2)^.
- Weight 50kg or less^[[3]](#footnote-3)^.
- Presence of implants or prosthesis^[[4]](#footnote-4)^.
- Have previously received any typhoid vaccine^[[5]](#footnote-5)^
- Have previously been diagnosed with laboratory confirmed typhoid or paratyphoid infection, or been given a diagnosis compatible with enteric fever5
- Have participated in previous typhoid or paratyphoid challenge studies with ingestion of challenge agent^5^
- Any contraindication to elective upper GI endoscopy (e.g. history of oesophageal perforation, recent myocardial infarction, anticoagulation, pharyngeal diverticulum, head and neck surgery).
- More than one non-study related upper GI endoscopy within the last year.
- Anyone taking long-term medication (e.g. analgesia, anti-inflammatories or antibiotics) that may affect symptom reporting or interpretation of the study results.
- Contra-indication to taking azithromycin, ciprofloxacin, trimethoprim/sulfamethoxazole and/or beta lactam antibiotics.
- Female participants who are pregnant or lactating.
- Female participants who are unwilling to ensure that they or their partner use effective contraception one month prior to challenge and continue to do so until two negative stool samples, a minimum of three weeks after completion of antibiotic treatment, have been obtained.
- Full-time, part-time or voluntary occupations involving:
  - Clinical or social work with direct contact with young children (defined as those attending pre-school groups or nursery or aged under 2 years)^[[6]](#footnote-6)^, or
  - Clinical or social work with direct contact with highly susceptible patients or persons in whom typhoid or paratyphoid infection would have particularly serious consequences e.g. the elderly or infirm (unless willing to avoid work until demonstrated not to be infected with *S*. Typhi or *S*. Paratyphi in accordance with guidance from Public Health England and willing to allow us to inform their employer) ^**^.
- Full time, part time or voluntary occupations involving:
  - Commercial food handling (involving preparing or serving unwrapped foods not subjected to further heating).
- Close household contact with:
  - Young children (defined as those attending pre-school groups, nursery or those aged less than 2 years)
  - Individual(s) who is (are) immunocompromised.
- Scheduled elective surgery or other procedures requiring general anaesthesia during the study period.
- Participants who have participated in another research study involving an investigational product that might affect risk of typhoid or paratyphoid infection or compromise the integrity of the study within the 30 days prior to enrolment (e.g. significant volumes of blood already taken in previous study)^[[7]](#footnote-7)^.
- Detection of any significantly abnormal results from screening investigations (at the clinical discretion of the study investigators).
- Inability to comply with any of the study requirements (at the discretion of the study investigators and the participants General Practitioner).
- Any other social, psychological or health issues which, in the opinion of the study investigator, may:
  - Put the participants or their contacts at risk because of participation in the study, or
  - Adversely affect the interpretation of the primary endpoint data, or
  - Impair the participant’s ability to participate in the study.
- Having been resident in an enteric fever endemic country for six months or more.
- Currently on the study delegation log (Other exclusions for relatives of study team will be done at investigators discretion).

Temporary exclusion criteria at challenge (Both naïve and re-challenge)

Participants will be temporarily excluded from challenge if presenting at the challenge visit with the following:

- Significant acute or acute-on-chronic infection within the previous seven days or have experienced fever (>37.5°C) or subjective febrile symptoms within the previous three days.
- History of any antibiotic therapy during the previous 14 days.
- Any systemic corticosteroid (or equivalent) treatment in the previous 14 days, or for more than seven consecutive days within the past three months.
- Received any vaccine within the last four weeks.
- Recent significant blood donation (e.g., to the National Blood Service).

## Secondary outcome definitions

Secondary clinical and microbiological outcome variables are listed by below

- Time to diagnosis - Time from date/time of challenge to date/time of first temperature ≥38⁰C that subsequently lasted for ≥12hours OR the date/time of first positive blood culture collection (whichever occurs earliest).
- Time to first blood culture positive for *S*. Typhi/Paratyphi - Time from date/time of challenge to date/time of blood culture collection.
- Time to clinical diagnosis (fever ≥38^o^C lasting ≥ 12 hours) - Time from date/time of challenge to date/time of first recorded temperature ≥38^o^C which subsequently lasted 12 hours.
- Mode of diagnosis - The proportion of participants diagnosed with typhoid/paratyphoid fever based upon either clinical criteria (persistent fever ≥38^o^C for ≥ 12 hours) OR microbiological criteria (blood culture positive for S. Typhi/Paratyphi collected ≥ 72 hours from diagnosis)
- Detailed mode of diagnosis - Proportion of participants diagnosed with typhoid/paratyphoid fever based upon either of the following specific diagnostic criteria:
  - Temperature ≥38^o^C preceding positive blood culture;
  - Temperature ≥38^o^C without positive blood culture;
  - S. Typhi/Paratyphi bacteraemia preceding temperature ≥38^o^C;
  - S. Typhi/Paratyphi bacteraemia without temperature ≥38^o^C.
- Time to first fever - Time from date/time of challenge to date/time of first recorded temperature ≥38⁰C.
- Fever clearance time - Time from initiation of antibiotics or start of fever (whichever was later) to first recorded temperature <38^o^C persisting for at least 48hours. Only diagnosed participants with fever were included in the analysis.
- Symptom severity - Severity of symptoms in each challenge group were assessed by:
- The proportion of participants with maximum symptom severity score graded as mild, moderate or severe following challenge.
- The proportion of participants meeting the criteria for severe enteric fever.
  - Individual enteric fever severity scores calculated by summing numerical values assigned to the severity of individual solicited symptoms, clinical observations (heart rate, systolic blood pressure, diastolic blood pressure and temperature) and laboratory measurements between Day 0 to Day 21 (0=not present; 1=mild; 2=moderate; 3=severe; 4 = Hospitalisation.
  - Duration of bacteraemia - Time (Hours/Days) from collection of first positive blood culture until date/time of the last positive blood culture.
  - Bacteraemia clearance time - Time (Hours/Days) from collection of first positive blood culture until date/time of the first negative blood culture remaining persistently negative. Participants with missing data (e.g. no negative blood cultures after commencing antibiotics) were censored in the analysis at the time point of the last culture taken.
  - Stool shedding - Daily stool culture(s) positive for S. Typhi/Paratyphi for 14 days post-challenge.
- Quantitative blood culture - Concentration of bacteria in 10ml blood taken at the time of diagnosis using the Wampole™ Isostat® Isolator system (Colony forming units/ml). For values below the lower limit of detection (0.1 CFU/ml), a value of 0.05 CFU/ml was assigned.
- Haematological and biochemical end-points - The following haematological parameters were measured from time of challenge to Day 28 and/or Day 90.
  - Total Haemoglobin (g/L)
  - Haemoglobin change from baseline (Hb g/l D0 – Hb g/l D14)
  - Total White Cell Count (x109/l)
  - Platelet counts (x109/l)
  - Neutrophil count (x109/l)
  - Lymphocyte count (x109/l)
  - Monocyte count (x109/l)
  - Eosinophil count (x109/l)
  - Monocyte/Lymphocyte ratio
  - Urea & Electrolytes (Na, K+, Urea, Creatinine –mmol/l)
  - C-reactive protein (mg/l)
  - Liver function tests (Bilirubin [umol/l], aspartate transaminase (AST IU/l), alkaline phosphatase (ALP IU/l), alanine transaminase (ALT IU/l), Albumin (g/L)
- Safety outcome measures - Adverse events, adverse events of special interest, SAE’s and SUSARs according to each study group.

## Solicited Symptoms and Grading

| Solicited Symptoms & Expected Adverse Events of Enteric Fever | Grade 0 | Grade 1 | Grade 2 | Grade 3 | Grade 4 |
| --- | --- | --- | --- | --- | --- |
| Headache | Not present | Present but no interference with activity | Some interference with activity | Significant; any use of codeine phosphate or prevents daily activity | Hospital visit required |
| Malaise | Not present | Present but no interference with activity | Some interference with activity | Significant; any use of codeine phosphate or prevents daily activity | Hospital visit required |
| Anorexia | Not present | Eat less than normal for 1-2 meals | Miss 1-2 meals completely | Miss all meals completely | Hospital visit required |
| Rash | Not present | Present but no interference with activity | Some interference with activity | Significant or prevents daily activity | Hospital visit required |
| Constipation | Not present | Present but no interference with activity | Some interference with activity | Significant or prevents daily activity | Hospital visit required |
| Diarrhoea | Not present | 3 – 4 loose stools in 24 hours (≥200mls volume) | 5 – 6 loose stools in 24 hours (≥200mls volume) | >6 loose stools in 24 hours (≥200mls volume) | Hospital visit required |
| Abdominal pain/distension | Not present | Present but no interference with activity | Some interference with activity | Significant or prevents daily activity | Hospital visit required |
| Myalgia | Not present | Present but no interference with activity | Some interference with activity | Significant or prevents daily activity | Hospital visit required |
| Arthralgia | Not present | Present but no interference with activity | Some interference with activity | Significant or prevents daily activity | Hospital visit required |
| Cough | Not present | Present but no interference with activity | Some interference with activity | Significant or prevents daily activity | Hospital visit required |
| Nausea and/or vomiting | Not present | Present but no interference with activity or 1 – 2 episodes in 24 hours | Some interference with activity or more than 2 episodes in 24 hours | Significant or prevents daily activity | Hospital visit required |

## Criteria for severe enteric fever

| Severe enteric fever is diagnosed if ANY of the following apply |
| --- |
| Oral temperature > 40ºC |
| Systolic blood pressure < 85 mmHg |
| Significant lethargy or confusion |
| Gastrointestinal bleeding |
| Gastrointestinal perforation |
| Any grade 4 laboratory abnormality |

Criteria for severe enteric fever

## Serum aNTIBODY ELISA

Antibody responses to *S*. Typhi O9:LPS (*S*. Typhosa LPS, L2387; Sigma-Aldrich, Dorset, UK), *S*. Typhi H-d antigen (University of Maryland CV0150622) and *S*. Paratyphi O:2 antigen (GSK Vaccines for global health) were measured using an in-house ELISA. Ninety-six well flat-bottom ELISA plates (Nunc 442404, Thermo Fisher) were coated 100μl/well of the target antigens suspended in 50mM carbonate-bicarbonate buffer (final concentration 15μg/ml O9:LPS; 1μg/ml H-d antigen and 1μg/ml O2 antigen), sealed and stored overnight at 4°C. Plates were washed five times with 300μl/well PBS + 0.05% Tween®20, before being blocked with 200μl/well PBS + 1 % bovine serum albumin (BSA) at room temperature for 90 minutes. After blocking, plates were washed five times with PBS + 0.05% Tween®20 and subsequently incubated at ambient temperature for 90 mins with 100μl/well of test serum samples diluted to 1:200, 1:600, 1:1800, 1:5400. All samples were run in duplicate. After incubation with the primary antibody, plates were again washed five times with PBS + 0.05% Tween®20 and subsequently incubated with secondary antibody at room temperature for 60 minutes (goat anti-human IgG:HRP at 1:20,000 [Bio Rad 204005] or goat anti-human IgA:HRP [Bio Rad STR141] at 1:6,000). After washing, plates were developed by adding 100μl/well of 3,3’, 5, 5’-Tetramethylbenzidine (TMB T0440, Sigma Aldrich, UK) and incubating out of direct light at room temperature for ~20-30 minutes, until absorbance at 630nm reading for the top concentration of standard curve reached 0.5-0.55. The reaction was stopped by adding 50μl/well of H_2_SO_4_. The final absorbances were read at 450 nm and 630nm using an automated microplate reader (Biotek ELx808 and associated Gen software v5.2).

The standard curve was generated by preparing a serial dilution of standard serum per plate. The standard serum was supplied by Emergent BioSolutions® from volunteers vaccinated with the live oral typhoid vaccine MO1ZH09. A standard serum of nominal concentration of 30,000 units/ml was made by adding 200μl of positive sera with 170μl of LPS negative sera, also supplied by Emergent BioSolutions®. From this, an intermediate positive control serum (IPCS), with a nominal concentration of 300units/ml, was prepared by mixing 300μl of standard sera with 29.7ml PBS/BSA, stored in 1ml aliquots at -20°C. We added 200μL/well of standard serum in duplicate at a starting concentration of 1/100, which was two-fold serial diluted to 1/12800 with PBS/BSA. High, intermediate and low-quality control (QC) sera were also prepared using IPCS standard sera to defined concentrations varying according to test antigen, respectively, and 100ul/well was added to the plate. PBS/BSA alone was used in the blank control wells and plates were incubated at RT for 1-hour.

An R^2^ value for the standard curve of each plate was calculated and the plate rejected if the R^2^ < 0.98. Additionally, the plate was rejected if the average blank OD value was > 0.1. The results of each ELISA plate were accepted only if the OD values of the high, intermediate and low-quality control wells were within the range of their known values ± two standard deviations of the blank wells. Plates were rejected if the within-dilution percentage coefficient of variance (%CV) of the three QC dilutions was > 10% or, if the mean values for the three QC dilutions were outside the predetermined ranges.

For test sera sample to be accepted, at least 2 consecutive serial dilutions with a within-dilution %CV of < 20% for the duplicates of each dilution needed to be present. If samples fell into the bottom range of the standard curve close to the detection limit (absorbance LLQ) a within-dilution %CV of < 35% was accepted if no further dilution was available.

## supporting References

1. Liamis G, Liberopoulos E, Barkas F, Elisaf M. Spurious Electrolyte Disorders: A Diagnostic Challenge for Clinicians. Am J Nephrol. 2013;38: 50–57. doi:10.1159/000351804

2. Waddington CS, Darton TC, Jones C, Haworth K, Peters A, John T, et al. An outpatient, ambulant-design, controlled human infection model using escalating doses of Salmonella Typhi challenge delivered in sodium bicarbonate solution. Clin Infect Dis. 2014;58: 1230–40. doi:10.1093/cid/ciu078

3. Darton TC, Jones C, Blohmke CJ, Waddington CS, Zhou L, Peters A, et al. Using a Human Challenge Model of Infection to Measure Vaccine Efficacy: A Randomised, Controlled Trial Comparing the Typhoid Vaccines M01ZH09 with Placebo and Ty21a. PLoS Negl Trop Dis. 2016;10: e0004926. doi:10.1371/journal.pntd.0004926

4. Dobinson HC, Gibani MM, Jones C, Thomaides-Brears HB, Voysey M, Darton TC, et al. Evaluation of the clinical and microbiological response to salmonella paratyphi a infection in the first paratyphoid human challenge model. Clin Infect Dis. 2017;64. doi:10.1093/cid/cix042

5. Jin C, Gibani MM, Moore M, Juel HB, Jones E, Meiring J, et al. Efficacy and immunogenicity of a Vi-tetanus toxoid conjugate vaccine in the prevention of typhoid fever using a controlled human infection model of Salmonella Typhi: a randomised controlled, phase 2b trial. Lancet. 2017;390: 2472–2480.

6. Gibani M, Jin C, Thomaides-Brears H, Shrestha S, Precaido-Llanes L, Napolitani G, et al. Investigating Systemic Immunity to Typhoid and Paratyphoid Fever: Characterising the Response to Re-challenge in a Controlled Human Infection Model. Open Forum Infect Dis. Oxford University Press; 2017;4: S227–S228. doi:10.1093/ofid/ofx163.474

7. Gibani MM, Jones E, Barton A, Jin C, Meek J, Camara S, et al. Investigation of the role of typhoid toxin in acute typhoid fever in a human challenge model. Nat Med. Nature Publishing Group; 2019;25: 1082–1088. doi:10.1038/s41591-019-0505-4

# supplementary Tables

S1 Table - Serious adverse events OVG2014/01 study.

| **Challenge Group** | **Criteria** | **Event** | **Challenge related** | **Comment** |
| --- | --- | --- | --- | --- |
| ***S.* Typhi (Naïve)** | In-patient hospitalisation or prolongation | Collapse | No | Episode of collapse with loss of consciousness 12 months after challenge. Reported left arm and left leg weakness 24 hours’ duration. Diagnosed as possible generalised seizure following neurology review. Instructed not to drive for 6/12. No medication prescribed, and no subsequent events. |
| ***S.* Paratyphi (Naïve)** | In-patient hospitalisation or prolongation | Hospital admission for IV fluids | Yes | Nausea and vomiting, unresponsive to oral anti-emetics. Tachycardia. Admitted overnight for IV fluids and anti-emetics. Treatment switched to IV Ceftriaxone. Discharged after <24 hours and completed course of oral ciprofloxacin. |
| **NA** | Medically important event | Renal Colic | No | Right loin pain with evidence of renal calculus on CT KUB. Diagnosed after enrolment but prior to challenge. Withdrawn from study and not challenged. |
| ***S.* Paratyphi (Heterologous re-challenge ST-SPT)** | Medically important event | Raised ALT | Yes | Alanine aminotransferase elevated to 898 IU/L 5 days after diagnosis. Ascribed to paratyphoid fever plus possible adverse drug reaction (azithromycin + paracetamol). Antibiotic switched from azithromycin to ciprofloxacin. Paracetamol withheld. Resolved. |

S2 Table - Participants meeting pre-specified criteria for severe enteric fever. Hypokalaemia was observed in four participants, which was attributed to pseudohypokalaemia resulting from delayed sample transport over periods of high ambient temperature[1]

| **Challenge Group** | **Criteria** | **Event** | **Challenge related** | **Comment** |
| --- | --- | --- | --- | --- |
| ***S.* Typhi Naïve** | Grade 4 laboratory abnormality | Hypokalaemia | No | K+ 2.9 mmol/l  At time of typhoid diagnosis |
| ***S.* Typhi Naïve** | Grade 4 laboratory abnormality | Hypokalaemia | No | K+ 2.8 mmol/l  24 hours after typhoid diagnosis |
| ***S.* Paratyphi (Heterologous re-challenge ST-SPT)** | Grade 4 laboratory abnormality | Hypokalaemia | No | K+ 2.9 mmol/l |
| ***S.* Paratyphi (Heterologous re-challenge ST-SPT)** | Grade 4 laboratory abnormality | Hypokalaemia | No | K+ 2.8 mmol/l |
| ***S.* Typhi (Heterologous re-challenge SPT-ST)** | Grade 4 laboratory abnormality | Elevated CRP | Yes | CRP 231mmol/l 48 hours after typhoid diagnosis |

| Study | Primary Challenge | Number Eligible  for Re-Challenge | Number (%) Enrolled  for Re-Challenge |
| --- | --- | --- | --- |
| OVG2009/10 (T1) [2] | *S*. Typhi Quailes strain | 40 | 14 (35%) |
| OVG2011/02 (T2)  NCT01405521 [3] | *S.* Typhi Quailes strain | 94 | 15 (16%) |
| OVG2013/07 (P1)  NCT02100397 [4] | *S.* Paratyphi A NVGH308 strain | 40 | 22 (55%) |
| OVG2014/08 (VAST)  NCT02324751 [5] | *S.* Typhi Quailes strain | - | 23 |
| OVG2014/01 (PATCH)  NCT02192008 [6] | *S.* Paratyphi A NVGH308 strain/  *S.* Typhi Quailes strain | - | 3 |

S3 Table - Number (%) of participants enrolled for re-challenge from previous challenge studies. Participants from the OVG2014/01 (n=113) and OVG2014/08 (n=103) studies were eligible for re-challenge after 12 months had elapsed from their primary challenge. As these studies were running contemporaneously, only a proportion of participants from these studies were eligible for re-challenge by the end of recruitment.

|  | Challenge Group | | | | | |
| --- | --- | --- | --- | --- | --- | --- |
|  | S. Typhi Challenge | | | S. Paratyphi Challenge | | |
|  | ST Naïve | ST-ST Re-Challenge | SPT-ST Re-Challenge | SPT Naïve | SPT-SPT Re-Challenge | ST-SPT Re-Challenge |
| Composite Criteria, n (% attack rate) | 12/19  (63%) | 12/27  (44%) | 7/10  (70%) | 10/18  (56%) | 3/12  (25%) | 13/26  (50%) |
| RR (95% CI)  Compared with naïve controls | Ref. | 0.7  (0.40-1.23) | 1.10  (0.59-1.87) | Ref. | 0.15  (0.15-1.14) | 0.90  (0.51-1.58) |
| p | Ref. | 0.24 | 0.99 | Ref. | 0.14 | 0.76 |
| Fever ≥38˚C (any duration) | 9/19  (47%) | 9/27  (33%) | 3/10  (30%) | 6/18  (33%) | 1/12  (8%) | 8/26  (31%) |
| RR (95% CI)  Compared with naïve controls | Ref. | 0.70  (0.35-1.45) | 0.63  (0.21-1.60) | Ref. | 0.25  (0.04 – 1.29) | 1.1  (0.45-2.58) |
| p | Ref. | 0.37 | 0.44 | Ref. | 0.19 | 0.99 |
| Fever ≥38·0˚C (any duration) + *S.* Typhi bacteraemia | 9/19  (47%) | 7/27  (26%) | 3/10  (30%) | 5/18  (27%) | 1/12  (8%) | 8/26  (31%) |
| RR (95% CI)  Compared with naïve controls | Ref. | 0.54  (0.25-120) | 0.63  (0.21-1.60) | Ref. | 0.3  (0.05-1.61) | 0.90  (0.35 -2.31) |
| p | Ref. | 0.20 | 0.44 | Ref. | 0.35 | 0.99 |
| Fever ≥38·0˚C (any duration) with subsequent bacteraemia | 0/19  (0%) | 4/27  (15%) | 0/10  (0%) | 2/18  (11%) | 1/12  (8%) | 2/26  (8%) |
| RR (95% CI)  Compared with naïve controls | Ref. | - | - | Ref. | 0.75  (0.10 – 5.14) | 0.69  (0.11 – 4.4) |
| p | Ref. | - | - | Ref. | 0.99 | 0.99 |
| *S*. Typhi bacteraemia OR stool shedding | 14/19  (74%) | 20/27  (74%) | 9/10  (90%) | 14/18  (77%) | 5/12  (42%) | 15/26  (58%) |
| RR (95% CI)  Compared with naïve controls | Ref. | 1  (0.71-1.51) | 1.22  (0.77 – 1.80) | Ref. | 0.75  (0.10 – 5.14) | 0.74  (0.49 – 1.12) |
| p | Ref. | 0.99 | 0.63 | Ref. | 0.06 | 0.20 |
| *S*. Typhi bacteraemia AND stool shedding | 7/19 (37%) | 6/27  (22%) | 7/10  (70%) | 4/18 (22%) | 2/12  (17%) | 7/26  (27%) |
| RR (95% CI)  Compared with naïve controls | Ref. | 0.6  (0.24-1.49) | 1.9  (0.9 – 3.9) | Ref. | 0.75  (0.18-2.96) | 1.21  (0.41-3.53) |
| p | Ref. | 0.33 | 0.12 | Ref. | 0.99 | 0.99 |
| Any Shedding | 9/19 (47%) | 15/27 (56%) | 9/10  (90%) | 9/18 (50%) | 4/12  (33%) | 9/26  (35%) |
| RR (95% CI)  Compared with naïve controls | Ref. | 1.17  (0.67-2.19) | 1.9  (1.10 – 3.37) | Ref. | 0.67  (0.25-1.54) | 0.69  (0.34 – 1.40) |
| p | Ref. | 0.76 | 0.043 | Ref. | 0.47 | 0.36 |

S4 Table - Attack rates according to alternative diagnostic criteria. ST = *S*. Typhi. SPT = *S*. Paratyphi A. p = Fishers exact test.

S5 Table - *Salmonella* Typhi and Paratyphi controlled human infection studies conducted in Oxford 2011- 2017.

| Study | Challenge agent | Study type | Vaccine | Description | Ref |
| --- | --- | --- | --- | --- | --- |
| OVG2009/10  (T1) | *S*. Typhi Quailes strain | Observational | - | Dose finding study  Low dose: 1-5 x 10^3^ CFU (n=20)  High dose: 1-5 x 10^4^ CFU (n=20) | [2] |
| OVG2011/02  NCT01405521  (T2) | *S*. Typhi Quailes strain | Vaccine RCT | M01ZH09 (n=31)  Ty21a (n=30)  Placebo (n=30) | Ty21a or M01ZH09 vaccines compared with control.  Challenge dose 1-5 x 10^4^ CFU | [3] |
| OVG2013/07  NCT02100397  (P1) | *S.* Paratyphi A NVGH308 strain | Observational | - | Dose finding study  High dose: 1-5 x 10^3^ CFU (n=20)  Low dose: 0.5-1 x 10^3^ CFU (n=20) | [4] |
| OVG2014/08  NCT02324751 | *S.* Typhi Quailes strain | Vaccine RCT | Vi-Polysaccharide (n=35)  Vi-TT conjugate (n=37)  Placebo (n=31) | Vi-PS (Typhim Vi ®, Sanofi Pasteur) or Vi-TT (TypbarTCV ®, Bharat Biotech) vaccines compared with control.  Challenge dose 1-5 x 10^4^ CFU | [5] |
| OVG2014/01  NCT02192008 | *S.* Paratyphi A NVGH308 strain  *S.* Typhi Quailes strain | RCT | - | Naïve challenge (S. Typhi and S. Paratyphi) vs Re-challenge (homologous and heterologous)  S. Typhi challenge dose 1-5 x 10^4^ CFU  S. Paratyphi challenge dose 1-5 x 10^3^ CFU | [6] |
| OVG2016/03  NCT03067961 | *S.* Typhi Quailes strain/  *S*. Typhi SB6000 (Typhoid-toxin deficient) | RCT | - | Wild Type S. Typhi Quailes strain (n=20)  SB6000 Typhoid toxin negative strain (n=20)  Challenge dose 1-5 x 10^4^ CFU | [7] |

# Supplementary Figures

S1 Figure - Study profile. Participants were recruited into one of three study groups defined *a priori* according to prior challenge status.

S2 Figure - Recruitment into re-challenge cohort. Density plot according to previous challenge agent allocation. Vertical lines represent median re-challenge interval for participants previously challenged with S. Typhi (orange) and S. Paratyphi (grey)

S3 Figure - Combined Attack Rates in *S*. Typhi and *S*. Paratyphi challenge studies. Forest plot illustrating attack rates in naive cohorts of *S*. Typhi (top) and *S*. Paratyphi (bottom) challenge studies. Heterogeneity I-squared = 0, test of homogeneity (Q statistic) gives p value for 0.78 (no evidence the proportions vary).

S4 Figure - Time to Diagnosis after homologous re-challenge and combined naïve historical controls. Cumulative incidence of typhoid (i) and paratyphoid A (ii) fever after challenge in naïve (ST& SPT) and homologous re-challenge (ST-ST & SPT-SPT) groups. Time to composite diagnostic endpoint, measured from challenge agent ingestion to development of first fever ≥38°C or first positive blood culture sampling. Non-diagnosed participants censored at day 14 hours. P value from log-rank test comparing ST = S. Typhi naïve challenge. ST-ST = Homologous Re-Challenge with S. Typhi. SPT = S. Paratyphi naïve challenge. SPT-SPT = Homologous Re-Challenge with S. Paratyphi.

S5 Figure – Haematology (a & b) and Biochemistry (c & d) laboratory parameters post challenge according to diagnosis status. Timepoints in a and c are normalised to the day of diagnosis (ED day = 0). Timepoints b and d in non-diagnosed participants represent day of sample collection. Box-and whisker plots represent median and interquartile range. Solid coloured lines link median value at each time point. Grey lines connect paired data points from the same individuals. Units: Haemoglobin = (g/dL); Haemoglobin change g/dL compared with Hb Day 0; Haematocrit(L/L); White cell count/Neutrophil count/Lymphocyte count/Eosinophil count/Monocyte count = cells x 10^9^/L; Urea = mmol/L; Creatinine = mg/L; Na^+^/K^+^ = mEq/L; C-reactive protein = mmol/l; Bilirubin = umol/l; ALT – IU/l; ALP = U/L; Albumin = g/L.

S6 Figure - Pattern of bacteraemia following *S.* Typhi (A) and *S.* Paratyphi A (B) challenge. Each row corresponds to an individual participant. Grey squares = Negative sample, Red squares = Positive blood culture, White squares = No sample collected. Tx = Day of treatment initiation. Participants above the dotted line did not meet the composite criteria for typhoid or paratyphoid diagnosis.

S7 Figure - Pattern of stool shedding after *S*. Typhi and *S*. Paratyphi challenge. Each row corresponds to an individual participant. Grey squares = Negative sample, Brown squares = Positive stool culture, White squares = No sample collected. Tx = Day of treatment initiation. Participants above the dotted line did not meet the composite criteria for typhoid diagnosis.

S8 Figure - Sub-Group analysis OVG2014/01 study. Forest plot comparing relative risk of typhoid or paratyphoid diagnosis following re-challenge compared with naïve controls from OVG2014/01 study. Box-plots represent relative risk and 95% confidence intervals scaled according to size of sub-group. Diamonds represent combined relative risk of diagnosis in each of the re-challenge cohorts. P = Fishers exact test.

S9 Figure – Sub group analysis. Forest plot comparing relative risk of typhoid or paratyphoid diagnosis following re-challenge compared with naïve and unvaccinated controls challenged with wild-type strains in all challenge studies[3–7]. Box-plots represent relative risk and 95% confidence intervals scaled according to size of sub-group. Diamonds represent combined relative risk of diagnosis in each of the re-challenge cohorts. P = Fishers exact test.

S10 Figure - Baseline (Day 0) serum anti-O9:LPS (a) anti-Hd (b) and anti-Vi (c) IgG in participants challenged with S. Typhi, grouped according to (i) outcome of challenge (ii) challenge group and (iii) outcome of previous challenge. p = Mann-Whitney U test two sided; Box plots display median, interquartile range; ST = S. Typhi naïve; ST-ST = Homologous S. Typhi re-challenge. SPT-ST = Heterologous S. Typhi re-challenge. ED = Met criteria for enteric fever diagnosis. nED = Did not meet criteria for enteric fever diagnosis

S11 Figure - Baseline (Day 0) serum anti-O2:LPS IgG (a) and IgA in participants challenged with *S*. Paratyphi, grouped according to (i) outcome of challenge (ii) challenge group and (iii) outcome of previous challenge. p = Mann-Whitney U test two sided; Box plots display median, interquartile range; SPT = S. Paratyphi naïve; SPT-SPT = Homologous S. Paratyphi re-challenge. ST-SPT = Heterologous S. Paratyphi re-challenge. ED = Met criteria for enteric fever diagnosis. nED = Did not meet criteria for enteric fever diagnosis.

S12 Figure - *Antibody response to Salmonella* Typhi *(a) and* Paratyphi *A (b) antigens following challenge/re-challenge with Salmonella* Typhi *(a) and* Paratyphi *(b).* Grouped according to antigen and outcome (ED = Met criteria for enteric fever diagnosis. nED = Did not meet criteria for enteric fever diagnosis). Coloured lines connect median values for each timepoint. Grey lines connect paired samples across timepoints; Box plots display median, interquartile range; p = Mann-Whitney test.

# Meta-Analysis Summary of Previous S. Typhi & S. Paratyphi challenge studies

Meta-analysis summary  - S. Typhi                Number of studies =      5

Fixed-effects model                       Heterogeneity:

Method: Inverse-variance                            I2 (%) =    0.00

                                                        H2 =    0.44

--------------------------------------------------------------------

           Study     |     ES    [95% Conf. Interval]     % Weight

---------------------+---------------------------------------------------

1                    |      0.65        0.41         0.85   15.03

2                    |      0.67        0.47         0.83   23.09

3                    |      0.77        0.59         0.90   30.32

4                    |      0.63        0.38         0.84   13.96

5                    |      0.71        0.48         0.89   17.60

---------------------+---------------------------------------------------

Fixed pooled  ES     |      0.70        0.62         0.78  100.00

---------------------+---------------------------------------------------

Test of theta = 0: z = 16.93                     Prob > |z| = 0.0000

Test of homogeneity: Q = chi2(4) = 1.75            Prob > Q = 0.7818

Meta-analysis summary   - S. Paratyphi         Number of studies =      2

Fixed-effects model                       Heterogeneity:

Method: Inverse-variance                            I2 (%) =    0.00

                                                        H2 =    0.08

           Study     |     ES    [95% Conf. Interval]     % Weight

---------------------+---------------------------------------------------

1                    |      0.60        0.36         0.81   53.34

2                    |      0.56        0.31         0.78   46.66

---------------------+---------------------------------------------------

Fixed pooled  ES     |      0.58        0.42         0.74  100.00

---------------------+---------------------------------------------------

Test of theta = 0: z = 7.24                      Prob > |z| = 0.0000

Test of homogeneity: Q = chi2(1) = 0.08            Prob > Q = 0.7817

1. [↑](#footnote-ref-1)
2. If elevated scores are due to temporary significant life events, the questionnaire may be repeated after resolution of the event with a view to inclusion if normal. [↑](#footnote-ref-2)
3. Or a body mass index (BMI) that, in the opinion of the study team, may adversely impair the interpretation of the study results or affect the safe performance of any study procedure. [↑](#footnote-ref-3)
4. This exclusion criteria does not apply to the negative control group [↑](#footnote-ref-4)
5. For Group A. [↑](#footnote-ref-5)
6. [↑](#footnote-ref-6)
7. As assessed by both participant questioning and registration with The Over-volunteering Prevention System (TOPS) database. [↑](#footnote-ref-7)
