# Supplementary material for: Homologous and heterologous re-challenge with Salmonella Typhi and Salmonella Paratyphi A in a randomised controlled human infection model
Source: PLoS Negl Trop Dis. 2020 Oct 20;14(10):e0008783. doi: 10.1371/journal.pntd.0008783 (PMC7598925; doi:10.1371/journal.pntd.0008783)
Supplement: S1 Protocol — (PDF) [file pntd.0008783.s021.pdf]

Investigating Enteric Fever

Investigating the mechanisms and determinants of systemic and mucosal immunity to *Salmonella* Typhi and *Salmonella* Paratyphi A in naïve and previously exposed individuals – A challenge and rechallenge study

Date and version number	Version 5.0, 22-MAY-2018
Ethics reference	14/SC/1204
Sponsor code	OVG 2014/01
Sponsor	The University of Oxford
Funder	Medical Research Council, UK<br>MRC reference: MR/K021222/1
Clinicaltrials.gov number	NCT02192008
Grant Holder	Prof Vincenzo Cerundolo<br>Professor of Immunology<br>Division of Investigative Medicine<br>Weatherall Institute of Molecular Medicine<br>University of Oxford<br>John Radcliffe Hospital<br>Headley Way<br>Oxford OX3 9DS<br>Tel/Fax: +44 (0) 1865 222 412<br>vincenzo.cerundolo@imm.ox.ac.uk

Investigator Agreement

"I have read this protocol and agree to abide by all provisions set forth therein. I agree to comply with the International Conference on Harmonization Tripartite Guideline on Good Clinical Practice."

Signatures:

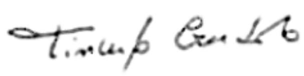

Date: 28th August 2018

Grant Holder:

Professor Vincenzo Cerundolo

Weatherall Institute of Molecular Medicine, University of Oxford

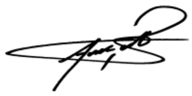

Date: 28th August 2018

Chief Investigator:

Professor Andrew Pollard

Oxford Vaccine Group, Department of Paediatrics, University of Oxford

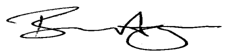

Date: 28th August 2018

Co-Investigator:

Professor Brian Angus

Centre for Tropical Medicine, Nuffield Department of Medicine, University of Oxford

Confidentiality Statement

This document contains confidential information that must not be disclosed to anyone other than the Sponsor, the Investigator Team, host NHS Trust(s), regulatory authorities, and members of the Research Ethics Committee.

1. Contents

1. KEY STUDY CONTACTS 8

2. SYNOPSIS 9

3. BACKGROUND AND RATIONALE 16

4. OBJECTIVES AND ENDPOINTS 22

5. STUDY DESIGN 24

5.1. Part A and Part B 24

5.2. Negative control group 25

5.3. Study overview 25

6. PARTICIPANT IDENTIFICATION 28

6.1. Study Participants 28

6.2. Inclusion Criteria 28

6.3. Additional Inclusion Criteria for Part B 29

6.4. Exclusion Criteria 29

6.5. Temporary exclusion criteria at challenge (Both Part A and B) 32

7. STUDY PROCEDURES 32

7.1. Part A and Part B 33

7.2. Recruitment (Part A and negative control group) 38

7.3. Recruitment (Part B) 39

7.4. Informed Consent 40

7.5. Screening and Eligibility Assessment Visit 41

7.6. Randomisation and Blinding 43

7.7. Post-consent Visits 44

i. Endoscopy (Day -180to Day -45, visit V1) 44

ii. eDiary and first Enteric String Sampling (Day -7, visit V2) 45

iii. Challenge visit (Day 0, Visit V3) 45

iv. Post-challenge visits; participants who do not reach diagnosis (Day 0+12hrs to Day 365, visit V4 to V30) 47

v. Outside of scheduled visits and unscheduled visits 47

vi. Enteric fever diagnosis and enteric fever diagnosis visits (visit 19 to 24) 48

vii. Second endoscopy visit (visit 27) 50

viii. Enteric String Sampling 50

ix. Wireless video capsule endoscopy 51
x. Participant questionnaire 51
7.8. Reporting cases to Public Health England 52
7.9. Stool Culture for Carriage of *S*. Typhi or *S*. Paratyphi 52
7.10. Screening of Household Contacts 52
7.11. Discontinuation/Withdrawal of Participants from Study 53
i. Pregnancy 53
ii. Trial Over-volunteering Prevention System 53
7.12. Definition of End of Study 54
7.13. Potential Benefit to Participants 54
7.14. Potential Risk to Participants 54
i. Phlebotomy 54
ii. Wireless video capsule endoscopy 54
iii. Endoscopy and biopsy 54
iv. Symptomatic infection 55
v. Study-fatigue 56
vi. Complications of typhoid and paratyphoid infection 56
7.15. Potential Risks to Close Contacts of Participants 56
8. INTERVENTIONS 57
***S.* Paratyphi Strain** **8.1.** **58**
i. The challenge strain (NVGH308) 58
ii. GMP manufacture of the challenge strain (NVGH308) 58
iii. Storage of the study treatment 58
8.2. *S.* Typhi Strain 59
i. The challenge strain (Quailes) 59
ii. GMP manufacture of the challenge strain (Quailes) 59
iii. Storage of the study treatment 59
8.3. General considerations for both strains 60
i. Accountability of the study treatment 60
ii. Concomitant medication 60
iii. Concomitant medication for symptoms of enteric fever 60
iv. Antibiotic treatment 60
v. Contraception, antibiotic treatment and enteric fever symptoms 62
vi. Post-study treatment 62

9. LABORATORY 62
9.1. Blinding of laboratory samples 63
9.2. Bacteriology 63
9.3. Molecular diagnostics 64
9.4. Inflammatory responses 65
9.5. Antibody responses 65
9.6. Cellular immune responses 66
9.7. Factors affecting susceptibility and response to infection 66
9.8. Other laboratory investigations 67
10. SAFETY REPORTING 68
10.1. Definitions of an Adverse Event (AE) 68
10.2. Definition of an Adverse Reaction (AR) 68
10.3. Definition of an Expected Adverse Event 68
10.4. Definition of a Serious Adverse Event (SAE) 70
10.5. Definition of a Serious Adverse Reaction (SAR) 71
10.6. Adverse events of special interest (AESI) 71
10.7. Expected Serious Adverse Events and Serious Adverse Reactions 72
10.8. Definition of a Suspected Unexpected Serious Adverse Reaction (SUSAR) 72
10.9. Procedures for recording Adverse Events 72
APPENDIX A: Negative control group .... Error! Bookmark not defined.
10.10. Procedures for reporting Serious Adverse Events 73
10.11. SUSAR Reporting 74
10.12. Annual Progress report (APR) 74
10.13. Interim safety review 74
10.14. Safety Profile Review 74
10.15. Trial Management Committee 74
10.16. Data Safety Monitoring Committee (DSMC) 74
10.17. Stopping rules 75
10.18. Staff and Investigator safety 75
11. STATISTICS AND ANALYSIS 75
11.1. Description of Statistical Methods 75
11.2. Analysis of Outcome Measures/Endpoints 76
11.3. The Level of Statistical Significance 77

11.4. Criteria for the Termination of the Study 77
11.5. Procedure for Accounting for Missing, Unused, and Spurious Data 77
11.6. Inclusion in Analysis 77

12. DATA MANAGEMENT 77
12.1. Access to Data 77
12.2. Data Recording and Record Keeping 78
12.3. Data integrity 78
12.4. Data archiving and storage 78

13. QUALITY ASSURANCE PROCEDURES 78
13.1. Investigator procedures 78
13.2. Monitoring 79
13.3. Source Data 79
13.4. Direct access to source data/documents 79
13.5. Modification to protocol 79
13.6. Protocol deviation 80
13.7. Audit & Inspection 80
13.8. Study Progress 80

14. ETHICAL AND REGULATORY CONSIDERATIONS 80
14.1. Declaration of Helsinki 80
14.2. Guidelines for Good Clinical Practice 80
14.3. Approvals 80
14.4. Reporting 81
14.5. Participant Confidentiality 81
14.6. Reimbursement 81

15. FINANCE AND INSURANCE 82
15.1. Funding 82
15.2. Insurance 82

16. PUBLICATION POLICY 82

17. REFERENCES 83

APPENDIX A: Negative control group 88

APPENDIX B: Grading the severity of solicited and unsolicited systemic Adverse Events 91

APPENDIX C: Grading the severity of visit observed Adverse Events 92

APPENDIX D: Grading the severity of laboratory Adverse Events 93

APPENDIX E: Close Household contacts 94

APPENDIX F: Amendment history 95

1. KEY STUDY CONTACTS

Grant Holder	Prof Vincenzo Cerundolo<br>Professor of Immunology / Unit Director<br>Weatherall Institute of Molecular Medicine,<br>University of Oxford,<br>John Radcliffe Hospital,<br>Headley Way, Oxford OX3 9DS, United Kingdom.<br>vincenzo.cerundolo@imm.ox.ac.uk
Chief Investigator	Prof Andrew Pollard<br>Professor of Paediatric Infection and Immunity / Director<br>Oxford Vaccine Group,<br>University of Oxford,<br>Centre for Clinical Vaccinology & Tropical Medicine (CCVTM),<br>Churchill Hospital, Oxford OX3 7LE, United Kingdom.<br>andrew.pollard@paediatrics.ox.ac.uk
Co-Investigator	Prof Brian Angus<br>Professor of Infectious Diseases / Consultant Physician /Director<br>Oxford Centre for Clinical Tropical Medicine,<br>University of Oxford,<br>Nuffield Department of Medicine Research Building (NDRMB),<br>Old Road Campus, Headington, Oxford, OX3 7FZ, United Kingdom.<br>brian.angus@ndm.ox.ac.uk
DSMC Chair	Dr David Lalloo<br>Professor of Tropical Medicine<br>Liverpool School of Tropical Medicine<br>dlalloo@liv.ac.uk
Statistician	Merryn Voysey<br>Senior Trial Statistician<br>Primary Care Clinical Trials Unit<br>Nuffield Department of Primary Care Health Sciences,<br>University of Oxford,<br>23-38 Hythe Bridge Street, Oxford, OX1 2ET, United Kingdom.<br>merryn.voysey@phc.ox.ac.uk
Lead Research Site	Oxford Vaccine Group<br>Centre for Clinical Vaccinology & Tropical Medicine (CCVTM),<br>Churchill Hospital, Oxford OX3 7LE, United Kingdom.
Sponsor	The University of Oxford
Monitor	Clinical Trials and Research Governance<br>Joint Research Office, Block 60, Churchill Hospital, Old Road, Headington, Oxford OX3 7LE, United Kingdom

2. SYNOPSIS

Study Title	**Investigating the mechanisms and determinants of systemic and mucosal immunity to *Salmonella* Typhi and *Salmonella* Paratyphi A in naïve and previously exposed individuals – A challenge and rechallenge study.**	
Internal ref. no. / short title	**OVG 2014/01** / Investigating Enteric Fever	
Abbreviated study name	**PATCH** (Paratyphoid And Typhoid CHallenge)	
Study Design	Single-blinded, randomised, outpatient, ambulatory design, human infection study in two groups: naïve individuals and participants previously exposed to *Salmonella* Typhi or Paratyphi challenge.	
Study Participants	Healthy adult volunteers (aged 18 to 60) who have not been previously exposed to typhoidal salmonella or live Vi vaccine (Part A) and healthy adult volunteers who have previously participated in an OVG typhoid or paratyphoid challenge study (Part B).<br>A cohort of healthy volunteers not previously exposed to typhoidal salmonella or live Vi vaccine will be recruited to act as negative controls.	
Planned Sample Size (Number of participants enrolled)	40- 60 (Part A) + 20-100 (Part B)<br>3 – 10 (negative control group)	
Planned Study Period	Planned clinical study period from August 2014 to December 2016.<br>Expected completion of sample analysis by August 2019.	
	**Objectives**	**Endpoints**
Primary	Measure the attack rate after challenge with *S.* Typhi or *S.* Paratyphi A in naïve and previously challenged individuals.	Clinical and/or microbiological proven enteric fever infection.

Secondary	To describe the human clinical response to *S*. Typhi or *S*. Paratyphi A in antigen-naïve and previously challenged individuals.	The clinical response after challenge or re-challenge using:<br>• participant symptom profiles<br>• Laboratory parameters including inflammatory markers, blood count, liver function tests and microbiological culture results.
	To describe the characteristics of bacterial dynamics after challenge in naïve and previously exposed individuals, including onset and duration of bacteraemia, bacterial burden at diagnosis, bacterial burden in enteric fluid, and stool shedding.	Microbiological culture (qualitative and quantitative) and PCR to detect and characterise *S*. Typhi or *S*. Paratyphi A in blood, stool and enteric fluid.
	To determine the gut luminal mucosal response to challenge with *S*. Typhi and *S*. Paratyphi.	Mucosal inflammation as determined by:<br>• Macroscopic appearance of mucosal inflammation as seen on endoscopy and wireless video capsule endoscopy, as judged by experienced endoscopists.<br>• Inflammatory markers such as calprotectin and lactoferrin performed on stool and duodenal secretions.
	To describe the human immune response to challenge or re-challenge, including the innate,	Immunological laboratory assays including quantitative and functional assays of the humoral (e.g. ELISA, ELISpot, SBA and opsonophagocytosis assay), mucosal (secretory IgA

	humoral, cell-mediated and mucosal responses.	measurement), and cell-mediated (multi-chromatic flow cytometry, mass cytometry, cytokine measurement) immune response.
	Determine host genetic features influencing:<br>• clinical manifestations of challenge with typhi/paratyphi<br>• alteration of those responses through epigenetic changes<br>• and control of gene and protein expression.	Laboratory and high-throughput assays including:<br>• measurement of baseline genetic susceptibility factors by whole genome sequencing<br>• the transcriptional response to challenge and infection<br>• detection of translated proteins by mass spectrometry (CyTOF).
Exploratory	Exploratory immunology to investigate the innate, humoral, cell-mediated and mucosal responses to challenge with *S*. Typhi or *S*. Paratyphi A in naïve and previously challenged individuals.	Novel assays performed on peripheral blood, faeces,, saliva and mucosal biopsies, including assays for CD1 and MR1 restricted T cell responses and whole blood and PMBC killing assays.
	To investigate how the human microbiome influences and interacts with a challenge of *S*. Typhi or *S*. Paratyphi A	Samples of stool and enteric fluid to measure the constituent microbiological flora by assays such as pyrosequencing and related metagenomic studies.
	Investigate new molecular techniques for detection of *S*. Typhi and/or S. Paratyphi in clinical samples.	Use of novel methodologies to prepare bacterial DNA/RNA and development of sensitive quantitative and qualitative PCR assays for blood, stool and duodenal secretions.

	To investigate recruitment methods and reasons for volunteer exclusions from typhoid and paratyphoid challenge models.	Analysis of recruitment numbers, including:<br>• Number of positive and negative responses to different recruitment techniques;<br>• Number of volunteers excluded prior to attending screening visits and reasons for exclusion;<br>• Number of volunteers attending for screening visits and reasons for exclusion.

ABBREVIATIONS

AE	Adverse Event
AESI	Adverse Event of Special Interest
ALT	Alanine aminotransferase
APR	Annual Progress Report
AST	Asparate aminotransferase
AR	Adverse Reaction
CCVTM	Centre for Clinical Vaccinology and Tropical Medicine
CI	Chief Investigator
CFU	Colony Forming Unit
CRF	Case Report Form
eCRF	(electronic) Case Report Form
CTRG	Clinical Trials & Research Governance, University of Oxford
DNA	Deoxyribonucleic Acid
DSUR	Development Safety Update Report
DSMC	Data Safety Monitoring Committee
EDTA	Ethylenediaminetetraacetic acid
ESS	Enteric String Sampling
ELISA	Enzyme-Linked Immunosorbent Assay
ELISpot	Enzyme-Linked Immunosorbent Spot
GCP	Good Clinical Practice

GMP	Good Manufacturing Practice
GP	General Practitioner
ICF	Informed Consent Form
ICH	International Conference of Harmonisation
ISF	Investigator Site File
MHRA	Medicines and Healthcare products Regulatory Agency
MDR	Multi-Drug Resistant
NHS	National Health Service
NRES	National Research Ethics Service
OUH	Oxford University Hospitals
OVC	Oxford Vaccine Centre
OVG	Oxford Vaccine Group
PBMC	Peripheral Blood Mononuclear Cell
PE	Protective Effect
PCR	Polymerase Chain Reaction
PI	Principal Investigator
PIL	Participant/ Patient Information Leaflet
PRN	Pro re nata or ‘as needed’
R&D	NHS Trust R&D Department
REC	Research Ethics Committee
RNA	Ribonucleic Acid

SAE	Serious Adverse Event
SAR	Serious Adverse Reaction
SBA	Serum Bactericidal Assay
SOP	Standard Operating Procedure
SUSAR	Suspected Unexpected Serious Adverse Reaction
TMF	Trial Master File
TOPS	The Over volunteering Prevention System (http://www.tops.org.uk)
WIMM	Weatherall Institute of Molecular Medicine
WBC	White Blood cell Count
WCE	Wireless Video Capsule Endoscopy

3. BACKGROUND AND RATIONALE

Enteric fever is a major cause of morbidity worldwide, particularly among young and school-age children in resource-limited countries[1]. Enteric fever is caused by faecal-oral transmission and subsequent infection by Gram negative bacteria of the subspecies *Salmonella enterica enterica*, in particular serovars Typhi and Paratyphi A. These serovars only infect humans and do not survive for long in the environment. While ultimate eradication of enteric fever may be achieved through improvement of clean water and sanitation infrastructure, these improvements are costly and require coordinated political will. In the interim, vaccination of those at highest risk is likely to have the greatest impact on disease prevalence[2].

Current licenced vaccines for the prevention of enteric fever deliver, at best, moderate protection against *S*. Typhi only, and are not licensed for use in infants. It is recognised that *S*. Paratyphi is responsible for an increasing proportion of enteric fever cases and that infants and young children bear the majority of the disease burden. Key additional features problematic to the control of enteric fever include emerging recognition of a previously underestimated disease burden in sub-Saharan Africa and the intercontinental spread of multi-drug resistant strains[3]. The development of efficacious vaccines against this human-restricted infection which disproportionately affects the most impoverished sections of society should therefore be a major priority for the global health community.

Advancement in the development of novel vaccines and diagnostics for enteric fever is held up by deficits in understanding in a number of areas. Specifically, the nature of the protective immune response resulting from infection and/or vaccination including the extent of involvement of mucosal immunity and the degree to which exposure to different serovars influences cross-protection is not known. In addition, vital potential alternative vaccine targets and correlates of protection are also not available which would greatly facilitate vaccine development and field-assessment of efficacy. As the bacteria causing enteric fever are human-restricted, the lack of suitable small animal models for *S.* Typhi or Paratyphi infection has impeded scientific progress in these areas.

Based on historical studies performed at the University of Maryland (1952-1974), a new human typhoid challenge model has been developed and successfully implemented at the Oxford Vaccine Group (Centre for Clinical Vaccinology and Tropical Medicine). The aim of this model is to both further our understanding of the immunobiology of infection and to subsequently measure the protective efficacy of a new oral vaccine candidate[4]. Based on the success of these studies, development of a human model using *Salmonella* Paratyphi A challenge is now underway. Both models have provided unique opportunities to investigate a human-restricted pathogen in a host-relevant model in the context of a strictly monitored experimental setting. They have provided major advances in understanding of the host-pathogen interaction including the mechanisms of organism pathogenesis and the evolution of the infection-derived immunological response.

In this current study, we aim to explore the precise nature and protective mechanism of these immune responses further. The overall objective of OVG2014/01 is to investigate the human response to challenge with *S.* Typhi or *S.* Paratyphi A in participants who have been challenged in one of the previously performed studies and compare this with the response of a group of healthy antigen-naïve participants.

Pathogenesis

Infection by *Salmonella enterica* serovars Typhi, Paratyphi A and C can cause systemic illness after ingestion from a contaminated food- or water-source. *S.* Paratyphi B generally causes more of a gastrointestinal-type illness whereas *S.* Paratyphi C may cause systemic illness but is rarely reported[5]. In the following discussion **'*S.* Paratyphi' refers to *Salmonella enterica* subspecies *enterica* serovar Paratyphi A only**.

S. Typhi and *S*. Paratyphi cause clinically indistinguishable infections characterised as 'enteric fever'; previously it was thought that paratyphoid had a more mild disease course, however more recent data suggests this is not the case, and may be associated with at least similar rates of severe illness and complications if not managed appropriately[6,7].

Bacteria causing enteric fever colonize the intestine by attaching to the small intestinal mucosa before invading the epithelial lining. Flagella enable *Salmonella* species to travel to the epithelial lining with fimbriae or pili assisting initial attachment. However, just how the bacteria attach and invade needs to be further elucidated. For instance, *Salmonella* pathogenicity islands (SPIs) harbour genes that are crucial for invasion of gut epithelial cells and initiation of an inflammatory response; however other pathways that cause systemic infection without disrupting the gut epithelial barrier exist in *Salmonella* species, such as the pathway via CD-18 expressing phagocytic cells. It is thought that specialized epithelial cells, such as M cells that overlie lymphoid nodules in the terminal ileum (Peyer's patches), are the site of internalization of *S*. Typhi[8]. Subsequent dissemination of bacteria contained within macrophages through bloodstream dispersal is thought to cause a 'silent' primary bacteraemia. This results in dissemination of *Salmonella* bacteria to the reticuloendothelial system, including the liver, spleen, lymph and bone marrow. After a latent period (corresponding to the clinical incubation period) of approximately one week (range 3-60 days), infected patients develop secondary bacteraemia accompanied by symptoms of fever, malaise, headache, abdominal pain and diarrhoea or constipation[5,9]. If undiagnosed or insufficiently treated, patients with prolonged infection (> 3weeks in duration) may develop complications including gastrointestinal perforation or haemorrhage.

Although clinically indistinguishable *S*. Paratyphi differs structurally from *S*. Typhi, notably in the absence of the Vi polysaccharide capsule. This is encoded by the viaB operon, part of the SPI-7 gene[10]. Individuals are therefore not protected from *S*. Paratyphi after vaccination with the Vi-polysaccharide vaccine. Despite the lack of Vi, which is thought to be responsible for many of the unique features of human typhoid infection, *S*.

Paratyphi is equally capable of causing systemic infection in exposed persons. The reasons for apparent similarities in human infection despite different pathogenic mechanisms are unknown, as is the public health significance of Vi-negative *Salmonella* Typhi strains[11,12].

The host immune response to infection with S. Typhi and S. Paratyphi is also of interest in the way it differs from other gastrointestinal pathogens. Evasion of host immune responses, possibly by down-regulation of innate host response is evidenced by a lack of neutrophil infiltrate in the mucosal epithelium. This correlates with certain clinical observations in enteric fever, particularly a lack of septic shock despite bacteraemia[13]. Individuals in endemic areas may be infected repeatedly with *S*. Typhi or *S*. Paratyphi strains and it is probable that repeated exposures contribute to protection as well [14].

Epidemiology

Global burden of disease estimates in 2010 suggest enteric fever is the 35th leading cause of 'Life Years Lost' worldwide [15]. The peak incidence of reported deaths is in 5-14 year olds in South East Asia, and in this age group enteric fever is the 8th most common cause of mortality worldwide. It is increasingly recognised that infection in the under 5's contributes significantly to the proportion of disease[16,17], which highlights the importance of developing vaccines suitable for this age-group in particular[18].

Reviews estimate between 21 and 26 million cases of enteric fever occur globally each year, although these estimates likely reflect widespread under-diagnosis[19,20]. Enteric fever presents as a non-specific febrile condition in endemic areas, where the range of differential diagnoses may be broad and include malaria, typhus and rickettsial infections. Diagnosis of enteric fever, both in the individual and at the population level is severely limited by the lack of sensitive and specific diagnostic tests[21]. Additional factors leading to underreporting include the widespread injudicious use of empirical pre-hospital antibiotics and the neglected and impoverished nature of the sections of society most affected[22,23].

In terms of geographical distribution, South-central, South-east Asia and sub-Saharan Africa exhibit the highest prevalence of documented infection[20]. However, the extent of salmonellosis due *S.* Typhi is not yet fully understood in Africa and rates of infection are likely higher than those reported[24,25].

Traditionally, the majority of cases of enteric fever were thought to be caused by *S*. Typhi, however, there is evidence that, particularly in Asia, *S*. Paratyphi A may actually be responsible for a considerable proportion of cases reported[26,27]. A recent review describes rates of *S*. Paratyphi A of between 10% and 55% in differing regions of India and as high as 90% in regions of China[27]. In some areas, for example Sri Lanka, paratyphoid has completely replaced typhoid, suggesting that the same ecological niche is exploited by both serovars[28]. Paratyphoid is also a cause for concern in travellers, in particular those visiting friends and relatives, as the commonly used typhoid Vi polysaccharide vaccine has no protective effect against paratyphoid. Large studies estimate that *S*. Paratyphi infection causes approximately one third of all enteric fever in returning

travellers[29,30]. These data suggest that the disease burden attributable to enteric fever cannot be fully addressed unless equal effort is paid to both *S*. Typhi and *S*. Paratyphi prevention.

Transmission

Humans are the only reservoir of typhoid and paratyphoid infection, with the gallbladder thought to be the predominant site for long-term carriage[31,32]. Spread between individuals occurs through faecal-oral transmission, mainly via contaminated food or water. In areas with adequate sanitation (i.e. flushing latrines), person-to-person transmission is rare[33]. A retrospective case note review studied 645 contacts of 329 cases of enteric fever in London; all had travelled to endemic regions, and only 10 additional cases were identified, all of whom had travelled abroad with their contact and were presumably infected by the same source rather than person-to-person contact[34].

Treatment

Antibiotic treatment of enteric fever in endemic countries is often empiric and provided by untrained practitioners. If professional healthcare advice is sought, clinical decision-making is hampered by a lack of sensitive and specific diagnostic tests. Antibiotic susceptibility test requires organism culture: in many endemic areas serological methods are used for diagnosis and therefore subsequent testing is not possible. Even in advanced healthcare settings, the interpretation of antimicrobial susceptibility profiles is complex and frequently updated[35]. Chloramphenicol, trimethoprim-sulfamethoxazole, ampicillin and ciprofloxacin are the most widely used antibiotics in most settings, however reduced susceptibility to all of these antibiotics has been recognised[36]. Use of ineffective antibiotics has been associated with adverse clinical outcomes for typhoid and the same is likely to be the case for paratyphoid[37]. Although the optimal treatment regime for enteric fever is still under investigation, fluoroquinolones (for example, ciprofloxacin) remain the preferred antibiotic class for susceptible strains and for clearance of long-term carriage, as they are concentrated in the biliary tract and have a high volume of distribution[9,38]. Azithromycin is another commonly used treatment for enteric fever. It is capable of achieving excellent intracellular concentrations, and its use has been increasing as a result of rising fluoroquinolone resistance [39,40,41,42].

Trends in antimicrobial resistance are difficult to generalise as they appear to vary greatly within region and with time. Multi-drug resistance (MDR) is defined as resistance to ampicillin, chloramphenicol and trimethoprim-sulfamethoxazole. Recent reviews estimating prevalence of MDR enteric fever infections range from 0% (in China) to 65% (in Pakistan) of infections[42]. The other major pattern of resistance which is commonly found is nalidixic acid resistance which is a key laboratory marker of impending decreased susceptibility to fluoroquinolone antibiotics. Sharply increasing rates of quinolone resistance have been recorded in many areas including Nepal. From 2008 to 2011 more than 10% of isolates of *S*. Typhi and *S*. Paratyphi were fully resistant compared to less than 4% between 1998 and 2002[43].

Vaccines

Whole-cell vaccines to prevent typhoid have been in use for more than 100 years although the newer, less reactogenic oral Ty21a vaccine has only been in more widespread use since the late 1960's[44]. Use of available licensed typhoid vaccines (Vi polysaccharide or Ty21a) in areas and age-groups at high-risk of infection is advocated by the WHO[45]. Implementation has been limited in practice for myriad reasons, including limited financial resources, lack of political will and advocacy and the variable vaccine efficacy results obtained in historic field-trials performed in endemic regions. Vaccine implementation has become further frustrated by the cessation of Ty21a manufacture in Europe and interruptions to supply of Vi polysaccharide vaccine[46].

There are no vaccines currently available for the prevention of *S*. Paratyphi A infection although there is limited *in vitro* data suggesting cross-reactive immunogenicity by Ty21a to *S*. Paratyphi B [47,48]. Several vaccine candidates are in Phase I and II of development including a conjugate vaccine targeted at the O:2 lipopolysaccharide component of *S*. Paratyphi (US National Institute of Health[49], Novartis Institute for Global Health[50]) and several live attenuated vaccine candidates (University of Maryland[51]).

As with typhoid, the development of a vaccine to prevent paratyphoid infection is severely hampered by lack of a suitable small animal model and detailed knowledge regarding likely immunological correlates for protection. Additional complexities in paratyphoid vaccine development include the lack of an available licensed vaccine to act as comparator, or with which to search for or confirm putative correlates, or from which to perform bridging studies towards more rapid licensure.

Aim of this project

This project aims to advance understanding of host-bacteria interactions from the time of initial exposure through to the development of infection in the context of previous exposure to the specific pathogen of interest. In comparing groups of individuals who have been previously exposed to the pathogen in a known, well-defined, challenge setting and infection/vaccine-naïve individuals, we will be able to directly assess the contribution of infection-derived immunity/exposure in the prevention (or otherwise) of subsequent pathogen interactions. Better understanding of the immune mechanisms which determine susceptibility to disease will inform and expedite much needed vaccine development for both S. Typhi and S. Paratyphi serovars, and provide vital data supporting the likely impact that vaccine introduction may have in an endemic setting.

Background to this project

A live challenge model of typhoid infection was first developed at the University of Maryland in the 1960's[52,53]. Studies performed involved almost 2000 participants and described developing humoral immunity after challenge with *S*. Typhi in vaccinated and unvaccinated volunteers[54]. Studies in Maryland were terminated due

to the more widespread concern regarding the use of prisoners and other vulnerable groups in clinical research[55].

A human typhoid challenge model was developed at the University of Oxford by the Oxford Vaccine Group between 2009 and 2012 (REC no. 10/H0604/53, OVG 2009/10; REC no. 11/SC/0302, OVG 2011/2). This new model was novel in using sodium bicarbonate as a gastric acid buffer, allowing a consistent lower challenge dose to be used and resulting in a smoother pattern of clinical infection. The model was also set up to be performed using outpatient community volunteers, which demonstrated safety and participant approval[4].

In OVG 2009/10, 41 healthy volunteers were challenged with escalating dose levels of *S.* Typhi suspended in a bicarbonate buffer solution and preceded by ingestion of a sodium bicarbonate solution. Participants were followed daily until diagnosis or day 14 at which point they were treated with eradicative antibiotics. Participants developing infection tolerated the clinical course well, and all participant symptoms responded rapidly to antibiotic treatment. Four participants met predetermined criteria for severe infection (2 recorded temperatures exceeding 40°C and 2 had >grade 3 laboratory abnormalities), however none required hospital admission, intravenous antibiotics or fluids. Participants were promptly treated on confirmation of typhoid diagnosis and no instances of stool shedding after treatment or transmission to secondary contacts were detected[4].

The model developed was used in the subsequent clinical trial OVG 2011/02, to assess the directly protective efficacy of a novel single dose oral typhoid vaccine (M01ZH09). Additionally, use of a human typhoid model was validated for vaccine assessment by demonstration of a consistent attack rate of 66% in the placebo vaccinated group (an attack rate of 65% had been demonstrated in the initial dose-finding study), and by demonstration of a protective effect (PE) by 3-doses of the licensed Ty21a vaccine (PE=35%). The study was performed safely, and challenge was well tolerated. The most significant study related SAE was related to use of ciprofloxacin, rather than vaccination or illness related to challenge; a participant developed symptoms of acute depression on a background of low mood, which resolved with time without pharmacological treatment or hospital admission.

Significant advances have been made in performing these human typhoid challenge studies, including first confirmation of the primary bacteraemia, identification of novel clinical and laboratory biomarkers in addition to major progression in understanding the host response to infection and microbiological dynamics. This information has and will directly inform vaccine design and development, transmission modelling and potential public health intervention strategies[4,56].

In the pathway to the development of a novel paratyphoid conjugate vaccine, Novartis Vaccines for Global Health has manufactured a *S.* Paratyphi strain, NVGH308, to GMP standard by GenIbet BioPharmaceuticals (Portugal). This strain originated from a clinical case of paratyphoid infection that occurred in Nepal.

Availability of a GMP standard challenge strain provided the opportunity to develop a *S.* Paratyphi human challenge model, using many of the previous design features used in, and the experience gained from, performing typhoid challenge studies. This paratyphoid challenge study is currently in development and will recruit between 20 and 80 participants, with the primary outcome of determining the dose (in colony forming units) of *Salmonella* Paratyphi A, challenge strain NVGH308, needed to produce a 60% to 75% attack rate in healthy volunteers.

Selection of the challenge agents

Salmonella Typhi (Quailes strain) was used extensively for human challenge studies in the 1960s/70s and has been provided by the University of Maryland to establish a GMP master cell bank in Oxford. Full antibiotic sensitivity of the strain stored in the master cell bank has been demonstrated and further characterization work including genome-sequencing has been completed at the Wellcome Trust Sanger Institute, Cambridge UK. The Quailes strain has been used to safely challenge 132 participants in Oxford since 2009.

The *S.* Paratyphi strain, NVGH308, is supplied to the Oxford Vaccine Group by Novartis Vaccines for Global Health. Further details regarding strain manufacture and preparation are given in Section 9, below.

4. OBJECTIVES AND ENDPOINTS

**Primary Objective**	**Primary Endpoint**
Measure the attack rate after challenge with *S*. Typhi or *S*. Paratyphi A in naïve and previously challenged individuals.	Clinical and/or microbiological proven enteric fever infection.
**Secondary Objectives**	**Secondary Endpoints**
To describe the human clinical response to *S*. Typhi or *S*. Paratyphi A in antigen-naïve and previously challenged individuals.	The clinical response after challenge or re-challenge using:<br>• participant symptom profiles<br>• laboratory parameters including inflammatory markers, blood count, liver function tests and microbiological culture results.

To describe the characteristics of bacterial dynamics after challenge in naïve and previously exposed individuals, including onset and duration of bacteraemia, bacterial burden at diagnosis, bacterial burden in enteric fluid, and stool shedding.	Microbiological culture (qualitative and quantitative) and PCR to detect and characterise *S*. Typhi or *S*. Paratyphi A in blood, stool and enteric fluid.
To determine the gut luminal mucosal response to challenge with *S*. Typhi and *S*. Paratyphi.	Mucosal inflammation as determined by:<br>• Macroscopic appearance of mucosal inflammation as seen on endoscopy and wireless video capsule endoscopy, as judged by experienced endoscopists.<br>• Inflammatory markers such as calprotectin and lactoferrin performed on stool and duodenal secretions
To describe the human immune response to challenge or re-challenge, including the innate, humoral, cell-mediated and mucosal responses.	Immunological laboratory assays including quantitative and functional assays of the humoral (e.g. ELISA, ELISpot, SBA and opsonophagocytosis assay), mucosal (secretory IgA measurement), and cell-mediated (multi-chromatic flow cytometry, mass cytometry, cytokine measurement) immune response.
Determine host genetic features influencing:<br>• clinical manifestations of challenge with typhi/paratyphi<br>• alteration of those responses through epigenetic changes<br>• and control of gene and protein expression.	Laboratory and high-throughput assays including:<br>• measurement of baseline genetic susceptibility factors by whole genome sequencing<br>• the transcriptional response to challenge and infection<br>• detection of translated proteins by mass spectrometry (CyTOF).
**Exploratory Objectives**	**Exploratory Endpoints**

Exploratory immunology to investigate the innate, humoral, cell-mediated and mucosal responses to challenge with *S*. Typhi or *S*. Paratyphi A in naïve and previously challenged individuals.	Novel assays performed on peripheral blood, faeces, saliva and mucosal biopsies, including assays for CD1 and MR1 restricted T cell responses and whole blood and PMBC killing assays.
To investigate how the human microbiome influences and interacts with a challenge of *S*. Typhi or *S*. Paratyphi A	Samples of stool and enteric fluid to measure the constituent microbiological flora by assays such as pyrosequencing and related metagenomic studies.
Investigate new molecular techniques for detection of *S*. Typhi and/or S. Paratyphi in clinical samples.	Use of novel methodologies to prepare bacterial DNA/RNA and development of sensitive quantitative and qualitative PCR assays for blood, stool and duodenal secretions.
To investigate recruitment methods and reasons for volunteer exclusions from typhoid and paratyphoid challenge models.	Analysis of recruitment numbers, including:<br>• Number of positive and negative responses to different recruitment techniques;<br>• Number of volunteers excluded prior to attending screening visits and reasons for exclusion;<br>• Number of volunteers attending for screening visits and reasons for exclusion.

5. STUDY DESIGN

5.1. Part A and Part B

This is a single-blinded, randomised, human infection study using *S.* Typhi or *S.* Paratyphi A challenge of ambulatory, out-patient healthy community adult volunteers. We will enrol a cohort of 40 to 60 volunteers not previously exposed to *S*. Typhi or *S*. Paratyphi into **Part A.**

Part B will involve 20 to 100 volunteers recruited from participants of previous typhoid (n=≥242; OVG2009/10, OVG2011/02, OVG 2014/08 or other future typhoid challenge studies if applicable) and paratyphoid (n>=20;

OVG2013/07 and other future paratyphoid challenge studies if applicable) challenge studies, including re-recruitment from the Part A cohort of this study if needed. Part A and Part B cohorts will be randomised on enrolment to be challenged with either *S*. Typhi or *S*. Paratyphi. Continuous participant safety monitoring will occur throughout (by use of daily clinical review and e-diary cards).

5.2. Negative control group

A preliminary cohort of three volunteers not previously exposed to *S*. Typhi or *S*. Paratyphi will be recruited to act as negative controls prior to enrolment of volunteers to Part A. The same inclusion and exclusion criteria for Part A and Part B applies, minus the need to exclude volunteers on the basis of having 'high risk' close contacts (see section 7.2) or working in commercial food handling. While this group will not be blinded, they will be required to ingest a bicarbonate buffer, complete a course of antibiotics and ideally have two endoscopies with mucosal biopsies. From these three to ten volunteers the same immunological assays as samples from Part A and B will be performed.

The purpose of negative controls is to determine if the combination of repeated blood sampling, mucosal biopsies and antibiotics have an effect on the systemic and/or mucosal immunological profile. Antibiotics are known to change the gut microbiota which in itself can impact on immune function, ranging from induction of antibody and T cell differentiation, to reduction of isolated lymphoid follicles[57].

Quantitative and functional assays of the humoral (such as ELISA, ELISpot, SBA and opsonophagocytosis), mucosal (secretory IgA measurement), and cell-mediated (flow cytometry, cytokine measurement) immune response will be performed. If a positive immunological signal is present then a further seven volunteers, to a maximum of ten in this negative control cohort, will be recruited after Part A enrolment is complete.

For simplicity, description of the study procedures and visits for the negative control group are detailed in Appendix A.

5.3. Study overview

An overview of the study is summarised in **Figure 1**.

For clarity it will be necessary in places to address both parts of the study as a whole (e.g. study overview and data interpretation) and in other places it will be easier to explain each part separately (e.g. recruitment). Where the protocol refers specifically to an individual study part, this will be explicitly stated. The majority of information referring to the negative control cohort will be in Appendix A.

An individual participating in Part A of this study can go on to participate in a re-challenge (Part B). If this were the case we would require the participant to undergo screening and consent again to ensure eligibility with a period of 12 months between challenges.

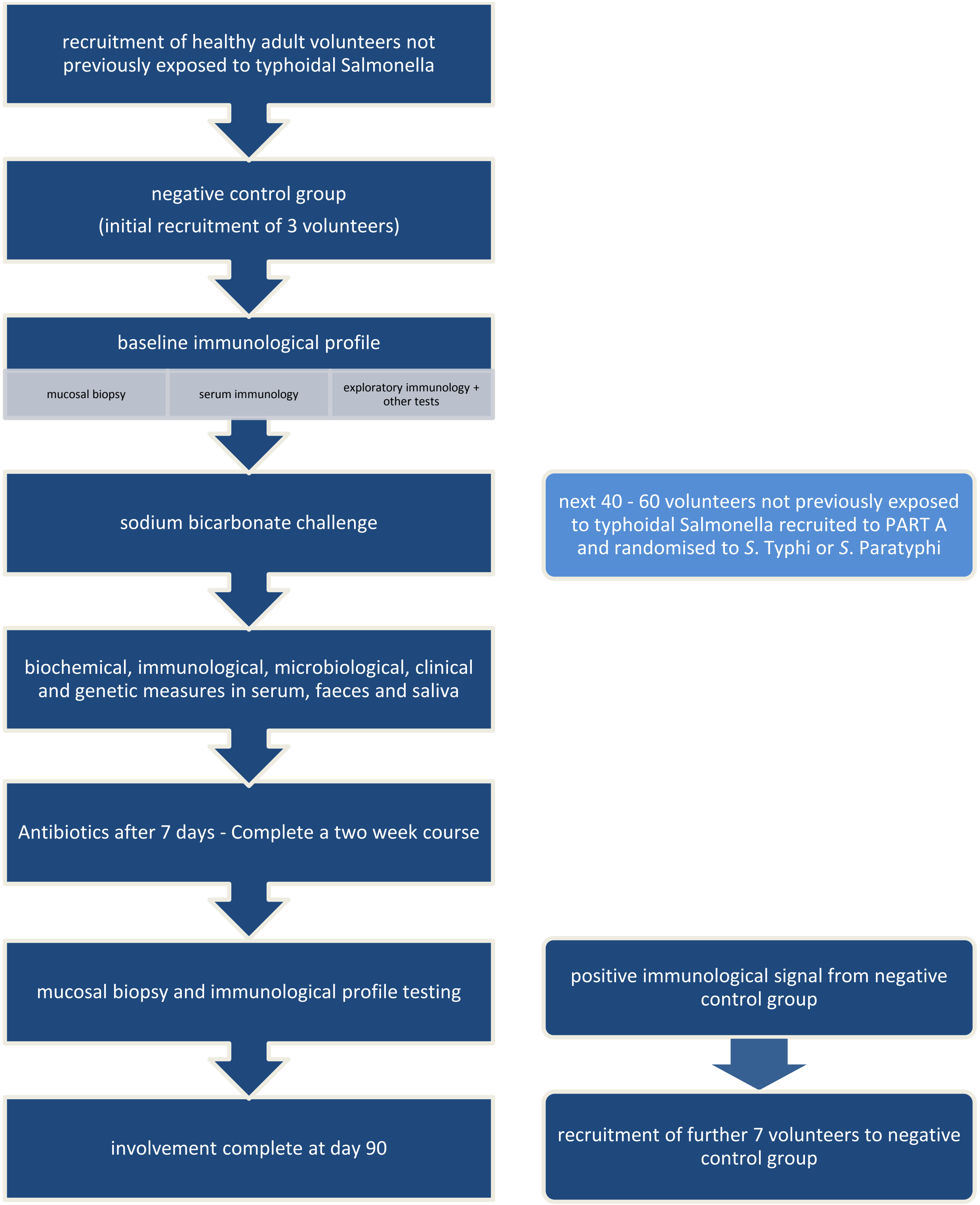

Figure 1a. Overview of Study – negative control group

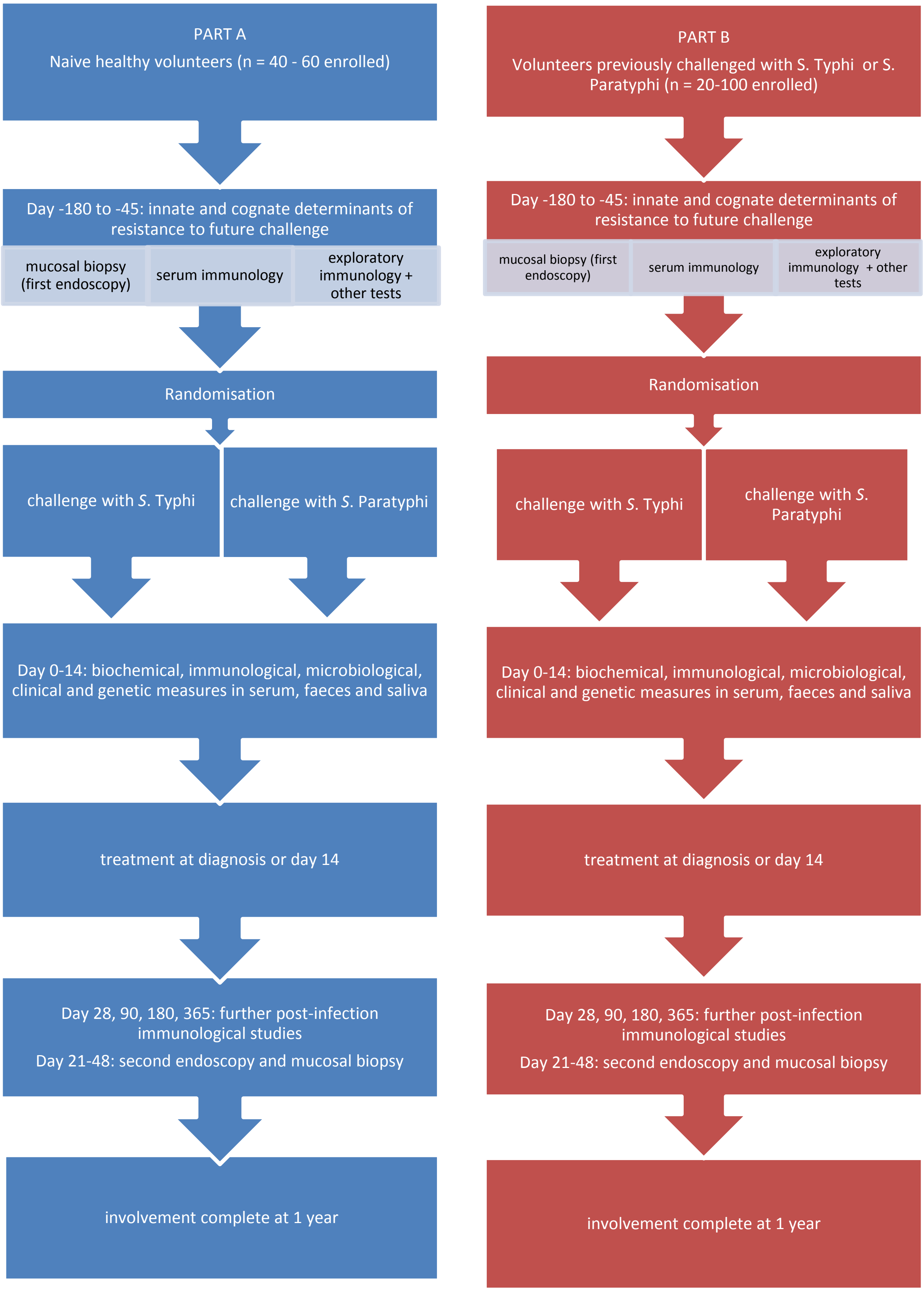

Figure 1b. Overview of Study – Part A and B

6. PARTICIPANT IDENTIFICATION

6.1. Study Participants

Study participants will be between the ages of 18 and 60 inclusive on day of challenge. They will be screened to ensure they are healthy and willing to co-operate with study procedures.

6.2. Inclusion Criteria

Participants must satisfy all of the following criteria to be considered eligible for the study:

- Agree to give informed consent for participation in the study.
- Aged between 18 and 60 years inclusive at time of challenge.
- In good health as determined by medical history, physical examination and clinical judgment of the investigators.
- Agree (in the Investigator's opinion) to comply with all study requirements, including capacity to adhere to good personal hygiene and infection control precautions.
- Agree to allow his or her General Practitioner (and/or Consultant if appropriate), to be notified of participation in the study.
- Agree to allow Public Health England to be informed of their participation in the study[*].
- Agree to give his or her close household contacts written information informing them of the participants' involvement in the study and offering them voluntary screening for *S.* Typhi or *S.* Paratyphi carriage[*].
- Agree to have 24-hour contact with study staff during the four weeks post challenge and are able to ensure that they are contactable by mobile phone for the duration of the challenge period until antibiotic completion[*].
- Have internet access to allow completion of the e-diary and real-time safety monitoring.
- Agree to avoid antipyretic/anti-inflammatory treatment until advised by a study doctor or until at least 14 days after challenge.
- Willing to undergo endoscopy and biopsy.

---

[*] This inclusion criteria does not apply to the negative control group

- Agree to provide their National Insurance/Passport number for the purposes of TOPS registration and bank details for the purpose of reimbursement for the duration of the their participation. Bank details will be stored electronically for the duration of the study.

6.3. Additional Inclusion Criteria for Part B

Participants must satisfy all of the above inclusion criteria as well as the following to be considered eligible for the study:

- Have been challenged with *S.* Typhi or *S.* Paratyphi within the last five years during which they were seen at least up to and including the Day 28 visit (completion of challenge and antibiotic treatment).

6.4. Exclusion Criteria

The participant will not be enrolled if any of the following apply:

- History of significant organ/system disease that could interfere with study conduct or completion. Including, for example, but not restricted to:
    - Cardiovascular disease
    - Respiratory disease
    - Haematological disease
    - Endocrine disorders
    - Renal or bladder disease, including history of renal calculi
    - Biliary tract disease, including biliary colic, asymptomatic gallstones, previous cholecystectomy, or abnormal ultrasound of the gallbladder
    - Gastro-intestinal disease including requirement for antacids, H_2-receptor antagonists, proton pump inhibitors or laxatives
    - Neurological disease
    - Metabolic disease
    - Autoimmune disease
    - Psychiatric illness requiring hospitalisation or known or suspected drug and/or alcohol misuse (alcohol misuse defined as an intake exceeding 42 units per week)
    - Infectious disease

- Have any known or suspected impairment of immune function, alteration of immune function, or prior immune exposure that may alter immune function resulting from, for example:
  - Congenital or acquired immunodeficiency, including IgA deficiency
  - Human Immunodeficiency Virus infection
  - Receipt of immunosuppressive therapy such as anti-cancer chemotherapy or radiation therapy within the preceding 12 months or long-term systemic corticosteroid therapy
  - Receipt of immunoglobulin or any blood product transfusion within three months of study start
  - History of cancer (except squamous cell or basal cell carcinoma of the skin and cervical carcinoma in situ)
- Moderate or severe depression or anxiety as classified by the Hospital Anxiety and Depression Score at screening or challenge that is deemed clinically significant by the study investigators[†].
- Weight 50kg or less[‡].
- Presence of implants or prosthesis[§].
- Have previously received any typhoid vaccine[**]
- Have previously been diagnosed with laboratory confirmed typhoid or paratyphoid infection, or been given a diagnosis compatible with enteric fever**
- Have participated in previous typhoid or paratyphoid challenge studies with ingestion of challenge agent**
- Any contraindication to elective upper GI endoscopy (e.g. history of oesophageal perforation, recent myocardial infarction, anticoagulation, pharyngeal diverticulum, head and neck surgery).
- More than one non-study related upper GI endoscopy within the last year.
- Anyone taking long-term medication (e.g. analgesia, anti-inflammatories or antibiotics) that may affect symptom reporting or interpretation of the study results.

[†] If elevated scores are due to temporary significant life events, the questionnaire may be repeated after resolution of the event with a view to inclusion if normal.

[‡] Or a body mass index (BMI) that, in the opinion of the study team, may adversely impair the interpretation of the study results or affect the safe performance of any study procedure.

[§] This exclusion criteria does not apply to the negative control group
[**] For Part A only.

- Contra-indication to taking azithromycin, ciprofloxacin, trimethoprim/sulfamethoxazole and/or beta lactam antibiotics.
- Female participants who are pregnant or lactating.
- Female participants who are unwilling to ensure that they or their partner use effective contraception one month prior to challenge and continue to do so until two negative stool samples, a minimum of three weeks after completion of antibiotic treatment, have been obtained (see section 9.3)[§].
- Full-time, part-time or voluntary occupations involving:
    - Clinical or social work with direct contact with young children (defined as those attending pre-school groups or nursery or aged under 2 years)[††], or
    - Clinical or social work with direct contact with highly susceptible patients or persons in whom typhoid or paratyphoid infection would have particularly serious consequences e.g. the elderly or infirm (unless willing to avoid work until demonstrated not to be infected with *S*. Typhi or *S*. Paratyphi in accordance with guidance from Public Health England and willing to allow us to inform their employer) [**].
- Full time, part time or voluntary occupations involving:
    - Commercial food handling (involving preparing or serving unwrapped foods not subjected to further heating) [*].
- Close household contact with:
    - Young children (defined as those attending pre-school groups, nursery or those aged less than 2 years) [*]
    - Individual(s) who is (are) immunocompromised[*].
- Scheduled elective surgery or other procedures requiring general anaesthesia during the study period.
- Participants who have participated in another research study involving an investigational product that might affect risk of typhoid or paratyphoid infection or compromise the integrity of the study

[††] This exclusion criteria does not apply to the negative control group as will not be infectious.

within the 30 days prior to enrolment (e.g. significant volumes of blood already taken in previous study)[‡‡].

- Detection of any significantly abnormal results from screening investigations (at the clinical discretion of the study investigators).
- Inability to comply with any of the study requirements (at the discretion of the study investigators and the participants General Practitioner).
- Any other social, psychological or health issues which, in the opinion of the study investigator, may:
    - Put the participants or their contacts at risk because of participation in the study, or
    - Adversely affect the interpretation of the primary endpoint data, or
    - Impair the participant's ability to participate in the study.
- Having been resident in an enteric fever endemic country for six months or more.
- Currently on the study delegation log (Other exclusions for relatives of study team will be done at investigators discretion).

6.4.1. Temporary exclusion criteria at challenge (Both Part A and B)

Participants will be temporarily excluded from challenge if presenting at the challenge visit with the following:

- Significant acute or acute-on-chronic infection within the previous seven days or have experienced fever (>37.5°C) or subjective febrile symptoms within the previous three days.
- History of any antibiotic therapy during the previous 14 days.
- Any systemic corticosteroid (or equivalent) treatment in the previous 14 days, or for more than seven consecutive days within the past three months.
- Received any vaccine within the last four weeks.
- Recent significant blood donation (e.g., to the National Blood Service).

7. STUDY PROCEDURES

[‡‡]As assessed by both participant questioning and registration with The Over-volunteering Prevention System (TOPS) database.

Existing OVG and OVC Standard Operating Procedures (SOPs) govern the safe and accurate conduct of all study procedures, in addition to a study-specific Clinical Study Plan.

7.1. Part A and Part B

Part A: A cohort of 40 to 60 volunteers will be recruited and then randomly allocated 1:1 (20-30 in each group) to be challenged with 1-5 x 10^4CFU of *S.* Typhi or 1-5 x 10^3CFU *S.* Paratyphi. The dose of *S.* Typhi and S.Paratyphi has been determined in previous challenge studies to produce an attack rate of 60-75% in naïve individuals when ingested with a bicarbonate buffer

Part B: Participants from previous challenge studies in OVG will be approached, aiming to recruit a cohort of between 20 and 100. They will then be randomised 1:1 and challenged with *S*. Typhi or *S*. Paratyphi as described above.

Prior to challenge, participants (from both parts A and B) will undergo duodenal mucosal biopsy, which will be repeated after completion of two weeks antibiotic treatment. After challenge, participants will be reviewed daily for at least 14 days by study investigators. Samples of blood, stool and, saliva willbe collected. Participants diagnosed with enteric fever (defined in **Table 3**) will be treated immediately with antibiotics and samples will be taken as per the enteric fever diagnosis section of **Table 2**. The participants who do not meet criteria for enteric fever will be treated with antibiotics on Day 14.

A proportion of participants from Part A and B will also have Enteric String Sampling (ESS) and/or Wireless Video Capsule Endoscopies (WCE) performed. This will be offered as an optional additional test and offered to interested participants until the quota has been filled for each group (see Section 8.7).

The study procedures at each time point are outlined in **Table 1**. The study samples collection at each time point are described in **Table 2**.

See **Appendix A** for **negative control group** study procedures each time point.

Table 1. Study procedures and timepoints (Part A and B)

	Screening	Pre-challenge visits			Intensive Challenge Period (Day 0 – day 14)																	Go to follow-up visit section of table
Visit Number	screening visit (V0)	endoscopy V1	Pre-challenge V2		V03	V04	V05	V06	V07	V08	V09	V10	V11	V12	V13	V14	V15	V16	V17	V18		
Visit Time	within 120 days	--180 to -45 days	-7 days		D0	D0+ 12	D1	D2	D3	D4	D5	D6	D7	D8	D9	D10	D11	D12	D13	D14		
Consent & consent questionnaire	x																					
Continuing /procedure consent		X	x		x				x													
Randomisation		x																				
Confirm 24 hr contact details	x		x																			
Medical history	x																					
Interim medical history					x	x	x	x	x	x	x	x	x	x	x	x	x	x	x	x		
Physical examination[1]	x				x																	
Vital signs	x	x	x		x	x	x	x	x	x	x	x	x	x	x	x	x	x	x	x		
Urine pregnancy test	x	x	x		x															x		
Saliva sample					x					x			x			x				x		
Stool sample			x		x		x	x	x	x	x	x	x	x	x	x	x	x	x	x		
Blood sample	x	X	x		x	x	x	x	x	x	x	x	x	x	x	x	x	x	x	x		
12 lead ECG	x																					
Ultrasound scan	x																					
Mood assessment	x		x											x						x		
Endoscopy, fluid aspirate & biopsy [5]		X																				
Enteric String Sampling [2]			x				x			x												
Wireless video capsule endoscopy[3]							x															
Challenge with *S.* Typhi or Paratyphi					x																	
Issue study pack					x																	
e-diary entries[4]			x		x	x	x	x	x	x	x	x	x	x	x	x	x	x	x	x		
Commence antibiotics																				x		
Notification of public health and GP					x																	
Letter informing close contacts			x																			

	Enteric Fever Diagnosis visits						Follow-up visits					
Visit Number	V19	V20	V21	V22	V23	V24	V25 [6]	V26	V27	V28	V29	V30
Visit Time	ED+0	ED+12hrs	ED+24hrs	ED+48hrs	ED+72hrs	ED +96hrs	D16	D28 +/- 4 days	D21 to D48	D90 +/- 14 days	D180 +/- 28 days	D365 +/- 56 days
Consent and consent questionnaire												
Continuing consent												
Randomisation												
Revalidation of consent												
Confirm 24 hr contact details												
Medical history												
Interim medical history	x	x	x	x	x	x	x	x	x	x	x	x
Physical examination[1]	x	x	x	x	x	x	x					
Vital signs	x	x	x	x	x	x	x	x	x	x	x	x
Urine pregnancy test	x											
Saliva sample	x					x		x	x	x	x	x
Stool sample	x		x	x	x	x		x	x	x	x	x
Blood sample	x	x	x	x	x	x		x	x	x	x	x
12 lead ECG												
Ultrasound scan												
Mood assessment	x											
Endoscopy, fluid aspirate and biopsy									x			
Enteric String Sampling [2]				x			x					
Wireless video capsule endoscopy[3]				x			x					
Challenge with *S*. Typhi or Paratyphi												
Issue study pack												
e-diary entries[4]	x	x	x	x	x	x	x	x				
Commence antibiotics	x											
Notification of public health and GP	x											
Letter informing close contacts												

[1] Physical examination may be performed at other times in the study as clinically indicated.
[2] Enteric String Sampling performed **four** times: Day -7 then Day 1, Day 4 and Day 16 post-challenge. For those diagnosed with enteric fever, Day 16 will be replaced by a test at diagnosis + 48 hours. .
[3] WCE performed at **two** timepoints: on Day 1 then Day 16 (if no diagnosis) or diagnosis plus 24 to 48 hours. Initially 10 volunteers, aiming to have 3-5 WCE from those who develop enteric fever, and 3-5 from those who remained well.
[4] Solicited responses will be entered from 7 days prior to challenge to Day 21. E-diary will remain open for unsolicited entries until Day 28.
[5] Second endoscopy will not be conducted earlier than 14 days after diagnosis or antibiotic treatment ie. Those diagnosed with enteric fever will have finished their two week course of antibiotics.
[6] This visit on day 16 (visit 27) is only for a few participants (approx. 3-5 participants) who have been offered the wireless video capsule endoscopy and have not been diagnosed with enteric fever.

Table 2. Summary of Study Sampling (Part A and B)

Investigation		USS of gallbladder	Saliva sample	Stool sample	Enteric String Sampling	Wireless Video Capsule Endoscopy	Endoscopy, fluid aspirate & Mucosal biopsy	Blood culture	PCR	Bacterial quantification	Bacterial localisation/Exploratory assays	Antibody secreting cells	Blood to correlate with Mucosal Biopsy	Whole Blood Stimulation	CyTOF	Cell mediated immunity	Full blood count	CRP, U+Es, LFTs	Coeliac serology	Virology (HIV, Hep B, Hep C serology)	Serum bactericidal assay	Antibodies	Cytokines	Functional genomics	DNA samples (including epigenetics)	Glucose	
**Sample tube**								aerobic BACTEC bottle	heparinised falcon (15mL)	Isolator tube	EDTA (15mL)	heparinised falcon (50mL)	Heparinised falcon (15ml)	EDTA vacutainer	EDTA vacutainer	EDTA vacutainer	EDTA vacutainer	Heparin vacutainer	Heparin vacutainer	Heparin vacutainer	Serum vacutainer	Serum vacutainer	Heparin vacutainer	Tempus™ Blood RNA	taken from serum samples	Glucometer	
**Volume blood (mL)**								10	10	10	15	25	10 or 20	5	5	50 to 80	1 or 3	2	3	3	3	5 to 10	2	3	x	1 drop	
**Visit**	**Time**																										**TOTAL**
VO	**within** 120 days	x															3	2	3	3						x	11
V1	-180 to -45 days						x						20		5	60											85
V2	-7 days			x	x				10								1	2									13
V3	D0		x	x								20		5	5	50	1	2			3	10	2	3	x		101
V4	D0+12 hrs							10															2	3			15
V5	D1				x	x		10									1	2					2	3			18
V6	D2			x				10									1	2				5					18
V7	D3			x				10															2				12
V8	D4		x	x	x			10							5	50	1	2				5		3			76
V9	D5			x				10															2	3			15
V10	D6			x				10									1	2					2	3			18
V11	D7		x	x				10				20			5	50					3	5	2	3			98
V12	D8			x				10									1	2									13
V13	D9			x				10															2				12
V14	D10		x	x				10							5	50	1	2				5	2	3			78
V15	D11			x				10																			10
V16	D12			x				10									1	2									13
V17	D13			x				10																			10
V18	D14		x	x				10					10		10	50	1	2				5	2	3			93
V19	ED hr0		x	x				10	10	10	15						1	2			3	5	2	3			61
V20	hr12							10															2				12
V21	hr24			x				10							5	50	1	2						3			71
V22	hr48			x	x	x		10				20					1	2				5	2				40
V23	hr72			x				10																			10
V24	hr96		x	x				10					10		10	50	1	2				5					88
Volumes per assay if ED								210	20	10	10	60	20	5	45	350	14	28	3	3	9	50	26	33			
**Total blood volume in intensive phase**								**If NO enteric fever diagnosis**																			**709**
								**If enteric fever diagnosed**																			**991**
V25	D16				x	x																					0
V26	D28		x	x											5	65	1	2				10		3	x		86
V27	D21-48						x						15	3													18
V28	D90		x	x											5	50					3	5		3			66
V29	D180		x	x																	3	5		3			11
V30	D365		x	x												80					3	5		3	x		91
**Total blood volume in 1 year**								**If NO enteric fever diagnosis**																			**981**
								**If enteric fever diagnosed**																			**1263**

7.2. Recruitment (Part A and negative control group)

Identification of study participants

Several strategies may be employed in order to recruit the required cohort of participants, including:

- Open Exeter database: Potential study participants will be identified via National Health Applications and Infrastructure Services (NHAIS) who hold the central NHS patient database (Open Exeter). These databases will identify all persons within the local area who are in the appropriate age range. First contact of potential participants will not be made by the researchers at the Oxford Vaccine Group. The initial information about the study will be sent out from this agency to preserve the confidentiality of potential participants. Potentially eligible participants will be sent an invitation letter, which describes the study, with a reply slip. Anyone who is interested in taking part will be able to contact the study team by telephone, email, registering on the OVG website or by returning the attached reply slips. Potential participants will be directed to the full information booklet on the OVG website, or will be sent the information via post or email. The study team will then contact them via telephone to conduct screening for eligibility and provide further details about the study.
- Poster advertising: Display of posters advertising the study throughout local hospitals and doctor's surgeries, tertiary education institutions and other public places with the permission of the owner/ proprietor.
- Direct mail-out: This will involve direct mailing of the invitation letter to adults whose names and addresses have been obtained via the Electoral register. Those people who have indicated they do not wish to receive postal mail–shots will have their names removed prior to the investigators being given the names and addresses. The company providing this service is registered under the Data Protection Act 1998.
- E-mail communication: We will contact representatives of local tertiary education establishments and local employers and ask them to circulate posters and information, and to circulate a link to study information on the OVG website by email.
- Oxford Vaccine Centre (OVC) database for healthy volunteers: Direct email and link to members of the public who have registered their interest in potentially volunteering for clinical trials conducted by OVC. This secure database is maintained by OVC and members of the public registered here have given consent to have their details recorded and to receive a newsletter of the recruiting studies on a quarterly basis. They understand this is not a commitment to volunteering for any trial they are contacted about.

- Previously screened volunteers but not enrolled in Paratyphoid Challenge Study (OVG 2013/07) due to completion of study (dose escalation study where total number of participants required not determined). These volunteers will have had an ultrasound screening and baseline blood tests performed. If recruited to this study, repeat blood tests will be done if greater than three months since last screened; repeat gallbladder ultrasound if greater than six months.
- Media advertising: Local media, newspaper, radio, website and social media advertisement placed in locations relevant for the target age group with brief details of the study and contact details for further information.
- Website advertising: Description of the study and copy of information booklet on the OVG website. Web notices may also be posted on university websites such as the Oxford University staff gateway and Brookes Student Union website.
- Exhibitions: Advertising material and/or persons providing information relating to the study will exhibit using stalls or stands at exhibitions and/or fairs, such as University Fresher's Fairs.
- Royal Mail Leaflet: Royal Mail door-to-door service with delivery of invitation letters in OVG envelopes to every household within certain postcode areas.

Recruitment, approach and initial eligibility assessment of potential study participants

Once an expression of interest has been received by OVG, an information booklet will be sent via mail or email to the potential participants to read at their leisure. Participants can also be directed to the OVG website, where the information booklet will be available. Participants will then be phoned to discuss the study further and then invited for a screening and consent visit, where a member of the clinical research team at the Oxford Vaccine Group will assess their eligibility.

7.3. Recruitment (Part B)

Identification and contacting of potential study participants

Participants will be drawn from a pool of participants previously challenged at OVG. They will be approached via mail out, phone call or email inviting them to take part in this study. This will include information on the study and a reply slip or link to the OVG website. Anyone who is interested in taking part will be able to contact the study team by telephone, email, registering on the OVG website or by returning the attached reply slips. Potential participants will be directed to the full information booklet on the OVG website, or will be sent the information via post or email. The study team will then contact them via telephone to conduct screening for eligibility and provide further details about the study.

7.4. Informed Consent

At the consent/screening visit, a full and detailed account of the study and proposed procedures will be explained to the interested individual and accompanying parties. Written and verbal versions of the participant information booklet and informed consent form will be presented to the participant, detailing no less than:

- the exact nature of and the rationale for performing the study,
- implications and constraints of the protocol,
- the risks and benefits involved in taking part.

Additional consent will be sought from each participant:

- For a study investigator to hold the name and 24-hour contact number of a close friend, relative or housemate who lives nearby and will be kept informed of the study participants whereabouts for the duration of the study. This person is to be contacted if study investigators are unable to contact the participant. Particiapnts will agree that if their whereabouts were still unknown, study staff would make every effort to ascertain their whereabouts, e.g. attending their home. If study staff still could not locate the participant, study staff would inform the police.The 24-hour contact will receive written information, and complete and sign a reply slip that the participant will give the study doctor/nurse before challenge[1].
- To inform contacts of their involvement in the study by giving them letters provided by OVG. The letter details the study, and offers screening for bacterial carriage. The low risk of spread will be emphasised to contacts to avoid undue anxiety[1].
- For Public Health England to be informed of study participation, challenge outcome and clearance results[1].
- To contact their GP to confirm their medical history and participation in the study.
- If the participant is involved in the provision of health or social care to vulnerable groups then consent will be taken to be able to inform his/her employer of their participation in the study[1].
- Consent for endoscopy and biopsy will be taken separately by the appropriate trained individual on or before the day of endoscopy.
- Consent for enteric string sampling and wireless video capsule endoscopy will be taken separately by the appropriate trained individual on or before the day of these procedures[1].

[1] Not required for negative control group

It will be clearly stated that the participant is free to withdraw from the study at any time, for any reason and that they are under no obligation to give the reason for withdrawal. The participant will be allowed an adequate time to consider the information from when they receive it, and the opportunity to question the researcher, their GP or other independent parties to decide whether they will participate in the study.

Once satisfied, the participant will personally sign and date the approved version of the informed consent form before any study specific procedures are performed. Consent will be sought as described in relevant SOPs. Written informed consent will be obtained by means of a dated signature of the participant and a signature of the study staff member who presented informed consent. A copy of the signed informed consent will be given to the participant and the original signed form will be retained at the study site. A doctor or nurse at the OVG, who has been trained in the consent process, will conduct the informed consent discussion.

In addition the participant will be required to take a post-consent multiple choice quiz. This has been designed to test their understanding of the study and what they will be required to do to take part in the study. The participants' answers to the test will be reviewed with them by a trained study doctor or nurse and any deficient areas will be covered again until the participant is deemed to be fully informed.

7.5. Screening and Eligibility Assessment Visit

Once informed written consent is obtained, the following baseline assessments and information will be collected as part of the assessment of inclusion/exclusion criteria:

- Participant demographics; age (date of birth), gender, ethnicity and country of birth,
- Medical history, including:
    - Details of any significant medical or surgical history based on participant recall. If medical clarification is required, medical notes and/or discussion with other medical practitioners will be undertaken,
    - Confirmation from the participants GP that they know of, or do not know of, any medical condition that may affect their suitability as a volunteer,
    - Blood donation history and planning.
    - Contraception use.
    - Vaccination history.
- Females participants only:
    - Urinary pregnancy test result.

- Responses regarding any personal or domestic reason that may lead to concern regarding an individual's ability to maintain good personal hygiene[1].
- Use of concomitant medication (including over the counter medications, vitamins, illicit drug use and herbal supplements).
- History of living in enteric fever endemic areas (age, place and duration).
- Physical examination; cardiovascular, respiratory, abdominal and gross neurological examination and calculation of Body Mass Index.
- Urine dipstick (and laboratory analysis if appropriate).
- 12-lead ECG.
- Blood samples for: haemoglobin count, white cell indices, platelet count, erythrocyte sedimentation rate, serum sodium, serum potassium, serum urea, serum creatinine, liver function tests, C-reactive protein, serum amylase, anti-endomysial antibody and IgA levels, HIV and Hepatitis B and C.
- Random capillary or venous blood glucose.
- Ultrasound abdomen (to screen for gallbladder disease)[2].
- Psychological assessment by the Hospital Anxiety and Depression Score.
- Provision of the following document;
    - 24-hour contact letter (to be returned completed and signed before challenge)[3],
    - Letter to participant's close household contacts, as defined in **Appendix E**[3].

With prior participant approval, the GP will be contacted to confirm the history as given and stating whether they know of any medical reason why the participant should not be included; this reply is required prior to study enrolment.

Consent will be taken to register the participant onto TOPS.

All laboratory results will be reviewed and collated by the study team who will record these in the electronic source database. Specific guidance is provided in existing OVG SOPs. If a test result is deemed clinically significant, it may be repeated, to ensure it is not a single occurrence. If a test remains clinically significant, the

[1] While good personal hygiene preferred, this is not required for the negative control group.
[2] An ultrasound for gallbladder disease is not required for the negative control group as they will not be at risk of *S.* Typhi/Paratyphi carriage.
[3] Not required for negative control group.

participant will be informed and appropriate medical care arranged with the permission of the participant in liaison with their General Practitioner. Decisions to exclude potential participants from enrolling in the study or to withdraw a participant from the study will be at the discretion of the Chief and Co-Investigator.

The details of this assessment will be recorded in the electronic source database. If the inclusion/exclusion criteria are satisfied and informed written consent has been obtained, the participant will be randomised to receive a predetermined dose of the *S.* Typhi or *S*. Paratyphi challenge strain. For participants screened more than 90 days before the intended day of challenge, but not more than 180 days before, rescreening will be limited to confirmation of ongoing consent and repeating any investigations felt to be medically relevant by a medically qualified investigator.

Participants will also be informed that their samples would be eligible for BioBank ('Oxford Vaccine Centre Biobank' Southampton & South West Hampshire LREC (B) 10/H0504/25). BioBank is a separate study and optional to all participants of studies conducted by OVG. Separate consent is sought for this.

7.6. Randomisation and Blinding

Participants in Part A will be randomised 1:1 to receive either *S*. Typhi or *S*. Paratyphi. Participants in Part B will be stratified according to their previous exposure and then randomised to receive either *S.* Typhi or *S.* Paratyphi (described in figure 2 below) so as to achieve four groups. To elaborate, participants who have been previously challenged with *S.* Typhi will be randomised 1:1 to receive either *S.* Typhi or *S.* Paratyphi. Participants who have been previously challenged with *S.* Paratyphi will be randomised 1:1 to receive either *S.* Typhi or *S.* Paratyphi using the computer randomisation system 'Sortition'. Randomisation will take place after screening investigations are completed and when the participant has their first study procedure (endoscopy and biopsies) and study samples are taken. The participant will remain blinded to the allocation of the challenge strain; however, study staff will not be blinded. Participants will routinely be unblinded to allocation of the challenge strain at V26 (Day 28 post challenge). Unblinding may occur earlier than V26 if there are potential public health implications (e.g. If there is an outbreak of Typhoid or Paratyphoid in the community).

Enrolment is considered to be when the participant attends V1 and is randomised to *S*. Typhi or *S*. Paratyphi.

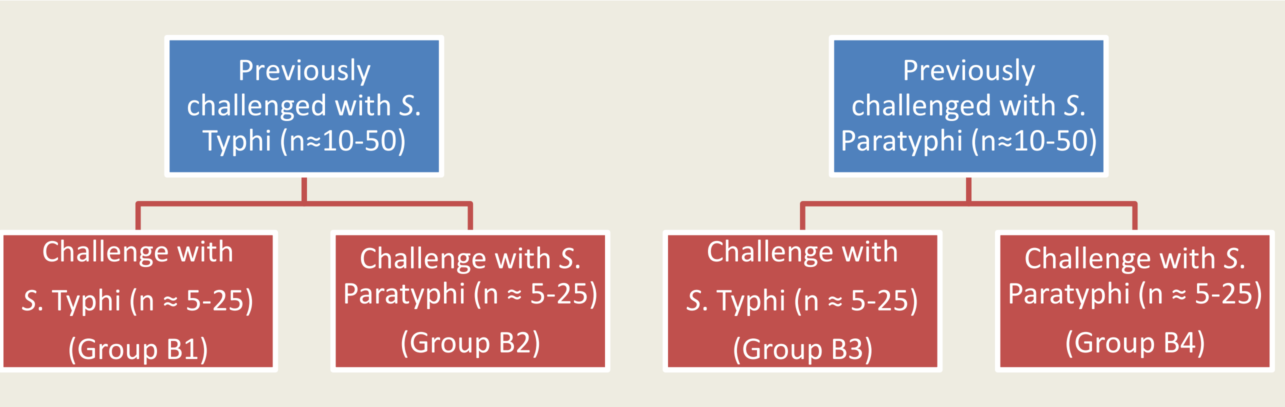

Figure 2. Example of participant randomisation to re-challenge agent.

7.7. Post-consent Visits

i. Endoscopy (Day -180 to Day -45, visit V1)

This procedure will involve video endoscopy of the upper gastrointestinal tract, and biopsy of the duodenum (D2). Endoscopy will be performed in a window between 180 and 45 days before challenge and will be carried out in the OUH Department of Gastroenterology Endoscopy Unit, according to all standard operating procedures, and to recognised national standards of safety (as monitored by Global Rating Scale). A separate consent for the endoscopy procedure, based on a local proforma used for all NHS patients, will be undertaken prior to endoscopy by an appropriately trained healthcare professional. A blood sample will be taken at this visit.

To prepare for the procedure participants will be asked to fast for four hours. For participant comfort, their throat will be numbed with an anaesthetic lignocaine spray. Light sedation (usually with intravenous midazolam) will also be offered. For safety, oxygen is given with monitoring of heart rate and blood pressure performed. It generally takes ten minutes to prepare an individual for gastroscopy and another 10-12 minutes to complete the procedure. First, a gastroscope (approximately 10 mm in diameter) will be passed through the mouth into the participants' oesophagus, through the stomach and into the duodenum (D2-3). Up to ten millilitres (mls) of fluid (distilled water) will be instilled into the duodenum through the endoscope channel, and fluid immediately sucked back, and stored for protein and antibody analysis. This is followed by flexible mucosal biopsy forceps passed through a channel in the gastroscope with up to 15 x 3 mm punch biopsy samples taken from the lining of the small bowel. As the bowel mucosa does not produce sensation the participant will be unaware of the sampling procedure. Following gastroscopy, the participant will be observed for up to two hours until the effects of any medication have worn off. Before going home, departmental staff will ensure that each participant is able to eat and drink as normal without discomfort.

ii. eDiary and first Enteric String Sampling (Day -7, visit V2)

Participants will be given access to an eDiary from seven days prior to challenge. During this time we will record any unsolicited symptoms and medications. From the day of challenge through to day 21 after challenge (28 days in total), participants will record daily symptoms and temperature. The eDiary will remain accessible till day 28 post-challenge for unsolicited symptoms. Completion of the eDiary prior to challenge will comprise of recording unsolicited symptoms only, with the aim of aiding interpretation of the post-challenge data.

Recent evidence has highlighted an important role for diet in affecting the composition of the microbiome [58].Baseline variation in diet may have a significant impact on the results of micobiome analysis before and after challenge. From Day -7 to Day – 1, participants will record a food diary for seven days alongside unsolicited symptoms, using a standardised form recorded on the online diary system (eDiary). The data collected will include type of food, brand names, cooking method and portion size, for each of breakfast, lunch, dinner and snacks.

A username and password will be given at the visit before challenge (Day -7) and they will be instructed on to how to use the software.

A stool and blood sample will be collected on this day and a urine pregnancy test on female participants performed. The first Enteric String Sampling (ESS) takes place for those who have been asked and have agreed to ESS performed during their challenge (see point viii in this section).

We will also undertake a baseline pre-challenge mood assessment using Hospital Anxiety and Depression Score (HADS).

iii. Challenge visit (Day 0, Visit V3)

Challenge visits (Day 0) will be performed in allocated outpatient clinical rooms at the CCVTM. The study will be single-blinded such that participants will not be aware of the challenge agent. Prior to challenge, the following baseline assessments will be made:

- Ensure that participant consent remains valid,
- Correct randomisation to challenge agent,
- Check participant has fasted for 90 minutes (clear fluids permitted),
- Obtain and document interim medical history since the last visit and check eligibility criteria (specifically temporary exclusion to challenge),

- Confirm 24-hour contact details[1],
- Record oral temperature, pulse and blood pressure,
- Perform sample collection, as per **Table 2.**

The suspension for ingestion will be prepared in a class II biological safety cabinet within the OVGL Containment Level 3 (CL3) suite. The cabinet will be solely used for the purpose of preparing challenge agents. The strain and bicarbonate solutions will be prepared in the laboratory and administered in the clinic room in compliance with the study-specific Laboratory Study Plan and Clinical Study Plan.

The participant will be observed for 20 minutes after administration of the challenge agent. After challenge, each volunteer will have the following information and materials provided:

- To notify the study centre of any serious adverse events/ reactions that occurs prior to next review, and any occurrence of fever ≥38°C,
- Not to use antipyretics until informed otherwise by a study investigator,
- Study centre contact details, including contact details for on call study staff member,
- Oral thermometer and instruction on its use,
- Confirm participant symptom and temperature entry into e-diary or paper backup,
- Medic Alert-type card containing information including the antibiotic sensitivity of the challenge strain, study doctor contact details and instruction for the research team to be contacted immediately in the event of illness/accident,
- Check details of a mobile phone number that the participant will be carrying with them for the 14 days post challenge and counsel the participant on the importance of keeping the mobile switched on and with them at all times,
- Information on enteric precautions and provision of liquid hand soap, paper towels and gloves to aid with adherence to enteric precautions, and information regarding the use of barrier contraception[2],
- Notification of challenge will be sent to the Thames Valley Health Protection Unit (Public Health England) and to the participants' GP[1].

Participants do not have to remain on site between assessments but a rest area will be provided for use if they wish.

---

[1] Not required for negative control group
[2] Not required for negative control group

iv. Post-challenge visits; participants who do not reach diagnosis (Day 0+12hrs to Day 365, visit V4 to V30)

These visits will require the following procedures:

- Obtain and document interim medical history since screening and check continued eligibility,
- Review e-Diary entries and laboratory blood parameters,
- Record oral temperature, pulse and blood pressure (not required in D90- D365),
- Perform mood assessment using HADS form (on D7 and D14)
- Perform sample collection as per **Table 2,**
- Issue concomitant medication for symptom control, if required (see **Table 6**),
- Schedule next visit and re-iterate participant requirements such as completion of the e-Diary, refraining from use of anti-pyretics and notification of any fever ≥38°C (where appropriate).

v. Outside of scheduled visits and unscheduled visits

Unscheduled visits will be allowed to ensure safety of the participants. History, examination and further investigation may be required to ensure participants safety. These will be at the discretion of the study investigators.

During the follow-up period, further reviews may be required depending on the participant's condition, clinical indication or at the discretion of the study staff. Every effort will be made to perform the visits on time as indicated in the study protocol. Based on previous experience with challenge studies and similar trial protocols, we are aware that in some instances this may be difficult. This is expected to be particularly difficult for visits ED+12 hours, ED+24 hours, ED+48 hours, ED+72 hours, ED+96 hours, as the time of diagnosis will not always be convenient for the participant to attend OVG. A window of time to complete the visits will be as follows:

- Day -7 +/- 4days
- ED+12 +/- 12hrs
- ED+24; ED+48; ED+72 ED+96 +/- 24 hours
- Day 28 +/- 4 days
- Day 90 +/- 14 days
- Day 180 +/- 28 days
- Day 365 +/- 56 days

vi. Enteric fever diagnosis and enteric fever diagnosis visits (visit 19 to 24)

These visits will require the following procedures:

- Obtain and document enteric fever diagnosis (see **Table 3**),
- Assessment by a study physician of diagnosis severity (**Table 4**) and potential need for in-patient admission (**Table 5**),
- Review e-Diary entries and laboratory blood parameters,
- Record oral temperature, pulse and blood pressure,
- Perform mood assessment using HADS form on the day antibiotics are commenced for enteric fever diagnsosis.
- Perform sample collection as per **Table 2.** Based on previous experience with challenge studies and similar trial protocols, we are aware that in some instance sample collection at post-diagnosis time-points may be difficult, as the time of diagnosis will not always be practical for sample processing. In order to overcome this and to facilitate sample processing, a window period of +/- 24 hours for post-diagnosis samples will be instituted.
- Issue concomitant medication for symptom control (if required) and antibiotic therapy (with confirmatory negative urine pregnancy test result) (see **Table 6**),
- Schedule next visit and re-iterate participant requirements such as continuing e-Diary entries, adherence to antibiotic therapy and maintaining contact with the study team.

Diagnosis of enteric fever

For the purposes of data analysis and reporting of enteric fever cases to Thames Valley Health Protection Unit (Public Health England), enteric fever will be defined as specified in **Table 3**.

**Enteric fever is diagnosed if ANY of the following apply**
**A positive blood culture for *Salmonella* Typhi or Paratyphi from 72 hours post-challenge**
**A positive blood culture for *Salmonella* Typhi or Paratyphi within 72 hours post-challenge, with one or more signs/symptoms of enteric fever (such as recorded temperature ≥38 °C)**

**Persistent positive blood cultures (two or more blood cultures taken at least 4 hours apart) for *Salmonella* Typhi or Paratyphi within 72 hours post-challenge.**
**Oral temperature ≥38°C persisting for 12 hours**

Table 3. Criteria for the diagnosis of enteric fever

S. Typhi or *S*. Paratyphi bacteraemia occurring before 72 hours may reflect a transient primary bacteraemia and not clinical enteric fever. For microbiological reasons, the earliest indication that a participant has a bacteraemia will be the identification of a gram-negative bacillus on Gram staining of a blood culture sample. Formal identification of the organism as *S*. Typhi/ Paratyphi may take a minimum of a further 24 hours. Participants in whom a gram negative bacillus is identified in the aerobic blood culture bottle will therefore be defined as having enteric fever for the purposes of clinical management and collection of blood, stool and saliva samples (samples as per Table 1, enteric fever diagnosis visits).

Severe enteric fever

Severe enteric fever will be defined as illness that includes any of the criteria satisfied in **Table 4**.

Severe enteric fever is diagnosed if ANY of the following apply
**Oral temperature ≥ 40ºC**
**Systolic blood pressure ≤ 85 mmHg**
**Significant lethargy or confusion**
**Gastrointestinal bleeding**
**Gastrointestinal perforation**
**Any grade 4 laboratory abnormality, as defined in APPENDIX D: Grading the severity of laboratory Adverse Events**

Table 4. Criteria for severe enteric fever

Admission to in-patient facility

Admission to the John Warin Ward (or Infectious Diseases Unit, Oxford University Hospitals NHS Trust) will be considered by a study physician under the following circumstances:

Admission to in-patient facility to be considered if ANY of the following apply
**Severe enteric fever as defined in Table 4**

**Failure of symptoms to improve within 48hrs after starting antibiotic therapy**
**Unable to tolerate oral antibiotics**
**Dehydration requiring intravenous fluid therapy**
**Unanticipated concerns about participants home conditions**

Table 5. Criteria for considering admission to in-patient facility.

In addition, any participant that deviates from the protocol and takes an antipyretic at home before the diagnosis of enteric fever will be admitted (at the discretion of the clinical study team).

Ultimately, all decisions regarding admission will be taken by the clinical study team in conjunction with the Infectious Diseases Consultant on call. The consultant will be made aware of the study protocol and the suggested treatments outlined in section 9.3, but in-patient management is at the discretion of the supervising consultant.

vii. Second endoscopy visit (visit 27)

A second endoscopy and venesection will be performed in the window period between Day 21 and Day 48 inclusive after challenge. This is regardless of whether the participant reaches enteric fever diagnosis. Further consent will be sought prior to endoscopy with the procedure conducted in the same manner as the first endoscopy. Although participants will be encouraged to undertake this procedure it is possible that some may not wish to have a further endoscopy. In this instance the participant can opt to decline the procedure and remain in the study.

Note: It is anticipated that some participants enrolling in this study may have had endoscopy and biopsy in the previous year. If this is the case we will ensure that no participant has had more than one non-study related upper GI endoscopy within the last year.

This visit will not be conducted earlier than 14 days after enteric fever diagnosis or antibiotics treatment ie. All participants will have completed their two week course of antibiotics.

viii. Enteric String Sampling

A subset of volunteers will be asked to undergo ESS. Ten participants each are required from Part A and Part B (20 in total) and once these numbers have been satisfied no further participants will be asked to have the procedure. The procedure will be conducted on participants during scheduled visit days. The ESS used in this study will be the "Enterotest™" which has been designed to sample diagnostic small bowel fluid, for diseases such as typhoid and giardiasis.

Participants will be asked to fast for four hours prior to the test. A string, enclosed in a lightly-weighted small gelatine capsule is swallowed, with the loose end of the string taped to the cheek. It is left in situ for four hours, while the string is carried into the duodenum, and the capsule dissolves. The participant will be ambulatory but fasting during this time. A rest area will be available to participants if needed. The string is retrieved after four hours by being gently pulled back through the mouth. Following retrieval of the string, the presence of bile-stained fluid is confirmed, the entire string will be placed in a sterile container, and transported to the OVG CL3 laboratory, where the fluid will be "stripped out" of the string for microbiology tests and protein assays. Participants will be requested to have this procedure on three to four occasions as outlined in **Table 1**. The procedure will be performed by trained clinical staff following local SOPs, Clinical Study Plans, and infection control processes. ESS involves minor discomfort (e.g. mild gagging during retrieval of the string, but no adverse complications have been described).

ix. Wireless video capsule endoscopy

Wireless video capsule endoscopy (WCE) will be performed by trained clinical staff following manufacturer instructions and local protocols. Participants will have gel skin pads applied, and carry a small battery pack/image capture devise. They will be asked to swallow the video capsule on two occasions – Day 1 (range D0+12hrs to Day 2) after challenge and again 24 to 48 hours after enteric fever diagnosis, or Day 16 if not diagnosed. An overnight fast and fasting for two hours (clear fluids permitted) after swallowing the capsule is recommended for good quality results.

WCE will not be performed on all participants. The study aims to have three to five results from participants from Part A and B who have developed disease and three to five from those who remained asymptomatic. As it is not possible to know beforehand the participants who will develop enteric fever, it will be offered sequentially to the first ten participants. If further numbers are required then participants who enrol later in the study may be offered the test.

x. Participant questionnaire

Following completion of their last e-Diary entry participants will be directed to an on-line questionnaire regarding their experience of the study. This questionnaire will be closely based on one used in previous challenge studies (approved by the South Central, Oxford Research Ethics Committee A), which aimed to examine volunteer motivations, attitudes and factors influencing participation in human challenge research.

7.8. Reporting cases to Public Health England

The Thames Valley Health Protection Unit (PHE) will be informed of the name, address and date of birth of all participants who;

- Satisfy the definition of enteric fever, and/or
- Have *S.* Typhi or *S*. Paratyphi cultured from any blood or faecal specimen.

In addition any breaches in enteric precautions that result in another individual coming into contact with potentially infective faeces will be reported to the Thames Valley Health Protection Unit.

7.9. Stool Culture for Carriage of *S*. Typhi or *S*. Paratyphi

The isolates of *S*. Typhi and *S*. Paratyphi used in this study have been tested and are sensitive to the first, second and third-line antibiotics of choice (see section 9.3). The duration of antibiotic therapy and exclusion of gallbladder abnormalities makes carriage of *S*. Typhi and *S*. Paratyphi unlikely. Furthermore, screening of stool samples post-antibiotic treatment will reduce the risk of carriage further. Stool samples for culture will be obtained one week after completion of the antibiotic course and then every 48 hours until three successive samples are negative. Once this criterion is satisfied, the participant will be considered fully treated for *S.* Typhi/ Paratyphi infection and no longer an infection risk. If samples remain positive for *S.* Typhi/ Paratyphi four weeks after completion of antibiotics then the participant will be referred to a Consultant in Infectious Diseases (Oxford University Hospitals NHS Trust) for further management.

The Thames Valley Health Protection Unit will be informed of all participants in whom clearance has been demonstrated and of any participant who fails to demonstrate clearance after the initial 14 day course of antibiotics or after any other antibiotic treatment course. The employer of any participant involved in the provision of health or social care to vulnerable groups will be notified in writing once three successive samples are negative.

7.10. Screening of Household Contacts

Participants in Part A and B will provide letters from the study investigators to close household contacts. Contacts will be offered the opportunity to be screened for *S*. Typhi/ Paratyphi infection, which will involve obtaining two stool samples 48-hours apart a minimum of seven days after the participant has begun antibiotic treatment. If either stool culture of a household contact is positive, he/she will be referred to a Consultant in Infectious Diseases for appropriate antibiotic management, and the Thames Valley Health Protection Unit will be informed.

7.11. Discontinuation/Withdrawal of Participants from Study

Each participant can exercise his or her right to withdraw from the study at any time. If, however, the participant decides to withdraw after they are challenged with *S*. Typhi/Paratyphi they will, for public health reasons, be required to complete a course of antibiotics and may be required to attend additional hospital/non-study visits to ensure compliance. In addition, the investigator may terminate a participant's involvement in the study at any time if the investigator considers it necessary for any reason including, though not exclusive to, the following:

- Ineligibility (either arising during the study or in the form of new information not declared or detected at screening),
- Significant protocol deviation,
- Significant non-compliance with study requirements or risk to public health,
- Any adverse event which requires discontinuation of the study procedures or leads to an inability to continue to comply with study procedures,
- Consent withdrawn,
- Lost to follow up.

Withdrawal from the study will not result in exclusion of the data generated by that participant from analysis. The reason for withdrawal, if given, will be recorded in the CRF.

i. Pregnancy

In the event of a female participant becoming pregnant during the study this outcome will be recorded and the study sponsor/ DSMC will be notified if appropriate. No further non-essential study procedures will be performed (i.e. appropriate antibiotic treatment, screening for stool carriage and/or referral may be required but further blood sampling etc. will not). The procedures and actions to be taken with a positive pregnancy test, as outlined in SOP OVG 058, will be followed.

ii. Trial Over-volunteering Prevention System

The Over-volunteering Prevention System (TOPS) is a database to guard against the potential for harm that can result from excessive volunteering in clinical trials involving IMPs or blood donations. Participants will be registered with this system at screening using their national insurance number, or passport number if they do not have a national insurance number. The system will be updated in the event of the participant not entering the study, being withdrawn or excluded. Alternatively TOPS will be updated on the participant's last visit.

7.12. Definition of End of Study

The clinical phase of the study has ended when the last volunteer completes the last visit. The definition of the end of the study is when the last assay is performed.

7.13. Potential Benefit to Participants

Participants will not directly benefit from participation in this study. However, it is hoped that the information gained from this study will contribute to the development of an understanding of enteric fever. The only benefits for participants will be information about their general health status.

7.14. Potential Risk to Participants

The general risks to participants in this study are associated with study-fatigue, risks of study procedures, symptomatic infection and the small risk of subsequent complications.

i. Phlebotomy

The volume of blood drawn over the study period should not compromise healthy adult volunteers. Mild tenderness, bruising, light-headedness or rarely syncope, may result from venepuncture.

ii. Wireless video capsule endoscopy

Complications are rare but retention of the capsule and/or resultant gastro-intestinal obstruction is reported. This is extremely unusual in healthy individuals without underlying stricturing bowel disease. In clinical practice this complication usually only occurs because of occult GI stricturing sometimes encountered during investigation for symptomatic GI conditions such as Crohns Disease. Participants will be encouraged to report any abdominal pain after swallowing the capsule and will be closely monitored by the study team. In the event of the individual experiencing pain or any other symptom suggestive of retained capsule or obstruction, they will be reviewed by the OUH Gastroenterology team, who will decide whether further investigation, monitoring or intervention is appropriate.

iii. Endoscopy and biopsy

The British Society of Gastroenterology describes endoscopy as a very safe procedure. Most people experience no adverse effects as a result of endoscopy. The most common adverse effects are mild (sore throat / abdominal discomfort). On extremely rare occasions, the procedure is associated with more serious complications. Diagnostic endoscopies have an associated total complication rate of < 0.1%, with an associated mortality of 0.004%. Potential complications associated with endoscopy are detailed below:

Perforation: Perforation is the most serious complication associated with diagnostic endoscopy. Perforation is very rare (approximately 0.01%) with an associated mortality of 0.001% (BSG Guideline 2006, Riley). Perforation is most commonly associated with biopsy of an underlying cancer, or the presence of a structural abnormality such as a pouch or stricture which is usually but not always symptomatic before endoscopy – these additional risk factors are not likely to pertain to our healthy volunteers. Cervical perforation and small biopsy-related “microperforations” can usually be managed conservatively, but occasionally thoraco-abdominal perforation requires surgical repair (BSG Guideline 2006, Riley). Although there is a theoretical increased risk of perforation when introducing a potentially ulcerating gastrointestinal infection, it should be noted that biopsies are taken from the proximal duodenum, and the ulceration described in enteric fever occurs more distally in the ileum. As a precaution, however, at least 45 days will be left between initial biopsy and subsequent challenge with *S.* Typhi or *S*. Paratyphi, and the **second endoscopy and biopsy will not be performed until a minimum of 14 days after diagnosis or antibiotic treatment and clinical recovery**.

During a recent study that involved mucosal biopsies (REC Ref: 13/NW/0282, Understanding Immunity to Typhoid), a participant was diagnosed with a retroperitoneal perforation post-endoscopy. This was managed conservatively. Independent opinion from three endoscopists felt the use of jumbo forceps contributed to this SAE as they can intrude into the submucosa of the small bowel lining. Consequently, this study will use flexible, large capacity, mucosal biopsy forceps to take up to 15 x 3 mm punch biopsy samples. The depth of biopsies from large capacity forceps is less than that of jumbo forceps, theoretically reducing the risk of perforation.

Bleeding: Mucosal bleeding which requires intervention is very rare following diagnostic endoscopy. Mucosal bleeding may be managed by standard endoscopic methods including the use of local adrenaline injection, placement of clips, or the application of direct thermal haemostatic methods, which usually terminated bleeding. Very rarely angiography with embolisation or surgery may be necessary (BSG Guideline 2006, Riley).

Cardiorespiratory Events: Cardiorespiratory events, usually related to over-sedation, are most common in elderly patients and in people with underlying comorbidity. This risk can be minimised by routine safe conscious sedation practice, using the lowest required dose of sedation (BSG Guideline 2003, Teague).

Infection: Nonclinically significant transient bacteraemia is common following gastroscopy, as it is following other minor upper GI trauma, such as tooth-brushing. Prophylactic antibiotic treatment is no longer recommended (BSG Guideline 2009, Allison).

iv. Symptomatic infection

Study participants may develop symptomatic typhoid or paratyphoid infection following challenge. During the challenge phase (before treatment with antibiotics) participants will be reviewed at least daily by the clinical study team and will, in addition, be telephoned or contacted by text message every evening to ensure that

they are safe. Symptoms of fever, headache, malaise, anorexia, abdominal pain, nausea/vomiting, myalgia and arthralgia, cough, rash, diarrhoea and constipation will be actively enquired about at each post-challenge visit. Participants will be instructed to record their oral temperature with a thermometer provided should they feel feverish, and will be instructed to contact the investigator immediately should they manifest any signs or symptoms they are concerned about.

v. Study-fatigue

This may occur due to the intense nature of the study procedures, especially during the challenge period. The participants are reimbursed for their time and every effort will be made to make study investigations as swift and uncomplicated as possible. Interventions and visits will be limited in number and will be arranged to fit with individual schedules and other obligations, as far as is practical.

vi. Complications of typhoid and paratyphoid infection

The antibiotic management of typhoid and paratyphoid fever is outlined in Section 9.3; fluoroquinolone antibiotics are the treatment of choice for enteric fever and are generally well tolerated and rapidly efficacious[9,42]. If any participant is unexpectedly unwell then additional visits will be arranged by the study team. If it is deemed necessary for safety reasons, a home visit will be performed. The risks associated with typhoid and paratyphoid challenge are greatly minimised by participant compliance with study visits and maintenance of close contact with the study team. This will be emphasised at screening and throughout the study.

7.15. Potential Risks to Close Contacts of Participants

In view of the low infectivity of *S*. Typhi and *S.* Paratyphi and the level of hygiene and sanitation in the UK, secondary transmission of typhoid and paratyphoid to household contacts or other close contacts is highly unlikely. It is thought that *S.* Typhi/ Paratyphi, unlike *Shigella*, enterohaemorrhagic *Escherichia Coli* or Hepatitis A virus is virtually never transmitted by direct faecal-oral contact. This is believed in part to be due to the requirement for ingestion of a higher inoculum of *S.* Typhi/ Paratyphi bacteria to cause clinical illness, as was found in the initial typhoid challenge model performed in Oxford (OVG 2009/10). The only (rarely) reported exception is direct contact transmission of *Salmonella* sp. by ano-lingual sexual contact[59]. For this study we discuss with participants what bodily fluids are potentially infective (stool) and that sexual practices without barrier methods may risk transmission of the infection to a sexual partner, albeit this risk is small.

The potential for transmission to occur within a household may increase if the individual excreting *S.* Typhi/ Paratyphi fails to practice hand washing after defecation and proceeds to prepare food in a way that the food

becomes inoculated. If food is kept at ambient temperature, *Salmonella* bacteria can proliferate, reach an infective dose, and the food vehicle may then transmit infection.

Throughout the period of possible excretion the challenged participants must practice stringent hand washing techniques, post-defecation and prior to contact with food. Participants will be given hand soap and paper towels for use at home and detailed practical advice on how to prevent transmission of faecal-oral infections. Participants will be taught and then observed practicing good hand hygiene technique. The importance of adhering to sanitation advice will be emphasised at each visit. It is important to note that participants in this study will be fully informed about the risks of transmission and how to prevent this, prior to challenge, and as such will be in the position to implement this from the point of infection reducing the chances of secondary transmission to near zero. This is very different from the situation with travellers returning from abroad where diagnosis is usually delayed by several weeks allowing a prolonged period of exposure to contacts before precautions are put in place. In practice, since most individuals living in developed countries practice good personal hygiene and food hygiene, secondary transmission of *S.* Typhi/ Paratyphi or within households by returning travellers with enteric fever is indeed rare. We will treat all participants in this study very early in the course of disease, leading to the rapid clearance of bacteria and a very limited period of excretion and potential exposure to contacts.

When occasional transmission of enteric fever occurs, it is usually related to unknowingly infected food handlers; for this reason food handlers will be excluded from this study[60,61].

Even in the absence of precautions to prevent secondary transmission of enteric fever (as is seen in travellers), the rate of transmission is exceptionally low within the UK. In a large and recent study of 251 contacts of patients with enteric fever in London, only one person was identified as a suspected case of secondary transmission[62]. Similarly a study in Scotland showed a maximum secondary transmission rate in the absence of precautions to be six out of 267 contacts[63].

8. INTERVENTIONS

The *S.* Typhi and *S*. Paratyphi challenge agent will be administered by the oral route with a sodium bicarbonate solution. Participants will be nil by mouth for 90 minutes before and after challenge. Participants will drink 120 ml of a bicarbonate solution; one minute later the participants will ingest the challenge organisms suspended in 30 ml of bicarbonate solution.

8.1. *S.* Paratyphi Strain

i. The challenge strain (NVGH308)

The challenge strain is provided by Novartis Vaccines for Global Health, having been manufactured to GMP standard (see below). The NVGH308 strain has been serotyped as *Salmonella enterica* serovar Paratyphi A and was originally isolated from a patient in Kathmandu, Nepal, who was participating in a clinical study undertaken by the Oxford University Clinical Research Unit.

ii. GMP manufacture of the challenge strain (NVGH308)

A parent seed lot, S888P5SP01, was established in March 2010 after serial colony selections on Luria Broth PTK agar plates and stored in the Novartis Vaccines and Diagnostics bacterial seed bank (Siena, Italy). This lot was used to establish the GMP Master Cell Bank, SA-13-002. Under GMP conditions in GenIbet BioPharmaceuticals, Portugal, three dose levels of the challenge agent were produced in chemically-defined media, using sucrose as the carbon source, to prepare a bulk bacterial suspension (Active Substance, 00513). Prepared vials containing the challenge agent were stored at -80°C ± 5°C and part of this batch (243 vials) transferred to the Oxford Vaccine Group Laboratory in 2014.

Strain characterisation has included:

- Serotyping confirms *Salmonella enterica* serovar Paratyphi A.
- Antibiotic sensitivity profile of the challenge agent in June 2013 demonstrated a fully antibiotic sensitive strain.
- Further antibiotic sensitivity profiling of the challenge agent in December 2013 in Oxford University Hospital's laboratory demonstrated full antibiotic sensitivity.
- Analysis by the Novartis Vaccines Institute for Global Health confirms presence of the O:1 and O:2 polysaccharide antigen.
- Biochemical profiling.

A challenge study using this strain was undertaken at OVG (REC no. 14/SC/0004 ,OVG 2013/07 Understanding Paratyphoid Infection).[Dobinson et al, in press]. Using the results of the previously performed dose-finding study (OVG 2013/07), the required dose will be administered to provide an inoculum of 1-5x10^3 CFU.

Storage of the study treatment

S. Paratyphi (NVGH308) for inoculation of participants will be stored as a frozen suspension in soya tryptone medium containing 10% sucrose. Suspensions have been labelled with the contents (*S*. Paratyphi NVGH308 strain), date of manufacture, CFU information, storage conditions, batch and vial number.

Vials of the required concentration of *Salmonella* Paratyphi (NVGH308) will be thawed and diluted immediately prior to use.

8.2. *S.* Typhi Strain

i. The challenge strain (Quailes)

The *Salmonella* Typhi (Quailes strain) was originally supplied by Maryland University and then transferred to the Health Protection Agency (Porton Down, Salisbury, UK) and manufactured to GMP standards. It has been used as a challenge agent for 132 healthy volunteers since 2009 in Oxford. Using the results of the previously performed dose-finding study (OVG 2009/10), the required dose will be administered to provide an inoculum of 1-5x10^4 CFU.

ii. GMP manufacture of the challenge strain (Quailes)

A master cell bank (OX-SQ-MCB1) was generated in 2009 at the Oxford site which was then formally transferred to a GMP site. GMP batches were then shipped back to, and stored in Oxford under controlled conditions. These were then subjected to quality control and characterisation including gram stain, antibiotic sensitivity (fully sensitive), purity and content testing.

iii. Storage of the study treatment

The *Salmonella* Typhi (Quailes strain) for inoculation of participants have been stored as a frozen suspension in soya tryptone medium containing 10% sucrose. Suspensions have been labelled with the contents (*S*. Typhi Quailes strain), date of manufacture, CFU information, storage conditions, batch and vial number.

Vials of the required concentration of *Salmonella* Typhi (Quailes stain) will be thawed and diluted immediately prior to use.

8.3. General considerations for both strains

i. Accountability of the study treatment

Doses of *S.* Typhi/ Paratyphi prepared for inoculation of study participants will be accounted for within an accountability log. The investigator will track all vials received and used and OVG will retain all unused products.

The clinical study team will be responsible for adequate and accurate accounting of *S.* Typhi and *S*. Paratyphi vials prepared for inoculation of participants. The clinical study team will administer the study inoculum only to individuals included in this study following the procedures set out in this study protocol. The date, dosage and time of participant inoculation will be recorded.

ii. Concomitant medication

Any medication taken by the participant at the time of enrolment into the study or during the first 90 days of the study period will be recorded on the eCRF. Medications as outlined below will be provided by the clinical study team as required.

iii. Concomitant medication for symptoms of enteric fever

Concomitant medication can be provided for symptomatic control of enteric fever, before and after diagnosis. Concomitant medication after enteric fever diagnosis includes antibiotics and anti-pyretics if required.

Drug	Indication	Dose	Route	Frequency
Paracetamol	Fever and/or pain (**after antibiotic therapy started**)	1 gram	Oral	PRN, max QDS
Codeine	Pain	30-60mg	Oral	PRN max. 240mg/24hours
Senna	Constipation	2-4 tablets	Oral	PRN
Domperidone	Nausea	10-20mg	Oral	PRN (max 80mg in 24hrs)
Chlorpheniramine	Allergy symptoms	4mg	Oral	PRN 6-8 hourly, max 24mg in 24hrs

Table 6. Concomitant medication for symptom control.

iv. Antibiotic treatment

Antibiotic therapy is commenced when one or more of the criteria in **Table 7** are satisfied.

Antibiotics are commenced if ANY of the following apply
**Any participant meeting the definition of enteric fever**
Any participant with 3 or more of the following symptoms *severe enough to interfere with all normal activity*;<br>• **Headache**<br>• **Malaise**<br>• **Anorexia**<br>• **Abdominal pain**<br>• **Nausea/vomiting**<br>• **Myalgia**<br>• **Arthralgia**<br>• **Cough**<br>• **Rash**<br>• **Diarrhoea**<br>• **Constipation**
**Any participant who has not received antibiotics by day 14 post-challenge**
**Any participant in whom antibiotic use is felt to be clinically necessary** (as decided by a medically qualified study investigator)

Table 7. Criteria for commencing antibiotic treatment.

The first line antibiotic is oral Ciprofloxacin 500mg twice daily for 14 days. Side-effects of ciprofloxacin are detailed in The British National Formulary. The most common side-effects include gastro-intestinal upset, rash, dizziness or itch. Participants will be informed of possible side-effects of antibiotic treatment before enrolment into the study. Cautions and contra-indications for the use of ciprofloxacin include:

- Pregnancy – a negative pregnancy test is required of all female participants of childbearing potential prior to treatment,
- Quinolone antibiotics can cause QT interval prolongation, increasing the risk of ventricular arrhythmias in people predisposed to QT prolongation. For this reason, participants with evidence of QT interval prolongation on screening ECG or a history of cardiac arrhythmia will be excluded from the study.

Any participant in whom a contra-indication to ciprofloxacin becomes apparent (as per The British National Formulary) or if response to treatment is slower than expected, the following regimens of licensed antibiotics will be used:

- 2nd line: Oral azithromycin 500mg once daily for 14 days,
- 3rd line: Oral trimethoprim/sulfamethoxazole 160/800 mg once daily for 14 days.

Participants will be asked to take the first antibiotic dose in the presence of a study investigator. They will be telephoned or contacted by text message to remind them to take further antibiotic doses. A participant's general practitioner will be notified in writing of the antibiotics received.

v. Contraception, antibiotic treatment and enteric fever symptoms

The possible combined adverse effects on pregnancy of *S.* Typhi and *S*. Paratyphi infection and the procedures and antibiotics given in this study, are unclear. As such, pregnant women are excluded from participating in this study and female participants are required to use an effective form of contraception from 30 days prior to challenge and then until three clearance stools are negative. In order to demonstrate clearance, the first stool sample will be collected at least one week after completion of antibiotics. Two further stool samples should be collected at least 48 hours apart.

It is only some antibiotics (those in the rifamycin class) that reduce the efficacy of oral hormonal contraceptives. However, for this study female participants will be advised to use additional barrier contraception while on antibiotics.

Diarrhoea and vomiting can reduce the efficacy of oral hormonal contraceptives ('pill') by altering absorption. If vomiting occurs within two hours of taking a pill, then another pill should be taken straight away. If there is continued vomiting or significant diarrhoea (passing six to eight stools in 24 hours) for more than 24 hours, this is counted as a missed pill day and extra contraception, such as condoms, may be recommended dependant on the type of oral contraception used (i.e. combined versus progesterone-only pills). The clinical study team will follow the NHS contraceptive guide (www.nhs.uk/conditions/ contraception-guide) if these issues arise.

vi. Post-study treatment

Study medication will not be continued beyond the study period.

9. LABORATORY

An interim sample analysis will be performed for the assays described in this section to confirm optimal sample number and collection time points. Based on these results, the number of participant samples required could

allow fewer participants to be recruited. It is anticipated that some sample collection time points will be dropped.

9.1. Blinding of laboratory samples

Details regarding the process of blinding laboratory samples and the laboratory methods to be used are given in the OVG Clinical and Laboratory Study Plans. Samples processed by departments at the Oxford University Hospitals will be processed using the study identifier and participant study number only.

9.2. Bacteriology

Blood culture

After inoculation of aerobic broth with 10mls of participant blood (BACTEC PLUS Aerobic/F culture vial; BD, Oxford, UK), culture will be performed using the BACTEC 9240 continuous monitoring system in the microbiology laboratory of the Oxford University Hospitals NHS Trust, according to the current version of M-SOP-017 Blood Culture. Identification of organisms cultured will be by biochemical (API, Analytical Profile Index; bioMérieux, Basingstoke, UK) and serological methods, latterly by agglutination according to the Kauffman-White classification. Blood cultures obtained out-of–hours will be transported to the microbiology lab by courier. Blood culture results out-of-hours will be phoned to the on-call study doctor by the microbiology department.

Quantitative culture of whole blood will be performed to determine the number of organisms in the blood, using the Isostat® Isolator system (Alere, Basingstoke). Enumeration of *S.* Typhi/ Paratyphi organisms in the blood will be performed by lysis centrifugation followed by direct plating onto non-selective media.

Stool and enteric fluid sample culture

Routine stool cultures and screening will be performed by the microbiology laboratory, OUH, according to the current version of M-SOP-111 Stool Culture. Stool and enteric fluid samples will be inoculated directly onto XLD agar for semi-quantitative culture and into Selenite F enrichment broth for qualitative culture. After overnight incubation (at 37°C), each sample will be sub-cultured onto Salmonella-selective chromogenic agar (SALM agar, E&O Laboratories Ltd, Bonnybridge, Scotland). Suspected colonies will be identified as per the blood culture method described above.

Stool cultures will be taken at baseline, throughout the 14 day post-challenge period and at visits after enteric fever diagnosis. Participants will be required to supply two stool samples after completion of antibiotics to

screen for carriage. If the participant works with high-risk groups then they will require three negative samples. This is in concordance with guidance from Public Health England.

Isolates of *S*. Typhi/ Paratyphi may be retained for phage typing or further investigation by the reference laboratory if challenge strain confirmation is required by Public Health England.

Quantitative stool and enteric fluid cultures will be performed at the OVG Laboratory, to assess the burden of enteric luminal replication and stool shedding. Isolates from stool and enteric fluid samples will be stored frozen for future analysis, which may include phage typing or genetic sequencing.

Stool microbiome

Analysis of the relative composition of bacterial populations within the bowel, will be performed on collected stool samples. The faecal microbiome and its effects on challenge outcome, bacterial dynamics and response to antibiotic treatment will be assayed using techniques including pyrosequencing. After collection stool samples will be stabilised in RNA-later and stored at -80°C, prior to further assessment.

Cellular Localisation

Culture based assays to identify cellular localisation of S. Typhi and S.Paratyphi at the time of diagnosis will also be performed in order to further describe the bacterial dynamics after challenge as outlined in the secondary objectives of the study.

9.3. Molecular diagnostics

To develop and validate possible molecular diagnostics for the purposes of improving on currently available diagnostic tests, samples will be collected for techniques including the Blood culture-PCR methodology described briefly below.

Blood culture PCR detection

The Oxford Vaccine Group has developed a fast and highly sensitive novel TSB-bile blood culture-PCR assay . We will use this and other novel methodologies s to detect low levels of *S.* Typhi/ Paratyphi in the blood of participants after challenge[64].

DNA will be isolated using the UltraClean® BloodSpin® kit (MO BIO Laboratories Inc., CA, USA) according to the manufacturer's instructions. Isolated DNA will be used as a template for organism detection by PCR amplification.

9.4. Inflammatory responses

Plasma Cytokines

The kinetics of the inflammatory response will be measured by assay of stored plasma or serum samples for concentration of cytokines, including IL-1β, IL-2, IL-4, IL-5, IL-6, IL-8, IL-10, IL-12, TNF-α and IFN-γ. Plasma/serum samples will be isolated from blood and stored at -80°C. A custom commercial multiplex bead-array kit (Millipore, Germany) or an alternative methodology will be used.

Faecal lactoferrin/calprotectin

Stool and enteric fluid samples may be assayed for lactoferrin and calprotectin, or other markers of gastrointestinal tract inflammation. Samples will be stored at -80°C and lactoferrin and calprotectin measured using commercially available ELISA kits.

9.5. Antibody responses

Serum

Serum will be isolated from blood and stored at -80°C. Samples will be tested for IgG, IgM, and IgA antibodies to a panel of antigens including *S.* Paratyphi O and H antigens or *S.* Typhi O, H and Vi antigen.

The antibody response may also be tested in other assays using additional antigens or methodology. The functional activity of antibodies will be tested using serum bactericidal, opsonophagocytosis or alternative assay.

Antibody in Lymphocyte Supernatant

Briefly, isolated PBMCs and gut mucosal mononuclear cells will be cultured for 24 hours before the supernatants are harvested and stored at -80°C. Specific IgG, IgM and IgA antibodies to a panel of *S*. Typhi/ Paratyphi antigens will be measured by ELISA or an alternative method.

Saliva

Total and isotype specific salivary immunoglobulin to a panel of *S*. Typhi/ Paratyphi antigens will be measured by ELISA or alternative assay.

***S*. Paratyphi-specific coproantibodies**

Specific IgG, IgM and IgA antibodies against S. Typhi and *S*. Paratyphi antigens will be measured in stool samples. If specific IgA is detected, IgA1 and IgA2 subclass determination may be performed.

Mucosal immune responses

Gut-derived, trafficking, antibody-secreting cells (ASC) that secrete IgA or IgG antibody against S. Typhi and *S.* Paratyphi O or H antigen will be measured by ELISPOT. Briefly, PBMC will be cultured in antigen-coated ELISPOT plates. Specific IgA, IgM and IgG will be determined by counting coloured spots. Enteric fluid samples will be tested for immunoglobulin responses using ELISA or alternative methods.

B cell repertoire

PBMC's isolated from blood will be used for high-throughput sequencing of B cell receptor (antibody) genes. This can be used to give an overview of the entire repertoire of different B cells that are circulating in the blood. Custom bioinformatic tools will be used to look at global changes in the repertoire following challenge, which could give insight into which B cell clones are expanding in response to challenge, and are therefore likely to be producing pathogen-specific antibodies. Knowledge of the genetic sequence of pathogen-specific B cell clones could be used in the creation of monoclonal antibodies. In addition, it is possible that measuring the abundance of certain pathogen-specific B cell clones in the repertoire could be used as a correlate of protection.

9.6. Cellular immune responses

Cellular immune responses will be analysed using PBMCs, gut mucosal mononuclear cells and/or whole blood. Assays performed may include activation, proliferation and cytokine measurement using techniques including intracellular staining and multi-chromatic flow-cytometry of *in vitro* stimulated cells. Additional analysis will be performed using heavy-metal ion tags (CyTOF) to increase the number of parameters being investigated. These investigations will be performed by the OVG Laboratory and the Human Immunology Group at the Weatherall Institute of Molecular Medicine (WIMM), Oxford.

T cell repertoire

T cell repertoire will be analysed with high resolution sequencing techniques on total lymphocytes or on subsets of antigen specific T cells.

CD1 and MR1 restricted T cell responses

Frequency of CD1 and MR1 restricted T cells will be assessed on PBMC and gut mucosal mononuclear cells. Assays performed may include activation, proliferation and cytokine measurement using techniques including intracellular staining and multi-chromatic flow-cytometry of *in vitro* stimulated cells, and ELISPOT and proliferation assays on T cells stimulated with cell lines transfected with CD1 and MR1 molecules.

9.7. Factors affecting susceptibility and response to infection

DNA analysis

Analysis of DNA sequencing or the association with single-nucleotide polymorphisms (SNPs) and epigenetics will be performed using DNA extracted from clotted blood, derived from collected serum samples. With consent, these samples will be used to identify host factors for susceptibility and response to challenge using a candidate gene approach.

Functional genomics

Blood and gut mucosal biopsies will be collected and initially stored at -80°C. RNA will be isolated and used for the study of gene expression profiles. Gene expression profiles will be determined using commercially available gene expression microarrays, the Nanostring platform and/or mRNA-seq technologies.

Whole blood/PBMC *Salmonella* Typhi/Paratyphi killing assay

This exploratory assay aims to confirm that cells from blood taken from protected individuals (those previously exposed and not diagnosed with enteric fever within 14 days of challenge) will kill S. Typhi/Paratyphi more effectively than cells from blood from non-protected individuals. Whole blood or PBMC will be used to set up an in-vitro challenge with *S*. Typhi/Paratyphi. The bacteria will be spiked into blood/PBMC and growth/death of the bacteria will be assessed at defined timepoints after inoculation. A range of immunological methods will be used to assess the mechanism of in-vitro killing.

Mass spectrometry

Enteric fluid and serum proteins and other molecules may be analysed by SELDI-TOF mass spectrometry according to established protocols, developed in collaboration with Imperial College London Medical School.

9.8. Other laboratory investigations

Laboratory tests such as white blood cell count (WBC), differential counts, C-reactive protein, urea, creatinine, electrolytes, Aspartate Aminotransferase (AST), alkaline phosphatase (ALT), bilirubin, and amylase will be performed using the OUH, NHS laboratories. Blood samples will be collected and delivered to OUH clinical laboratories for analysis according to national SOPs.

Samples collected as part of this study may also be used for other exploratory studies of scientific relevance by the OVG Laboratory or any of the collaborating laboratories. These samples will be material deemed 'not relevant' under the Human Tissue Act (HTA). Studies may include further investigation of the inflammatory and immunological response to vaccination and/or challenge.

Buffy coat cells will be ordered through the National Blood Service to be used for assay controls and reported in future publications. Blood from healthy consenting volunteers will also be used as assay controls.

10. SAFETY REPORTING

According to current recommendations from the Medicines and Healthcare products Regulatory Agency, challenge agents do not constitute Investigational Medicinal Products or 'treatment'.

10.1. Definitions of an Adverse Event (AE)

An AE is:

Any untoward medical occurrence in participants following administration of *S*. Typhi or *S*. Paratyphi, which does not necessarily have to have a causal relationship with *S*. Typhi or *S*. Paratyphi. An AE can therefore be any unfavourable and unintended sign (including an abnormal laboratory finding), symptom or disease temporally associated with the ingestion of *S*. Typhi or *S*. Paratyphi, whether or not considered related to this.

10.2. Definition of an Adverse Reaction (AR)

All untoward and unintended responses to challenge related to any dose or any study procedure.

The phrase "responses to challenge" means that a causal relationship between *S.* Typhi or *S.* Paratyphi challenge and an AE is at least a reasonable possibility, i.e. the relationship cannot be ruled out.

All cases judged by either the reporting medically qualified professional or the Sponsor as having a reasonable suspected causal relationship to the challenge agent qualify as adverse reactions.

For the avoidance of doubt, if the reaction to *S*. Typhi or *S*. Paratyphi is within a defined set of criteria as listed under 'Expected Adverse Events' (see section 10.3), which may be expected in an individual developing symptomatic enteric fever, then these will be classified as outcomes and not as adverse events.

Adverse events may be identified if the participant is specifically asked to record their presence/absence/severity in the diary card or if the participant volunteers these spontaneously.

10.3. Definition of an Expected Adverse Event

Any expected adverse event (as specified in Table 8), starting between day 4 and day 14 post-challenge (or PD +96 hours, whichever comes first) will be deemed 'Expected Adverse Events' and documented in the participant eDiary and/or the eCRF. Expected events will not be included in the 'Adverse Event' section of the eCRF. Expected events can be recorded as adverse events, at the discretion of the investigators, if they were believed to be unusual (for example in severity, frequency, duration or character).

Expected Events
**headache**
**fever >37.5°C but <40°C (A fever over 40 degrees will be considered an AE).**
**generally unwell (malaise)**
**loss of appetite (anorexia)**
**rash**
**constipation**
**diarrhoea**
**abdominal pain/distension**
**myalgia or arthralgia**
**cough**
**nausea or vomiting**

Table 8. Events which will be attributed to development of symptomatic enteric fever

Causality assessment will follow the guidelines from existing OVC SOPs, with the exception of causality attribution and classification that will be based on the following criteria:

No relationship

- No reasonable temporal relationship to ingestion of challenge agent, *and*
- Alternative aetiology (clinical, environmental or other intervention), *and*
- Does not follow pattern of recognised response to *S*. Typhi or *S*. Paratyphi infection.

Possible

- Reasonable temporal relationship to ingestion of challenge agent, *or*
- Event not readily explained by alternative aetiology (clinical, environmental or other interventions), *or*
- Similar pattern of response to that seen with *S*. Typhi or *S*. Paratyphi infection.

Probable

- Reasonable temporal relationship to ingestion of challenge agent, *and*
- Event not readily produced by alternative aetiology (clinical, environment, or other interventions), *or*
- Known pattern of response with *S*. Typhi or *S*. Paratyphi infection.

Definite

- Reasonable temporal relationship to *S*. Typhi or *S*. Paratyphi ingestion, *and*
- Event not readily produced by alternative aetiology (clinical, environment, or other interventions), *and*
- Known pattern of response to *S*. Typhi or *S*. Paratyphi infection.

10.4. Definition of a Serious Adverse Event (SAE)

A serious adverse event is an AE that results in any of the following outcomes, whether or not considered related to challenge agent ingestion:

- Death.
- Life-threatening event (NOTE: The term "life-threatening" in the definition of "serious" refers to an event in which the participant was at risk of death at the time of the event; it does not refer to an event which hypothetically might have caused death if it were more severe).
- Hospitalisation or prolongation of existing hospitalisation.
- Results in persistent or significant disability/incapacity.
- Is a congenital anomaly/birth defect.
- Other important medical events. NOTE: Other events that may not result in death are not life threatening, or do not require hospitalisation, may be considered a serious adverse event when, based upon appropriate medical judgement, the event may jeopardise the patient and may require medical or surgical intervention to prevent one of the outcomes listed above.

To ensure no confusion or misunderstanding of the difference between the terms "serious" and "severe", which are not synonymous, the following note of clarification is provided:

The term "severe" is often used to describe the intensity (severity) of a specific event (as in mild, moderate, or severe myocardial infarction); the event itself, however, may be of relatively minor medical significance (such as severe headache). This is not the same as "serious," which is based on patient/event outcome or action criteria usually associated with events that pose a threat to a participant's life or functioning as defined in the bullet points above. Seriousness (not severity) serves as a guide for defining regulatory reporting obligations.

10.5. Definition of a Serious Adverse Reaction (SAR)

An adverse event that is both serious and, in the opinion of the reporting investigator, believed with reasonable probability to be due to challenge or study procedure, based on the information provided (see causality assessment section 10.3).

10.6. Adverse events of special interest (AESI)

The following events will be considered AESIs:

- Complications of severe enteric fever (as defined in **Table 4**)

  Complications such as perforation or haemorrhage occur almost exclusively in patients who are untreated for an extended period. Participants in this study will be treated 12 hours after developing fever or if *S.* Typhi or *S.* Paratyphi is recovered from a blood culture drawn 72 hours after challenge. They will be closely monitored during the initial study phase, until a 14-day course of antibiotic is completed, so that the risk of complications is small.

- Failure to clinically or bacteriologically cure a participant of enteric fever infection within 14 days of antibiotic therapy, progression to chronic carrier state or relapse.

  A chronic carrier state in which *S*. Typhi/ Paratyphi is excreted in the stools for many years without illness can develop after ingestion. The chronic carrier state is usually seen in older women with pre-existing gallbladder disease (mainly gallstones). We will only enrol participants with a normal ultrasound examination of the gallbladder. Stool sample for culture will be obtained upon completion of the initial antibiotic course and then weekly until two (three for health and social care workers) subsequent samples are negative to detect the chronic carrier state. Should chronic carriage occur (defined as stool cultures being positive for *S*. Typhi or *S.* Paratyphi 4 weeks after completion of antibiotics) then the participants will be referred to a Consultant in Infectious Diseases (Oxford University Hospitals NHS trust) for further management.

- Transmission of *S*. Typhi or *S.* Paratyphi to a contact of a participant.

  In view of the low infectivity of *S.* Typhi or *S.* Paratyphi without sodium bicarbonate buffer and the standard level of hygiene and sanitation in the UK, secondary transmission to household contacts or other contacts is highly unlikely. Strict precautions will be emphasised by the study staff.

  Household contacts of participants will be offered the opportunity to be screened for *S*. Typhi or *S.* Paratyphi by examination of up to two stool specimens, taken 48 hours apart a minimum of seven days after the participant has commenced antibiotic therapy. In the unlikely event that a contact tests positive, Public Health England will be informed.

- AEs requiring a physician visit or Emergency Department visit which, in the opinion of the study investigator, are related to administration of the challenge agent.
- Pregnancy outcomes.

  The possible adverse effects of enteric fever infection or the effect of some antibiotics (including azithromycin and ciprofloxacin) on the outcome of pregnancy are unknown. Therefore, pregnant women will be excluded by history and laboratory tests, and female participants will be specifically instructed to prevent conception (see section 8.3). Should pregnancy occur, information about outcome of the pregnancy will be sought.

10.7. Expected Serious Adverse Events and Serious Adverse Reactions

No serious adverse events or reactions are expected. This is addressed in Section Adverse events of special interest (AESI).

10.8. Definition of a Suspected Unexpected Serious Adverse Reaction (SUSAR)

A serious adverse reaction, the nature or severity of which is not consistent with the expected outcomes following challenge with *S.* Typhi or *S.* Paratyphi.

10.9. Procedures for recording Adverse Events

In the 21 days after challenge, solicited symptoms will be recorded once daily by the participant in a study diary (e-diary or paper backup version). All AEs occurring during this time, either observed by the clinical study team or reported by the participant, irrespective of their relatedness to the challenge, medication or procedure will be recorded on the eCRF and graded according to criteria defined in

and APPENDIX **C**: Grading the severity of visit observed Adverse Events~~APPENDIX C: Grading the severity of visit observed Adverse Events~~. In addition participants will be requested to continue to record any unsolicited symptoms until 28 days after challenge in the e-Diary.

AEs will be recorded using the following guidance:

- Pre-existing medical conditions (present before start of the AE collection period) are considered "concurrent medical conditions" and should not be recorded as AEs. However, if the participant experiences a worsening or complication of such a condition, the worsening or complication should

be recorded as an AE. Investigators should ensure that the AE term recorded captures the change in the condition (e.g., "worsening of").

- Each AE should be recorded to represent a **single** diagnosis. Accompanying signs or symptoms (including abnormal laboratory values) should not be recorded as additional AEs.
- Changes in laboratory values are only considered to be AEs if they are judged to be clinically significant, e.g., if some action or intervention is required. If abnormal laboratory values are the result of pathology for which there is an overall diagnosis the diagnosis only should be reported as one AE.
- All AEs that result in a participant's withdrawal from the study or that are present at the end of 28 days post challenge period, will be followed up until a satisfactory resolution occurs or until a non-study related causality is assigned. It will be left to the investigator's clinical judgment whether or not an AE is of sufficient severity to require the participant's removal from treatment. A participant may also voluntarily withdraw from treatment due to what he or she perceives as an intolerable AE. If either of these occurs, the participant must undergo an end of study assessment and be given appropriate care under medical supervision, by referral to their GP, until symptoms cease or the condition becomes resolved or is stable.
- The following information will be recorded in the eCRF: description of the AE, the date of onset and end date, severity, assessment of relatedness to study medication (as judged by a medically qualified investigator) and action taken. Follow-up information should be provided as necessary.

10.10. Procedures for reporting Serious Adverse Events

All serious adverse events (SAE) must be recorded on the Sponsor's SAE form and reported by fax and email to the Sponsor and DSMC Chair within 24 hours of discovery or notification of the event. Additional information received for a case (follow-up or corrections to the original case) need to be detailed on a new SAE form and faxed or emailed to the Sponsor and DSMC Chair, as above.

The chair of the DSMC will perform an independent review of SAEs and request any further information required in a manner adherent to the procedures and timelines of the DSMC Charter. Documentation of this review will be kept in the TMF.

A SAE occurring to a participant should be reported to the REC that gave a favourable opinion of the study where in the opinion of the Chief Investigator the event was 'related' (resulted from administration of any of the research procedures) and 'unexpected' in relation to those procedures. Reports of related and unexpected SAEs should be submitted within 15 working days of the Chief Investigator becoming aware of the event, using the NRES report of serious adverse event form.

10.11. SUSAR Reporting

The Chief Investigator will report all SUSARs to the ethical committee(s) and other parties as applicable. For fatal and life-threatening SUSARS, this will be done no later than 7 calendar days after the Sponsor or delegate is first aware of the reaction. Any additional relevant information will be reported within 8 calendar days of the initial report. All other SUSARs will be reported within 15 calendar days. The Chief Investigator will also inform all investigators concerned of relevant information about SUSARs that could adversely affect the safety of participants.

All SUSARs and deaths occurring during the study will be reported to the Sponsor. For all deaths, available autopsy reports and relevant medical reports will be made available for reporting to the relevant authorities.

10.12. Annual Progress report (APR)

An Annual Progress Report (APR) of the study will be submitted once a year by the Chief Investigator to the Research Ethics Committee. The data lock point will be the anniversary of the favourable opinion of that REC. The APR will be submitted within 30 days from that anniversary date.

10.13. Interim safety review

The first participant to be challenged will be challenged alone. If there are no safety concerns (as assessed by a study doctor and discussed with a senior investigator and or clinician as appropriate) by day 14 post-challenge the remaining participants will be challenged. Any safety concerns that do arise will be referred to the DSMC for review.

10.14. Safety Profile Review

The safety profile will be reviewed frequently by the trial management committee. Any concerns will be referred to the DSMC Chair.

10.15. Trial Management Committee

The study investigators will form the trial management committee and will provide frequent management of the study.

10.16. Data Safety Monitoring Committee (DSMC)

This committee consists of a chairperson and a minimum of two other appropriately qualified members as specified in the relevant SOP.

The DSMC is independent and will review safety data throughout the study according to the DSMC Charter. The DSMC will have access to data and, if required, will monitor these data and make recommendations to the study investigators on whether there are any ethical or safety reasons why the study should not continue. A summary of all AESIs and SAEs to date will be provided to the DSMC on request. Any participant lost to follow up and any withdrawals will also be notified to the DSMC.

The outcome of each DSMC review will be communicated directly to the study investigators and documentation of all reviews will be kept in the TMF. The Chair of the DSMC will also be contacted for advice where the Chief Investigator feels independent advice or review is required.

10.17. Stopping rules

Any safety concerns arising during the study will be discussed with the DSMC.

If the study is halted, the challenge agent will not be administered to any additional participants, and no further procedures will be performed. However, administration of antibiotics and safety follow-up of current participants will continue, overseen by the clinical study team. Enrolment will stop until the DSMC has reviewed the events. Further events and actions to be undertaken will be detailed in the DSMC charter.

10.18. Staff and Investigator safety

All staff working on the project will be required to follow strict infection control techniques as outlined in local SOPs.

11. STATISTICS AND ANALYSIS

11.1. Sample size

A minimum of 40 volunteers will be enrolled into Part A of the study. A minimum of 20 volunteers will be enrolled into Part B . Enrollment is defined as attending the V1 visit and randomisation.

Owing to the window period between V1 and V2 it is anticipated that some volunteers may withdraw consent or be excluded prior to attending challenge. In this instance, these individuals will not be included in the analysis of the primary endpoint (i.e. attack rate calculations). Data from collected up to the point of withdrawal will be used in the analysis of relevant secondary endpoints where appropriate (e.g. laboratory assays). Individuals who withdraw after enrolment, but prior to challenge, will not be replaced.

11.2. Description of Statistical Methods

The analyses for the primary endpoint will be descriptive in nature and will not include formal hypothesis testing.

Binary endpoints for the primary endpoint and reactogenicity endpoints will be described using percentages and 95% binomial exact (Clopper-Pearson) confidence intervals.

Time-to-event endpoints analyses will be conducted using the Kaplan-Meier method and presented as Kaplan-Meier plots.

Immunogenicity data are expected to be skewed and so will be log transformed prior to analysis. Results will be presented as geometric means with 95% confidence intervals. Values below the limit of detection will be replaced by half the value of the lower limit.

The analyses for the exploratory objective to investigate the role of recruitment methods and reasons for volunteer exclusions from typhoid and paratyphoid challenge models will be descriptive.

11.3. Analysis of Outcome Measures/Endpoints

The analysis of the primary endpoint will be descriptive only. The percentage of participants who meet the criteria for diagnosis of enteric fever will be calculated using a 95% Clopper-Pearson Exact confidence interval. The numerator will be the number of participants who meet the criteria for diagnosis and the denominator will include all participants excluding those who withdrew or were treated prior to Day 14 without being diagnosed.

A secondary analysis of the primary endpoint will be conducted using the Kaplan-Meier method which will include all participants. Participants who withdrew or were treated prior to Day 14 and had no diagnosis of enteric fever will be censored in the analysis at the time of withdrawal or treatment. Participants who were not diagnosed with enteric fever and were treated at Day 14 will be counted as ‘events’ occurring at Day 14 in the analysis under the assumption that enteric fever would have occurred had continual monitoring without treatment been possible during the unobserved time period beyond Day 14.

Time-to-event analyses of individual components of the primary outcome (e.g. positive blood culture, oral temperature ≥ 38.0°C etc.) will be conducted using the Kaplan-Meier method and will include all participants. Participants not meeting the criteria for an individual component of the primary endpoint will be censored in the analysis at the time of diagnosis or at Day 14 for those undiagnosed.

11.4. The Level of Statistical Significance

The main study outcome is a proportion, which will be quoted with 95% confidence intervals. 95% confidence intervals will be used throughout.

11.5. Criteria for the Termination of the Study

The Chief Investigator and Data Safety Monitoring Committee will have the right to terminate the study at any time on grounds of participant safety. If the study is prematurely terminated the investigator will promptly inform the participants and will ensure appropriate therapy and follow-up. If the study is halted, the relevant Ethics Committee will be notified within 15 days of this occurring.

11.6. Procedure for Accounting for Missing, Unused, and Spurious Data

All available data will be used in the analyses and there will be no imputations for missing data. Participants will be analysed according to the type of challenge received for summaries by group.

11.7. Inclusion in Analysis

All participants will be included in analysis of the primary endpoint if they are successfully challenged on Day 0 and have at least one post- challenge assessment. If a participant later withdraws from the study, data up until that point will be included in the analysis. Participants who are treated or withdraw before meeting the primary endpoint at day 14 and have no diagnosis of enteric fever prior to day 14 will not be included in the primary analysis of the primary endpoint. Volunteers who are enrolled but withdraw prior to challenge will be included in analysis of relvant secondary/exploratory endpoints where appropriate (e.g. laboratory data collected at primary endoscopy visit).

All participants who have undergone challenge will be included in the time-to-event analysis.

If a participant is inadvertently given an incorrect dose or challenge product they will be analysed according to the dose they received rather than the planned dose.

12. DATA MANAGEMENT

12.1. Access to Data

Direct access will be granted to authorised representatives from the Sponsor or host institution for monitoring and/or audit of the study to ensure compliance with regulations.

Study data will also potentially be accessed and used for future academic research within the OVG.

12.2. Data Recording and Record Keeping

The investigators will populate the content of participants' CRFs, which will be in a paper and/or electronic format using an OpenClinica™ database. This database is stored on a secure University of Oxford server and has restricted access and is password-protected with accountability records. This data includes safety data, laboratory data (both clinical and immunological) and outcome data. All information transcribed to and from the OpenClinica™ database is by encrypted (Https) transfer.

Each study participant will have a unique screening number, which will be allocated following the taking of informed consent, and all names and/or identifying details are not included in any study data electronic file. After enrolment, the participants will be identified by a study specific participants number and/or code. Samples sent to laboratories for processing will be identified by study number and participant number only.

Anonymized CRFs and any other document related to primary and secondary endpoints of the study will also be transmitted to the Sponsor at completion of the study.

12.3. Data integrity

Data collection and storage will be inspected throughout the study by internal (performed by the OVG) and external (by the study Sponsor) monitoring.

12.4. Data archiving and storage

Following completion of the study, all personal data will be transmitted to the Sponsor with an anonymized key and will also be kept for a period of 15 years by Ardington Archives storage (Faringdon, Oxford) according to the latest SOP. Storage of this data will be reviewed every five years and files will be confidentially destroyed if storage is no longer required. Prof Andrew J Pollard, or his successor, as Head of the Oxford Vaccine Group will have the responsibility for custody of the data.

13. QUALITY ASSURANCE PROCEDURES

13.1. Investigator procedures

The study will be conducted in accordance with the current approved protocol, GCP, relevant regulations and standard operating procedures. Approved and relevant Standard Operating Procedures (SOPs) will be used at all clinical and laboratory sites.

13.2. Monitoring

Monitoring will be performed according to Good Clinical Practice (GCP) by CTRG or parties appointed by the Sponsor. Following written SOPs, the monitor(s) will verify that the research study is conducted and data are generated, documented and reported in compliance with the protocol, GCP and the applicable regulatory requirements. The investigator sites will provide direct access to all study related source data/documents and reports for the purpose of monitoring and auditing by the Sponsor and inspection by local and regulatory authorities.

13.3. Source Data

Source documents are original documents, data, and records from which participants' electronic data is directly populated. These include, but are not limited to, hospital records (from which medical history and previous and concurrent medication may be summarized into the electronic data capture database), clinical and office charts, laboratory and pharmacy records, diaries, microfiches, radiographs, and correspondence. In this study electronic data entries will be considered source data where it is the site of the original recording. All documents will be stored safely under strict confidentiality and with restricted access. On all study-specific documents, other than the signed consent and the participant contact sheet, the participant will be referred to by the study participant number/code only.

13.4. Direct access to source data/documents

Direct access will be granted to authorised representatives from the Sponsor, host institution and the regulatory authorities to permit study-related monitoring, audits and inspections.

13.5. Modification to protocol

No amendments to this protocol will be made without consultation with, and agreement of the Sponsor. Any amendments to the study that appear necessary during the course of the study must be discussed by the investigator and Sponsor concurrently. If agreement is reached concerning the need for an amendment, it will be produced in writing by the Chief Investigator and will be made a formal part of the protocol following ethical and regulatory approval.

An administrative change to the protocol is one that modifies administrative and logistical aspects of a protocol but does not affect the participants' safety, the objectives of the study and its progress. An administrative change does not require NHS REC approval.

The Investigator is responsible for ensuring that changes to an approved study, during the period for which NHS REC approval has already been given, are not initiated without NHS REC review and approval except to eliminate apparent immediate hazards to the participant.

13.6. Protocol deviation

Any deviations will be documented in a protocol deviation form and filed in the TMF.

13.7. Audit & Inspection

The Quality Assurance manager operates an internal audit program to ensure that the systems used to conduct clinical research are present, functional and enable research to be conducted in accordance with study protocols and regulatory requirements. Audits include laboratory activities covering sample receipt, processing and storage and assay validation. The internal audits will supplement the external monitoring process and will review processes not covered by the external monitor.

The Sponsor may carry out audit to ensure compliance with the protocol, GCP and appropriate regulations.

13.8. Study Progress

The progress of the study will be overseen by the Chief Investigator.

14. ETHICAL AND REGULATORY CONSIDERATIONS

14.1. Declaration of Helsinki

The Investigator will ensure that this study is conducted in accordance with the principles of the Declaration of Helsinki.

14.2. Guidelines for Good Clinical Practice

The Investigator will ensure that this study is conducted in accordance with relevant regulations and with the Guidelines for Good Clinical Practice.

14.3. Approvals

The protocol, informed consent form, participant information sheet and any proposed advertising material will be submitted to an appropriate Research Ethics Committee (REC), and host institution(s) for written approval.

The Investigator will submit and, where necessary, obtain approval from the above parties for all substantial amendments to the original approved documents.

14.4. Reporting

The CI shall submit once a year throughout the study, or on request, an Annual Progress report to the REC Committee, host organisation and Sponsor. In addition, an End of Study notification and final report will be submitted to the same parties.

14.5. Participant Confidentiality

The study staff will ensure that the participants' anonymity is maintained. Study participants will be identified only by initials and a participants ID number on the CRF and any electronic database. All documents will be stored securely and only accessible by study staff and authorized personnel. The study will comply with the Data Protection Act, which requires data to be anonymized as soon as it is practical to do so.

14.6. Reimbursement

All participants will be reimbursed for their time, travel and for the inconvenience based on the following figures:

- Travel expenses: £15 per visit
- Inconvenience of blood tests: £10 per blood donation
- Time required for visit: £20 per visit
- Time off work compensation: £150 per day
- Endoscopy/Biopsy: £100 per procedure
- ESS: £45 per visit/procedure
- WCE: £45 per visit/procedure

Participants will receive between £3,340 and £3,655 if they remain in the study for the entire period.

Control participants will receive £1,345 if they remain in the study for the entire period.

Payments will be made via bank transfer. Participants will be required to provide banking details including account name, sort code and account number. All personal banking details will be stored confidentially and retained by OVG while the participant is actively involved in the study. Consent will be obtained prior to requesting and storing personal bank account details.

Due to the generous reimbursement for scheduled visits, participants will not be given extra reimbursement for unscheduled visits.

15. FINANCE AND INSURANCE

15.1. Funding

Financial support towards this study has been provided by the Medical Research Council.

15.2. Insurance

The University has a specialist insurance policy in place which would operate in the event of any participant suffering harm as a result of their involvement in the research (Newline Underwriting Management Ltd, at Lloyd’s of London). NHS indemnity operates in respect of the clinical treatment that is provided.

16. PUBLICATION POLICY

The Investigators will be involved in reviewing drafts of the manuscripts, abstracts, press releases and any other publications arising from the study. Authors will acknowledge that the study was funded by the Medical Research Council. Authorship will be determined in accordance with the ICMJE guidelines and other contributors will be acknowledged.

17. REFERENCES

1 Lozano R, Naghavi M, Foreman K, *et al.* Global and regional mortality from 235 causes of death for 20 age groups in 1990 and 2010: a systematic analysis for the Global Burden of Disease Study 2010. *Lancet* 2012; **380**: 2095–128.

2 Clemens J, Favorov M, Ivanoff B, *et al.* Background Paper on Vaccination against Typhoid Fever using New- Generation Vaccines - presented at the SAGE November 2007 meeting Table of Contents. 2007.

3 Crump J a, Mintz ED. Global trends in typhoid and paratyphoid Fever. *Clin Infect Dis* 2010; **50**: 241–6.

4 Waddington CS, Darton TC, Jones C, *et al.* An Outpatient, Ambulant Design, Controlled Human Infection Model Using Escalating Doses of Salmonella Typhi Challenge Delivered in Sodium Bicarbonate Solution. *Clin Infect Dis* 2014; : ciu078 – .

5 Pegues A, Miller S. S. Salmonella Species, including Salmonella Typhi. In: Mandell G, Bennett J, Dolin R, eds. Principles and Practice of Infectious Diseases, 7th edn. Philadelphia: Elsevier Inc., 2013.

6 Meltzer E, Stienlauf S, Leshem E, Sidi Y, Schwartz E. A large outbreak of salmonella paratyphi a infection among israeli travelers to Nepal. *Clin Infect Dis* 2014; **58**: 359–64.

7 Maskey AP, Day JN, Phung QT, *et al.* Salmonella enterica serovar Paratyphi A and S. enterica serovar Typhi cause indistinguishable clinical syndromes in Kathmandu, Nepal. *Clin Infect Dis* 2006; **42**: 1247–53.

8 De Jong HK, Parry CM, van der Poll T, Wiersinga WJ. Host-pathogen interaction in invasive Salmonellosis. *PLoS Pathog* 2012; **8**: e1002933.

9 Bhan MK, Bahl R, Bhatnagar S. Typhoid and paratyphoid fever. *Lancet* 2005; **366**: 749–62.

10 Seth-Smith HMB. SPI-7: Salmonella's Vi-Encoding Pathogenicity Island. *J Infect Dev Ctries* 2008; **2**. DOI:10.3855/jidc.220.

11 Baker S, Sarwar Y, Aziz H, *et al.* Detection of Vi-Negative Salmonella enterica Serovar Typhi in the Peripheral Blood of Patients with Typhoid Fever in the Faisalabad Region of Pakistan Detection of Vi-Negative Salmonella enterica Serovar Typhi in the Peripheral Blood of Patients with Typh. 2005. DOI:10.1128/JCM.43.9.4418.

12 Gal-Mor O, Suez J, Elhadad D, *et al.* Molecular and cellular characterization of a Salmonella enterica serovar Paratyphi a outbreak strain and the human immune response to infection. *Clin Vaccine Immunol* 2012; **19**: 146–56.

13 Raffatellu M, Wilson RP, Winter SE, Bäumler AJ. Clinical pathogenesis of typhoid fever. *J Infect Dev Ctries* 2008; **2**: 260–6.

14 Saul A, Smith T, Maire N. Stochastic simulation of endemic Salmonella enterica serovar Typhi: the importance of long lasting immunity and the carrier state. *PLoS One* 2013; **8**: e74097.

15 Wang H, Dwyer-Lindgren L, Lofgren KT, *et al.* Age-specific and sex-specific mortality in 187 countries, 1970-2010: a systematic analysis for the Global Burden of Disease Study 2010. *Lancet* 2012; **380**: 2071–94.

16 Simanjuntak CH, Paleologo FP, Punjabi NH, *et al.* Oral immunisation against typhoid fever in Indonesia with Ty21a vaccine. *Lancet* 1991; **338**: 1055–9.

17 Lozano R, Naghavi M, Foreman K, *et al.* Global and regional mortality from 235 causes of death for 20 age groups in 1990 and 2010: a systematic analysis for the Global Burden of Disease Study 2010. *Lancet* 2012; **380**: 2095–128.

18 Fraser A, Goldberg E. Vaccines for preventing typhoid fever. *… Database Syst Rev* 2007. http://onlinelibrary.wiley.com/doi/10.1002/14651858.CD001261.pub2/pdf/standard (accessed Dec 17, 2014).

19 Buckle GC, Walker CLF, Black RE. Typhoid fever and paratyphoid fever: Systematic review to estimate global morbidity and mortality for 2010. *J Glob Health* 2012; **2**: 010401.

20 Crump J a, Luby SP, Mintz ED. The global burden of typhoid fever. *Bull World Health Organ* 2004; **82**: 346–53.

21 Baker S, Favorov M, Dougan G. Searching for the elusive typhoid diagnostic. *BMC Infect Dis* 2010; **10**: 45.

22 Liqing Zhou TDCW and AJP. Salmonella - Distribution, Adaptation, Control Measures and Molecular Technologies. InTech, 2012 DOI:10.5772/2470.

23 Corner RJ, Dewan AM, Hashizume M. Modelling typhoid risk in Dhaka metropolitan area of Bangladesh: the role of socio-economic and environmental factors. *Int J Health Geogr* 2013; **12**: 13.

24 Crump JA. Typhoid Fever and the challenge of nonmalaria febrile illness in sub-saharan Africa. *Clin Infect Dis* 2012; **54**: 1107–9.

25 Morpeth SC, Ramadhani HO, Crump JA. Invasive non-Typhi Salmonella disease in Africa. *Clin Infect Dis* 2009; **49**: 606–11.

26 Ochiai RL, Wang X, von Seidlein L, *et al.* Salmonella paratyphi A rates, Asia. *Emerg Infect Dis* 2005; **11**: 1764–6.

27 Sahastrabuddhe S, Carbis R, Wierzba TF, Ochiai RL. Increasing rates of Salmonella Paratyphi A and the current status of its vaccine development. *Expert Rev Vaccines* 2013; **12**: 1021–31.

28 Chandrasiri P, Elwitigala J, G N. A multicentre laboratory study of Gram negative bacterial blood stream infections in Sri Lanka. *Ceylon Med J* 2013; **58**: 56–61.

29 Boggild AK, Castelli F, Gautret P, *et al.* Vaccine preventable diseases in returned international travelers: results from the GeoSentinel Surveillance Network. *Vaccine* 2010; **28**: 7389–95.

30 Travel-associated Illness Trends and Clusters, 2000–2010 - Volume 19, Number 7—July 2013 - Emerging Infectious Disease journal - CDC. .

31 Dongol S, Thompson CN, Clare S, *et al.* The microbiological and clinical characteristics of invasive salmonella in gallbladders from cholecystectomy patients in kathmandu, Nepal. *PLoS One* 2012; **7**: e47342.

32 Gonzalez-Escobedo G, Gunn JS. Gallbladder epithelium as a niche for chronic Salmonella carriage. *Infect Immun* 2013; **81**: 2920–30.

33 Balasegaram S, Potter AL, Grynszpan D, *et al.* Guidelines for the public health management of typhoid and paratyphoid in England: practice guidelines from the National Typhoid and Paratyphoid Reference Group. *J Infect* 2012; **65**: 197–213.

34 Addiman S, Maimaris W, Thomas HL, White G, Lawrence J, Balasegaram S. Public health management of Salmonella Typhi/Paratyphi case and contact screening: lessons from North London. *Public Health* 2013; **127**: 207–13.

35 Humphries RM, Fang FC, Aarestrup FM, Hindler JA. In vitro susceptibility testing of fluoroquinolone activity against Salmonella: recent changes to CLSI standards. *Clin Infect Dis* 2012; **55**: 1107–13.

36 Koirala KD, Thanh DP, Thapa SD, *et al.* Highly resistant Salmonella enterica serovar Typhi with a novel gyrA mutation raises questions about the long-term efficacy of older fluoroquinolones for treating typhoid fever. *Antimicrob Agents Chemother* 2012; **56**: 2761–2.

37 Parry CM, Vinh H, Chinh NT, *et al.* The influence of reduced susceptibility to fluoroquinolones in Salmonella enterica serovar Typhi on the clinical response to ofloxacin therapy. *PLoS Negl Trop Dis* 2011; **5**: e1163.

38 Arjyal A, Basnyat B, Koirala S, *et al.* Gatifloxacin versus chloramphenicol for uncomplicated enteric fever: an open-label, randomised, controlled trial. *Lancet Infect Dis* 2011; **11**: 445–54.

39 Girgis NI, Butler T, Frenck RW, *et al.* Azithromycin versus ciprofloxacin for treatment of uncomplicated typhoid fever in a randomized trial in Egypt that included patients with multidrug resistance. *Antimicrob Agents Chemother* 1999; **43**: 1441–4.

40 Butler T, Sridhar CB, Daga MK, *et al.* Treatment of typhoid fever with azithromycin versus chloramphenicol in a randomized multicentre trial in India. *J Antimicrob Chemother* 1999; **44**: 243–50.

41 Parry CM, Ho VA, Phuong LT, *et al.* Randomized controlled comparison of ofloxacin, azithromycin, and an ofloxacin-azithromycin combination for treatment of multidrug-resistant and nalidixic acid-resistant typhoid fever. *Antimicrob Agents Chemother* 2007; **51**: 819–25.

42 Parry CM, Threlfall EJ. Antimicrobial resistance in typhoidal and nontyphoidal salmonellae. *Curr Opin Infect Dis* 2008; **21**: 531–8.

43 Karki S, Shakya P, Cheng AC, Dumre SP, Leder K. Trends of etiology and drug resistance in enteric fever in the last two decades in Nepal: a systematic review and meta-analysis. *Clin Infect Dis* 2013; **57**: e167–76.

44 Russell F. Antityphoid Vaccination in Children - JAMA 100 YEARS AGO. *JAMA* 2013; **309**: 1758.

45 Organisation WH. Background document : The diagnosis , treatment and Background document : The diagnosis , treatment and. .

46 Sanofi Pasteur MSD. Typhim Vi. 8 Oct. 2012 [letter]. 2012.

47 Wahid R, Simon R, Zafar SJ, Levine MM, Sztein MB. Live oral typhoid vaccine Ty21a induces cross-reactive humoral immune responses against Salmonella enterica serovar Paratyphi A and S. Paratyphi B in humans. *Clin Vaccine Immunol* 2012; **19**: 825–34.

48 McGregor AC, Waddington CS, Pollard AJ. Prospects for prevention of Salmonella infection in children through vaccination. *Curr Opin Infect Dis* 2013; **26**: 254–62.

49 Konadu EY, Lin FC, Hó VA, *et al.* Phase 1 and Phase 2 Studies of Salmonella enterica Serovar Paratyphi A O-Specific Polysaccharide-Tetanus Toxoid Conjugates in Adults , Teenagers , and 2- to 4-Year-Old Children in Vietnam Phase 1 and Phase 2 Studies of Salmonella enterica Serovar Paratyph. 2000. DOI:10.1128/IAI.68.3.1529-1534.2000.Updated.

50 Micoli F, Rondini S, Gavini M, *et al.* O:2-CRM(197) conjugates against Salmonella Paratyphi A. *PLoS One* 2012; **7**: e47039.

51 Safety and Immunogenicity of CVD 1902 Oral Attenuated Vaccine to Prevent S. Paratyphi A Infection - Full Text View - ClinicalTrials.gov. 2010.

52 Hornick RB, Greisman SE, Woodward TE, DuPont HL, Dawkins AT, Snyder MJ. Typhoid fever: pathogenesis and immunologic control. *N Engl J Med* 1970; **283**: 686–91.

53 Hornick R, Greisman S. Typhoid fever: pathogenesis and immunologic control. 2. *New Engl …* 1970; **283**: 739–46.

54 Levine MM, Tacket CO, Sztein MB. Host-Salmonella interaction: human trials. *Microbes Infect*; **3**: 1271–9.

55 Waddington CS, Darton TC, Woodward WE, Angus B, Levine MM, Pollard AJ. Advancing the management and control of typhoid fever: a review of the historical role of human challenge studies. *J Infect* 2014; **68**: 405–18.

56 Darton TC, Blohmke CJ, Pollard AJ. Typhoid epidemiology, diagnostics and the human challenge model. *Curr Opin Gastroenterol* 2014; **30**: 7–17.

57 Ubeda C, Pamer EG. Antibiotics, microbiota, and immune defense. *Trends Immunol* 2012; **33**: 459–66.

58 Kau AL, Ahern PP, Griffin NW, Goodman AL, Gordon JI. Human nutrition, the gut microbiome and the immune system. *Nature* 2011; **474**: 327–36.

59 Reller ME, Olsen SJ, Kressel AB, *et al.* Sexual transmission of typhoid fever: a multistate outbreak among men who have sex with men. *Clin Infect Dis* 2003; **37**: 141–4.

60 Ackers ML, Puhr ND, Tauxe R V, Mintz ED. Laboratory-based surveillance of Salmonella serotype Typhi infections in the United States: antimicrobial resistance on the rise. *JAMA*; **283**: 2668–73.

61 Black RE, Cisneros L, Levine MM, Banfi A, Lobos H, Rodriguez H. Case-control study to identify risk factors for paediatric endemic typhoid fever in Santiago, Chile. *Bull World Health Organ* 1985; **63**: 899–904.

62 Thomas HL, Addiman S, Mellanby A. Evaluation of the effectiveness and efficiency of the public health management of cases of infection due to Salmonella typhi/paratyphi in North East London. *Public Health* 2006; **120**: 1188–93.

63 Braddick MR, Sharp JC. Enteric fever in Scotland 1975-1990. *Public Health* 1993; **107**: 193–8.

64 Zhou L, Pollard AJ. A fast and highly sensitive blood culture PCR method for clinical detection of Salmonella enterica serovar Typhi. *Ann Clin Microbiol Antimicrob* 2010; **9**: 14.

APPENDIX A: Negative control group

A preliminary cohort of three volunteers not previously exposed to *S*. Typhi or *S*. Paratyphi will be recruited to act as negative controls for this study. This group will undergo the same study procedures as those recruited to Part A and B but will not require a screening ultrasound for gallbladder disease or have an ESS or WCE. While this group will not be blinded, they will need to ingest a bicarbonate buffer, complete a course of antibiotics and ideally have two endoscopies with mucosal biopsies. From these three volunteers the same immunological assays as samples from Part A and B will be performed. The aim is to determine if the act of repeated blood sampling, mucosal biopsies and antibiotics have an effect on the systemic and/or mucosal immunological profile. If a positive immunological signal is present, then a further seven volunteers, to a maximum of ten in this negative control cohort, will be recruited.

The study visits for this cohort ends on visit 90 (see table 9).

Interventions (Table 9)

- On challenge day (Day 0), participants will drink two grams of sodium bicarbonate dissolved in 150 ml of distilled water.
- A 14 day antibiotic course will be given at day 7 post-ingestion of bicarbonate buffer.
- From day 7 onwards, participants in the control group will follow the 'post-diagnosis' vist schedule equivalent to those in Part A & B, with the exception of the ED+12hrs vistit. Blood tests from Day 7 onwards will be equivalent to post-diagnosis blood tests for Part A & B .
- e-Diary records will be kept for the purpose of recording symptoms related to procedures and sample collection.
- Specimen collection of stool, blood, and saliva at the same time points as participants in Part A and B will be performed up until day 90.
- Endoscopy visits (Day -180 to day -45) and (Day 21-48) will be performed. The same procedure as outlined in section 8.7 applies.

Table 9. Study procedures and timepoints for negative control group

	Screen	Pre-challenge visits			Challenge Day and Post- Challenge Period (Day 0 – day 90)															
Visit Number	V0	Endo-scopy V1	V2		V04	V05	V06	V07	V08	V09	V10	V11	V12	V13	V14	V15	V16	V27	V28	V29
Visit Time	within 120 days	-180 to -45 days	-7 days		D0	D0+ 12	D1	D2	D3	D4	D5	D6	D7	D8	D9	D10	D11	D28 +/- 4 days	D21 to D48	D90 +/- 14 days
Consent & consent questionnaire	x																			
Continuing/ procedure consent		x	x		x															
Confirm 24 hr contact details	x		x																	
Medical history	x																			
Interim medical history					x	x	x	x	x	x	x	x	x	x	x	x	x	x	x	x
Physical examination[1]	x				x															
Vital signs	x	x	x		x	x	x	x	x	x	x	x	x	x	x	x	x	x	x	x
Urine pregnancy test	x	x																		
Saliva sample					x					x			x				x	x	x	x
Stool sample			x		x			x	x	x	x	x	x	x	x	x	x	x	x	x
Blood sample	x	x			x	x	x	x	x	x	x	x	x	x	x	x	x	x	x	x
12 lead ECG	x																			
Mood assessment	x		x											x						
Endoscopy, fluid aspirate and biopsy		x																	x	
Issue study pack					x															
e-diary entries[2]			x		x	x	x	x	x	x	x	x	x	x	x	x	x	x		
Commence antibiotics													x							
Notification of GP					x															

[1] Physical examination may be performed at other times in the study as clinically indicated.

[2] Solicited responses will be entered from 7 days prior to challenge to Day 21. E-diary will remain open for unsolicited entries until Day 28.

Table 10. Control group sample chart

**Investigation**		Saliva sample	Stool sample	Endoscopy, fluid aspirate & Mucosal biopsy	Commence Antibiotics	Antibody secreting cells	Blood to correlate with Mucosal Biopsy	Whole Blood Stimulation	CyTOF	Cell mediated immunity	Full blood count	CRP, U+Es, LFTs	Virology (HIV, Hep B, Hep C serology)	Coeliac Serology	Serum bactericidal assay	Antibodies	Cytokines	Functional genomics	DNA samples (including epigenetics)	Whole blood assay	Glucose	
**Sample tube**						heparinised falcon (50mL)	Heparinised falcon (15ml)	EDTA vacutainer	EDTA vacutainer	heparinised falcon (50mL)	EDTA vacutainer	Heparin vacutainer	Heparin vacutainer	Heparin vacutainer	Serum vacutainer	Serum vacutainer	Serum vacutainer	Tempus™ Blood RNA	taken from serum samples	heparinised falcon (15 mls)	Glucometer	
**Volume blood (mL)**						20	10 or 20	5	5	50 to 80	1 or 3	2	3	3	3	5 to 10	2	3	x	3	1 drop	
**Visit**	**Time**																					**TOTAL**
VO	**within 120 days**										3	2	3	3							x	11
V1	-180 to -45 days			x			20		5	60												85
V2	-7 days		x								1	2										3
V3	D0	x	x			20		5	5	50	1	2			3	10	2	3	x	3		104
V4	D0+12 hrs																2	3				5
V5	D1		x								1	2					2	3				8
V6	D2		x								1	2				5						8
V7	D3		x														2					2
V8	D4	x	x						5	50	1	2				5		3				66
V9	D5		x														2	3				5
V10	D6		x								1	2					2	3				8
V11	D7 (ED)	x	x		x	20					1	2			3	5	2	3				36
V12	D8 (ED +24)		x						5	50	1	2										58
V13	D9 (ED + 48)		x			20											2					22
V14	D10 (ED + 72)		x								1	2				5	2	3				13
V15	D11 (ED +96)	x	x				10		10	50												70
Volumes per assay if ED						60	10	5	25	200	9	18			6	30	18	24		3		
**Total blood volume in intensive phase**																						**504**
V26	D28	x	x						5	65	1	2				10		3	x			86
V27	D21-48			x			15	3														18
V28	D90	x	x						5	50					3	5		3		3		69
**Total blood volume for duration of study**																						**677**

APPENDIX B: Grading the severity of solicited and unsolicited systemic Adverse Events

Adverse event	Grade	Definition (in degrees Celsius)
Temperature	0	< 37.6
	1	37.6 – 38.0
	2	38.1 – 39.0
	3	> 39.0

Adverse event	Grade	Definition
Any symptom	0	Absence or resolution of symptom
	1	Awareness of symptom but tolerated; transient or mild discomfort; little or no medical intervention required
	2	Discomfort enough to cause limitation of usual activity; some medical intervention or therapy required
	3	Significant interference with daily activity
	4	Emergency department visit or hospitalisation

APPENDIX C: Grading the severity of visit observed Adverse Events

Observation	Grade 1	Grade 2	Grade 3
Oral temperature (°C)	37.6 – 38.0	38.1 – 39.0	39 or greater
Tachycardia (beats/min)	101-115	116-130	>130
Bradycardia (beats/min)	50-54	45-49	<45
Systolic hyper-tension (mmHg)	141-150	151-155	>155
Diastolic hypertension (mmHg)	91-95	96-100	>100
Systolic hypotension (mmHg)	85-89	80-84	<80
Respiratory rate (breaths/min)	17-20	21-25	>25

The following ranges are considered normal physiological ranges and are recorded as Grade 0:

- Oral temperature between 35.5 and 37.5 C
- Resting heart rate between 55 and 100 beats/minute
- Systolic blood pressure between 90 and 140 mmHg
- Diastolic blood pressure below 90 mmHg
- Respiratory rate less than 17 breaths/minute

APPENDIX D: Grading the severity of laboratory Adverse Events

Parameter	Grade 1	Grade 2	Grade 3	Grade 4*
**Haemoglobin: decrease from baseline value (gm/dl)**	< 1.5	1.5-2.0	2.1-5.0	>5
**White cell count: elevated (cell/mm^3)**	10,800–15,000	15,001–20,000	20,001–25,000	>25,000
**White cell count: depressed (cells/mm3)**	2500-3500	1500-2499	1000-1499	<1000
**Neutrophil count (cells/mm^3)**	1500-2000	1000-1499	500-999	<500
**Platelets (cells/mm^3)**	125,000-140,000	100,000-124,000	25,000-99,000	<25,000
**Sodium: hyponatraemia (mEq/L)**	132–134	130–131	125–129	<125
**Sodium: hypernatraemia (mEq/L)**	144–145	146–147	148–150	>150
**Potassium: hyperkalaemia (mEq/L)**	5.1–5.2	5.3–5.4	5.5–5.6	>5.6
**Potassium: hypokalaemia (mEq/L)**	3.5–3.6	3.3–3.4	3.1–3.2	<3.1
**Urea (mmol/L)**	8.2–8.9	9.0–11	>11	RRT
**Creatinine (µmol/L)**	132-150	151-176	177-221	>221 or RRT
**ALT and/or AST (IU/L)**	1.1–2.5 x ULN	>2.6–5.0 x ULN	5.1-10 x ULN	>10 x ULN
**Bilirubin, with increase in LFTs (umol/L)**	1.1–1.25 x ULN	1.26–1.5 x ULN	1.51–1.75 x ULN	>1.75 x ULN
**Bilirubin, with normal LFTs (umol/L)**	1.1–1.5 x ULN	1.6–2.0 x ULN	2.0–3.0 x ULN	>3.0 x ULN
**Alkaline phosphatase (U/L)**	1.1–2.0 x ULN	2.1–3.0 x ULN	3.1–10 x ULN	>10 x ULN
**Amylase (U/L)**	1.1–1.5 x ULN	1.6–2.0 x ULN	2.1–5.0 x ULN	>5.0 x ULN
**Albumin: hypoalbuminaemia (g/L)**	28–31	25–27	<25	Not applicable
**C-reactive protein**	>10-30	31-100	100-200	>200

Grade 4 potentially life threatening*

APPENDIX E: Close Household contacts

Definition of close household contact
Sexual partner
Person sharing bathroom or kitchen with participant
Person for whom the participant may prepare food
Person sleeping in the same bed as the participant

APPENDIX F: Amendment history

Amendment No.	Protocol Version No.	Date issued	Author(s) of changes	Details of Changes made
1	2.0	09-Feb-2015	Malick Gibani	Section 1 - Details of DSMC chair added.<br><br>Section 7.1 – Finalised *S.* Paratyphi challenge dose.<br><br>Section 7.7 – Window period of first endoscopy changed; Time point of wireless capsule endoscopy changed; Changes to symptom recording procedures from Day -7; Addition of food diary; Change to time point of HADS questionnaire.<br><br>Section 8.3 – First line antibiotic changed to Azithromycin.<br><br>Section 9.3 – Changes to PCR methodologies.<br><br>Section 9.8 – Clarification of assay validation procedures.<br><br>Appendix A – Addition of sample chart for negative control group (Table 10).<br><br>Table 1 – Alteration to time points of study procedures (See section 7.7).<br><br>Table 2 – Alteration to sample collection points (Blood to correlate with mucosal biopsy changed to D14 from D6; CMI sample collected at D10 from

				D9). Reduction in salivary sample timepoints; Reduction in PCR time points; Reduction in whole blood assay time points; Reductiion in stool collection time points Addition of RT-PCR; Reduction in volume CMI sample at Day 7; Clarification of ALS sample collection time points, Removal of urine specimen collection; Change to total blood volume collected.
2	3.0	26-MAY-2016	Malick Gibani	**Table 2 -** Changes to sample volumes and time points. Removal of ALS, qPCR and whole blood assay for groups A and B in light of interim data analysis and to re-allocate sample volume for additional exploratory assays. Maximum volume blood sampled over the study period has changed from 1299ml to 1263ml.<br><br>**Section 7.7, Subsection v -** Updated information regarding window periods to study visits.<br><br>**Section 7.7, Subsection vi -** *Updated information regarding window periods to sample processing around time of diagnosis. Changes have been made to facilitate study visits and sample processing.*<br><br>**Section 7.9/Section 8.3 -** Updated guidance regarding collection of clearance stool sample collection. Clearance stool samples for culture will be now obtained one week after completion of the antibiotic course and then every 48 hours until three successive samples are negative

				**Section 9.2 -** The following text has been added: *"Culture based assays to identify cellular localisation of S. Typhi and S.Paratyphi at the time of diagnosis will also be performed in order to further describe the bacterial dynamics after challenge as outlined in the secondary objectives of the study."*<br><br>**Table 10 -** Changes to sample volumes and time points following discussion in laboratory meetings and consensus within the study team. Reduction in volume of CMIs collected at endoscopy 1 visit. Addition of CyTOF sample at Day 90.
3	4.0	28-OCT-2016	Malick Gibani	**Section 2.**<br>Updated information regarding planned sample size updated 40- 60 (Part A) + 20-100 (Part B)<br><br>**Section 5.**<br><br>Updated Part A and PART B planned sample size and volunteer re-recruitment from other studies.<br><br>**Subsection 5.3 Study Overview Figure 1b**<br><br>Updated information regarding planned sample size for a Part B 20-100<br><br>**Subsection 7.1**

				Updated information regarding planned sample size for a Part B 20-100<br><br>**Subsection 7.2**<br><br>Information regarding new types of recruitment methods to be used in this study updated and added<br><br>**Subsection 8.1.**<br><br>'A challenge study using this strain was undertaken at OVG (REC no. 14/SC/0004, OVG 2013/07 Understanding Paratyphoid Infection).[Dobinson et al, in press]. Using the results of the previously performed dose-finding study (OVG 2013/07), the required dose will be administered to provide an inoculum of 1-5x103 CFU. ''<br><br>**Subsection 8.3.**<br><br>''Any participant in whom a contra-indication to Azithromycin becomes apparent (as per The British National Formulary) or if response to treatment is slower than expected, the following regimens of licensed antibiotics will be used:

				2nd line: Oral Ciprofloxacin 500mg twice daily for 14 days,<br>3rd line: Oral trimethoprim/sulfamethoxazole 160/800 mg once daily for 14 days.''
5	5.0	22-MAY-2018-	Malick Gibani	**Section 1**<br>Clarification of sample size: 'Planned Sample Size (Number of participants enrolled)'<br><br>**Section 2**<br>Addition of exploratory objective 'To investigate recruitment methods and reasons for volunteer exclusions from typhoid and paratyphoid challenge models'<br>Analysis of recruitment numbers, including:<br>• number of positive and negative responses to different recruitment techniques;<br>• number of volunteers excluded prior to attending screening visits and reasons for exclusion;

				• number of volunteers attending for screening visits and reasons for exclusion.<br><br>**Section 4**<br>Addition of exploratory objective 'To investigate recruitment methods and reasons for volunteer exclusions from typhoid and paratyphoid challenge models'<br>Analysis of recruitment numbers, including:<br>• number of positive and negative responses to different recruitment techniques;<br>• number of volunteers excluded prior to attending screening visits and reasons for exclusion;<br>• number of volunteers attending for screening visits and reasons for exclusion.<br><br>**Section 5.1**<br>Re-wording of section 5.1 for clarity. 'We will enrol a cohort of 40 to 60 volunteers not

				previously exposed to S. Typhi or S. Paratyphi into Part A’<br><br>**Section 7.6**<br>Re-wording of section 7.6 to clarify definition of enrolment. ‘Enrolment is considered to be when the participant attends V1 and is randomised to S. Typhi or S. Paratyphi.’<br><br>**S**<br>**Section 11.1**<br>Addition of text to clarify sample size:<br>A minimum of 40 volunteers will be enrolled into this arm of the study. Enrolment is defined as attending the V1 visit and randomisation. Owing to the window period between V1 and V2 it is anticipated that some volunteers may withdraw consent or be excluded prior to attending challenge. In this instance, these individuals will not be included in the analysis of the primary endpoint (i.e. attack rate calculations). Data from collected up to the point of withdrawal will be used in the analysis of relevant secondary

				endpoints where appropriate (e.g. laboratory assays). Individuals who withdraw after enrolment, but prior to challenge, will not be replaced.<br><br>**Section 11.7**<br><br>Addition of text to clarify inclusion in analysis. 'Volunteers who are enrolled but withdraw prior to challenge will be included in analysis of relevant secondary/exploratory endpoints where appropriate (e.g. laboratory data collected at primary endoscopy visit).'
